# Dual-Component Nanoemulsion Containing *Brugmansia suaveolens* Leaf Extract and Copaiba Oil: Antimicrobial Activity and Preclinical Wound-Healing Potential

**DOI:** 10.3390/plants15192901

**Published:** 2026-09-23

**Authors:** Sandro Pinheiro da Costa, Verônica da Silva Cardoso, Alane Beatriz Vermelho, Marcel Vasconcellos, Valéria Cristina Lopes Marques, Maria Eduarda Monteiro Silva, Ana Carolina Borba de Frias, Naomi Kato Simas, Ivana Correa Ramos Leal, Maria Helena Amaral, Simone Sacramento Valverde, Eduardo Ricci-Júnior

**Affiliations:** 1 Faculty of Medicine, Centro Universitário Serra dos Órgãos (UNIFESO), Teresópolis 25976-187, RJ, Brazil; sandropinheiropharma@gmail.com (S.P.d.C.); marcelvasconcellos@unifeso.edu.br (M.V.); valeriamarques@unifeso.edu.br (V.C.L.M.); mariaeduardasilva@unifeso.edu.br (M.E.M.S.); carolbfrias08@gmail.com (A.C.B.d.F.); 2 LADEG (Laboratório de Desenvolvimento Galênico e Nanotecnologia), Faculty of Pharmacy, Federal University of Rio de Janeiro (UFRJ), Rio de Janeiro 21941-901, RJ, Brazil; 3 Bioinovar (Unidade de Biocatálise, Bioprodutos e Bioenergia), Paulo de Góes Institute of Microbiology, Federal University of Rio de Janeiro (UFRJ), Rio de Janeiro 21941-901, RJ, Brazil; verocardoso@micro.ufrj.br (V.d.S.C.); abvermelho@micro.ufrj.br (A.B.V.); 4 Laboratorio de Produtos Naturais e Ensaios Biológicos (LAPRONEB), Faculty of Pharmacy, Federal University of Rio de Janeiro (UFRJ), Rio de Janeiro 21941-901, RJ, Brazil; naomisimas@gmail.com (N.K.S.); ivafarma@gmail.com (I.C.R.L.); 5 UCIBIO i4HB Laboratório de Tecnologia Farmacêutica, Faculdade de Farmácia, Universidade do Porto, 4050-313 Porto, Portugal; hamaral@ff.up.pt; 6 Laboratório de Química Medicinal de Produtos Bioativos, Institute os Drug Technology (Farmanguinhos), Fundação Osvaldo Cruz (Fiocruz), Rio de Janeiro 21040-900, RJ, Brazil; simone.valverde@ymail.com

**Keywords:** *Brugmansia suaveolens*, topical nanoemulsion, copaiba oil, wound healing, antimicrobial activity, antioxidant activity, cytocompatibility, tropane alkaloids

## Abstract

This study developed and evaluated a dual-component nanoemulsion containing *Brugmansia suaveolens* (Willd.) Sweet hydroethanolic leaf extract and copaiba oil (BSLE-NE). BSLE and copaiba oil were chemically characterized using high-resolution liquid chromatography–mass spectrometry and gas chromatography–mass spectrometry, respectively. BSLE-NE was optimized and characterized by dynamic light scattering and transmission electron microscopy. The formulation was also subjected to a preliminary 28-day physicochemical stability study at 8, 25, and 40 °C, as well as antimicrobial and cytocompatibility assays and evaluation in an excisional wound model using female *Wistar* rats. The antioxidant activity of BSLE was also assessed. Chemical profiling of BSLE revealed 39 chromatographic features, including putatively annotated tropane alkaloids, whereas β-caryophyllene (48.91%) was the predominant compound in copaiba oil. BSLE-NE exhibited a mean droplet diameter of 144 nm, a polydispersity index of 0.135, and a spherical morphology. The formulation maintained its nanometric droplet size for 28 days at 8 and 25 °C. Both BSLE and BSLE-NE exhibited >90% relative inhibition against *Klebsiella pneumoniae* and *Proteus mirabilis*. At a BSLE-equivalent concentration of 500 μg mL^−1^, nanoemulsification increased J774 macrophage viability from approximately 61% to 84%, while maintaining high viability in fibroblasts and keratinocytes. In vivo, both Copaiba-NE and BSLE-NE improved wound repair relative to the untreated control, while BSLE-NE produced the smallest residual wound diameter and the most advanced tissue organization. These findings support the preclinical wound-repair potential of BSLE-NE as a dual-component topical formulation integrating BSLE and bioactive copaiba oil.

## 1. Introduction

Cutaneous wound healing is a complex and dynamic biological process involving coordinated phases of inflammation, cellular proliferation, and extracellular matrix remodeling. Disruptions in these processes can delay tissue regeneration and increase susceptibility to secondary infections, both of which are commonly associated with chronic wounds [1,2]. The presence of opportunistic pathogens can further sustain inflammation and compromise tissue repair.

Oxidative stress also plays a central role in wound pathophysiology by promoting extracellular matrix degradation and impairing cellular function. These effects underscore the need for therapeutic strategies capable of simultaneously controlling microbial burden and modulating the local redox environment [2]. Accordingly, extracellular matrix components, including structural proteins and polysaccharides, represent important targets for interventions designed to enhance wound healing.

Plant-derived bioactive compounds have been extensively investigated as potential therapeutic agents because of their antioxidant, anti-inflammatory, and antimicrobial properties [3]. In particular, flavonoids and terpenes can modulate inflammatory pathways, attenuate oxidative stress, and promote tissue regeneration [4,5,6]. These compounds may also interact with biological macromolecules involved in skin repair, thereby contributing to the restoration of tissue integrity. Copaiba oil, a natural lipid phase rich in terpenoids, may additionally support the development of topical formulations by facilitating the dispersion of lipophilic compounds and providing a biologically relevant lipid environment for cutaneous delivery [4].

*Brugmansia suaveolens* (Willd.) Sweet (Solanaceae) exhibits a diverse phytochemical profile, with more than 120 reported constituents, including alkaloids, flavonoids, terpenoids, steroids, and other secondary metabolites [7,8]. The tropane alkaloids identified in this species include scopolamine, atropine/hyoscyamine, noratropine, and hydroxylated derivatives [7,8]. Scopolamine and atropine/hyoscyamine act as competitive antagonists of muscarinic acetylcholine receptors, accounting for their established anticholinergic and antispasmodic activities [7,9]. However, excessive exposure to these compounds can also result in central and peripheral anticholinergic toxicity [7,8,9]. Previous investigations have further documented structurally diverse flavonol glycosides in the leaves [10], characterized scopolamine in floral nectar [11], and demonstrated the antinociceptive activity of aqueous flower extracts in experimental models [12].

Over the past decade, nanoemulsions containing plant extracts or essential oils have progressed from primarily formulation-focused investigations to topical delivery systems evaluated for antimicrobial activity and wound repair. Formulations incorporating *Plantago major*, *Achyrocline satureioides*, *Deverra* species, and other plant-derived bioactive compounds have demonstrated formulation-dependent effects on re-epithelialization, inflammation, angiogenesis, tissue organization, and antimicrobial activity [13,14,15,16,17,18,19,20]. Collectively, these findings support the potential of plant-based nanoemulsions as topical delivery platforms while emphasizing that their biological performance depends on formulation composition and processing conditions. Within this framework, the incorporation of *B. suaveolens* extract into a chemically characterized copaiba oil-based nanoemulsion, combined with comprehensive physicochemical and biological assessments, represents the specific contribution of the present study.

Accordingly, this study aimed to develop and characterize a copaiba oil-based nanoemulsion containing *B. suaveolens* leaf extract. The formulation was evaluated in terms of droplet size, pH, and preliminary physicochemical stability over 28 days, in addition to antioxidant and antimicrobial activities, in vivo wound-healing potential, and systemic safety in an experimental animal model.

## 2. Results

### 2.1. Chemical Profiles of Brugmansia suaveolens Leaf Extract and Copaiba Oil

The phytochemical profile of the hydroethanolic leaf extract of *Brugmansia suaveolens* (Willd.) Sweet (BSLE) was qualitatively characterized using ultra-high-performance liquid chromatography coupled with electrospray ionization quadrupole time-of-flight mass spectrometry (UHPLC–ESI–QTOF–MS) in positive-ion mode. The base-peak chromatogram revealed 39 distinct features (Figure 1). Their retention times, peak areas, signal-to-noise ratios, maximum mass-to-charge (*m*/*z*) values, and annotation status are provided in Table S6. Metabolites were tentatively annotated based on their observed *m*/*z* values, chromatographic behavior, mass spectral characteristics, and comparisons with published data. Representative putatively annotated metabolites are summarized in Table 1.

The UHPLC–ESI–QTOF–MS analysis detected 39 chromatographic features across the evaluated retention-time range. Peaks 4–6 were putatively annotated as scopolamine, 3-hydroxy-6-tigloyloxytropane, and noratropine, respectively. The remaining features were retained as unassigned signals because the available full-scan data did not support reliable structural assignments at the confidence level applied. The complete feature-level dataset and annotation status are presented in Table S6.

Gas chromatography–mass spectrometry (GC–MS) analysis of copaiba oil revealed 20 chromatographic components, of which 19 were identified through comparison with the National Institute of Standards and Technology (NIST) mass spectral library, with similarity indices ranging from 90% to 100% (Table S7). β-Caryophyllene was the predominant constituent, accounting for 48.91% of the total chromatographic peak area, followed by germacrene D (7.70%), α-caryophyllene (6.88%), α-copaene (6.33%), α-himachalene (5.88%), and δ-cadinene (4.08%). Collectively, sesquiterpene hydrocarbons and their oxygenated derivatives accounted for 93.17% of the chromatographic profile, confirming the predominantly sesquiterpenic composition of the copaiba oil used as the dispersed phase in BSLE-NE.

### 2.2. In Silico Prediction of Biological Activity and Pharmacokinetic Properties

In silico analyses were performed using scopolamine and noratropine, which were putatively annotated in the BSLE chromatographic profile; hyoscyamine/atropine and 6-hydroxyatropine, which were previously reported for *B. suaveolens*; and β-caryophyllene, the predominant constituent of copaiba oil [7,8]. The Prediction of Activity Spectra for Substances (PASS) Online and SwissADME platforms were used to predict biological activities, physicochemical descriptors, drug-likeness parameters, and properties relevant to topical delivery.

PASS analysis indicated that several compounds exhibited probability of activity (Pa) values exceeding their corresponding probability of inactivity (Pi) values for biological activities associated with wound healing, inflammatory modulation, oxidative stress, and antimicrobial effects (Table S1). Scopolamine exhibited predicted anti-inflammatory (Pa = 0.742) and wound-healing stimulant (Pa = 0.689) activities, whereas hyoscyamine/atropine showed predicted anti-inflammatory (Pa = 0.715) and antibacterial (Pa = 0.621) activities. Noratropine exhibited predicted antimicrobial activity (Pa = 0.598), while 6-hydroxyatropine demonstrated predicted anti-inflammatory activity (Pa = 0.566). β-Caryophyllene showed predicted anti-inflammatory (Pa = 0.854) and antimicrobial (Pa = 0.712) activities, indicating that copaiba oil may contribute to the proposed biological activity of BSLE-NE.

SwissADME predictions indicated that the evaluated compounds encompassed a broad range of physicochemical properties relevant to topical delivery (Table S2). The tropane alkaloids exhibited moderate molecular weights, intermediate lipophilicity, and predicted skin permeability coefficients (log Kp) ranging from −5.62 to −5.81 cm s^−1^, with no violations of Lipinski’s rule of five. β-Caryophyllene exhibited the highest lipophilicity (log P = 4.52 cm s^−1^), a TPSA of 0.0 Å^2^, no violations of Lipinski’s rule of five, and the highest predicted skin permeability (log Kp = −4.62 cm s^−1^). Collectively, these computational findings suggest complementary biological and physicochemical properties among the selected compounds; however, these predictions require experimental confirmation.

### 2.3. Optimization and Physicochemical Characterization of Nanoemulsions

Nanoemulsion optimization was conducted using an experimental design to investigate the effects of formulation composition and ultrasound processing parameters on droplet size distribution and colloidal stability. Response surface analysis demonstrated that the relative proportions of the aqueous and copaiba oil phases directly influenced the polydispersity index (PDI) and mean droplet diameter (Figure 2A–D). Lower PDI values were observed when appropriate proportions of the aqueous Pluronic^®^ F-127 phase and the copaiba oil phase were employed. Conversely, higher oil proportions generally increased droplet diameter and broadened the size distribution, suggesting less effective interfacial stabilization during emulsification (Figure 2A–D).

The optimized formulation, designated BSLE-NE, exhibited a mean droplet diameter of 144 nm and a PDI of 0.135, indicating a nanoscale system with a narrow droplet size distribution, as confirmed by dynamic light scattering (DLS) (Figure S2). Macroscopic examination revealed a homogeneous, pale-yellow dispersion consistent with the formation of an oil-in-water nanoemulsion (Figure S3).

Transmission electron microscopy (TEM) confirmed the presence of predominantly spherical droplets with nanoscale dimensions (Figure 2E,F), consistent with the DLS profile presented in Figure S2. Collectively, these findings support the formation of an oil-in-water nanosystem containing *B. suaveolens* (Willd.) Sweet hydroethanolic leaf extract.

A 28-day stability study was conducted as a preliminary short-term assessment to identify early physicochemical changes in BSLE-NE under different storage temperatures before subsequent biological evaluation. This assessment was not intended to establish the long-term shelf life of the formulation. Throughout the study, BSLE-NE maintained nanoscale droplet diameters under controlled laboratory conditions at 25 ± 0.5 °C and refrigerated storage at 8 ± 0.5 °C. More pronounced changes in PDI were observed in samples stored in a temperature-controlled stability chamber at 40 ± 0.5 °C (Figure 2G,H). The comparatively large SDs observed for PDI at some time points reflect the variability among the triplicate measurements. The pH decreased over time, particularly at later evaluation points (Figure 2I). Nevertheless, pH values remained within a range considered compatible with topical application. No visible creaming or phase separation was observed during the 28-day assessment.

### 2.4. Antimicrobial Activity of BSLE and BSLE-NE

The antimicrobial activities of *B. suaveolens* (Willd.) Sweet hydroethanolic leaf extract (BSLE) and its corresponding nanoemulsion (BSLE-NE) were evaluated against *Staphylococcus aureus*, *Escherichia coli*, *Pseudomonas aeruginosa*, *Klebsiella pneumoniae*, *Proteus mirabilis*, *Candida albicans*, and *Yarrowia lipolytica* (Figure 3). Both samples were tested at an equivalent BSLE concentration of 250 μg mL^−1^, corresponding to 2.5 μg of BSLE per disc, enabling direct comparison between the free and nanoemulsified extract. Representative agar disc-diffusion plates showing BSLE, its matched 70% (*v*/*v*) ethanol vehicle control, and the untreated growth control across all seven tested microorganisms are presented in Figure S4. The 70% (*v*/*v*) ethanol and Copaiba-NE groups were used as the matched controls for BSLE and BSLE-NE, respectively, and their inhibition-zone measurements were incorporated into the control-corrected relative inhibition indices presented in Figure 3. The standardized antimicrobial discs listed in Section 4.10 were used as positive assay controls and were not included in the calculation of the control-corrected relative inhibition indices. Neat copaiba oil was not evaluated as a separate antimicrobial treatment.

Two-way analysis of variance (ANOVA) revealed a significant interaction between formulation and microorganism (*F*_(6,28)_ = 4.149; *p* = 0.0042), indicating that the effect of nanoemulsification depended on the microorganism evaluated. The main effect of microorganism was also significant (*F*_(6,28)_ = 852.4; *p* < 0.0001), confirming substantial differences in susceptibility among the tested strains. In contrast, the overall formulation effect was not significant (*F*_(1,28)_ = 2.372; *p* = 0.1348), indicating comparable overall antimicrobial activity between BSLE and BSLE-NE (Figure 3).

Both BSLE and BSLE-NE exhibited inhibition exceeding 90% against *K. pneumoniae* and *P. mirabilis* (Figure 3). No significant differences between formulations were observed for *K. pneumoniae* (*p* = 0.7273) or *P. mirabilis* (*p* = 0.8248), demonstrating that nanoemulsification preserved the antibacterial activity of BSLE against these Gram-negative strains.

Similarly, BSLE-NE exhibited antimicrobial activity comparable to that of BSLE against *C. albicans*, *E. coli*, and *P. aeruginosa*, with no significant differences between formulations (Figure 3). For *Y. lipolytica*, nanoemulsification significantly reduced antimicrobial activity relative to the free extract (*p* = 0.0278). Conversely, BSLE-NE exhibited significantly greater inhibition of *S. aureus* than BSLE (*p* = 0.0126), although the overall inhibitory response remained lower than that observed against *K. pneumoniae* and *P. mirabilis*.

Collectively, these findings demonstrate that BSLE-NE preserved the antimicrobial activity of BSLE against the most susceptible bacterial strains, while the effects of nanoemulsification varied according to the microorganism evaluated (Figure 3).

### 2.5. 2,2-Diphenyl-1-picrylhydrazyl (DPPH) Radical-Scavenging Activity of BSLE

The antioxidant activity of *B. suaveolens* (Willd.) Sweet hydroethanolic leaf extract was evaluated using the 2,2-diphenyl-1-picrylhydrazyl (DPPH) radical-scavenging assay at concentrations ranging from 5 to 500 μg mL^−1^. Quercetin was used as the reference antioxidant (Figure 4). BSLE exhibited concentration-dependent DPPH radical-scavenging activity, with inhibition increasing from 19.00% at 5 μg mL^−1^ to 70.50% at 500 μg mL^−1^.

Two-way ANOVA revealed a significant interaction between sample and concentration (*F*_(5,12)_ = 12.56; *p* = 0.0002), indicating that differences between BSLE and quercetin varied according to the concentration tested. Significant main effects were also observed for sample (*F*_(1,12)_ = 119.3; *p* < 0.0001) and concentration (*F*_(5,12)_ = 555.7; *p* < 0.0001).

At 500 μg mL^−1^, BSLE exhibited significantly greater radical-scavenging activity than quercetin (70.50% vs. 54.00%; *p* < 0.0001) (Figure 4). Significant differences favoring BSLE were also observed at 50 μg mL^−1^ (24.50% vs. 16.50%; *p* = 0.0012), 25 μg mL^−1^ (21.00% vs. 14.50%; *p* = 0.0062), and 5 μg mL^−1^ (19.00% vs. 13.50%; *p* = 0.0204). No significant differences between BSLE and quercetin were observed at 100 or 200 μg mL^−1^.

Overall, BSLE exhibited measurable concentration-dependent antioxidant activity, with significantly greater DPPH radical scavenging than quercetin at selected concentrations (Figure 4).

### 2.6. Cytotoxicity Evaluation

The cytocompatibility of BSLE, BSLE-NE, Pluronic^®^ F-127 at 10%, and copaiba oil was evaluated in human fibroblasts (HFB), human HaCaT keratinocytes, and murine J774 macrophages. These cell models were selected because they represent distinct and complementary components of cutaneous wound healing. Fibroblasts contribute to extracellular matrix production and tissue remodeling, keratinocytes mediate re-epithelialization and restoration of the epidermal barrier, and macrophages regulate inflammation and facilitate progression to the proliferative phase. Cell viability at the highest concentration evaluated, 500 μg mL^−1^, is presented in Figure 5. According to International Organization for Standardization (ISO) standard 10993-5:2009, materials are considered noncytotoxic when cell viability exceeds 70% relative to the untreated control [21].

In HFB cells, all evaluated samples maintained viability above the ISO-defined threshold (Figure 5A). BSLE resulted in approximately 91% cell viability, whereas BSLE-NE, Pluronic^®^ F-127 at 10%, and copaiba oil maintained viability at approximately 97% or higher.

A similar pattern was observed in HaCaT keratinocytes, with all samples maintaining cell viability above 90% (Figure 5B). BSLE resulted in approximately 94% viability, whereas BSLE-NE, Pluronic^®^ F-127 at 10%, and copaiba oil maintained viability between approximately 96% and 98%.

In J774 macrophages, greater differences in cytocompatibility were observed among the evaluated samples (Figure 5C). BSLE reduced cell viability to approximately 61%, below the 70% threshold established by ISO 10993-5:2009 [21]. In contrast, BSLE-NE maintained macrophage viability at approximately 84%, suggesting that nanoemulsification attenuated the cytotoxicity associated with the free extract in this cell model. Pluronic^®^ F-127 at 10% reduced viability to approximately 59%, whereas copaiba oil maintained viability at approximately 80%.

Overall, BSLE-NE exhibited greater cytocompatibility than BSLE in macrophages while maintaining high viability in fibroblasts and keratinocytes (Figure 5). Based on ISO 10993-5:2009, all tested samples were noncytotoxic to HFB and HaCaT cells at 500 μg mL^−1^. However, BSLE and Pluronic^®^ F-127 at 10% reduced J774 macrophage viability below the recommended 70% threshold [21].

### 2.7. In Vivo Wound Healing Assessment

Macroscopic evaluation revealed progressive wound closure between days 4 and 15 in all experimental groups, with a more pronounced reduction in lesion size in animals treated with BSLE-NE (Figure 6A). Morphometric analysis confirmed a time-dependent decrease in wound diameter in the untreated control, Copaiba-NE, and BSLE-NE groups (Figure 6B). Two-way repeated-measures ANOVA revealed a significant interaction between treatment and time (*F*_(6,27)_ = 23.28; *p* < 0.0001), as well as significant main effects of time (*F*_(1.735,15.62)_ = 2716; *p* < 0.0001) and treatment (*F*_(2,9)_ = 70.98; *p* = 0.0001). On day 15, BSLE-NE treatment resulted in the smallest residual wound diameter, approximately 0.025 mm, which was significantly lower than that observed in the untreated control group, approximately 1.200 mm (*p* = 0.0038), and the Copaiba-NE group, approximately 0.425 mm (*p* = 0.0095). Copaiba-NE also significantly reduced wound diameter compared with the untreated control on day 15 (*p* = 0.0078) (Figure 6B). These findings indicate that both nanoemulsion formulations promoted wound closure, with BSLE-NE producing the greatest improvement under the experimental conditions evaluated.

### 2.8. Histopathological Evaluation of Wound Healing

Histopathological evaluation was performed on day 15 after wound induction using hematoxylin-and-eosin-stained skin sections. Representative sections from the untreated control and BSLE-NE groups are presented in Figure 7, illustrating differences in epithelial organization and granulation tissue maturation. The untreated control group exhibited less organized tissue architecture and less advanced structural features of repair (Figure 7A). In contrast, the BSLE-NE group exhibited more advanced epithelial organization and better-structured granulation tissue, consistent with improved tissue repair on day 15 (Figure 7B). Additional histopathological images comparing the untreated control, Copaiba-NE, and BSLE-NE groups are provided in Figure S1.

Semiquantitative scoring further demonstrated greater tissue organization in the BSLE-NE group (Table S5). All groups achieved complete re-epithelialization, corresponding to a score of 2. However, BSLE-NE achieved the highest total histopathological score, 11 out of 12, followed by Copaiba-NE, 8 out of 12, and the untreated control, 5 out of 12. The BSLE-NE group exhibited higher scores for keratinization, granulation tissue organization, scar elevation index, and tissue remodeling, indicating more advanced tissue maturation on day 15. Copaiba-NE also improved selected histological parameters relative to the untreated control, particularly epidermal thickness and keratinization, but achieved lower scores than BSLE-NE for granulation tissue organization and remodeling.

### 2.9. Biochemical and Hematological Evaluation

Biochemical and hematological parameters were assessed on day 15 following topical treatment with Copaiba-NE or BSLE-NE (Tables S3 and S4). ALT and AST, evaluated as serum indicators of hepatocellular injury, did not exceed the published reference intervals for female Wistar rats of comparable age [22]. Alanine aminotransferase (ALT) levels were 26.0 U L^−1^ in the Copaiba-NE group and 22.0 U L^−1^ in the BSLE-NE group, whereas aspartate aminotransferase (AST) levels were 68.0 and 71.5 U L^−1^, respectively. Creatinine concentrations ranged from 0.40 to 0.64 mg dL^−1^, and urea concentrations ranged from 30.8 to 42.6 mg dL^−1^ (Table S3).

Hematological evaluation revealed broadly comparable profiles between the Copaiba-NE and BSLE-NE groups (Table S4). Erythrocyte-related parameters, including red blood cell count, hemoglobin concentration, and hematocrit, remained within or close to their respective reference ranges, as did platelet counts. However, total leukocyte counts (reference range: 4000–12,000 cells μL^−1^), neutrophil counts (1200–5400 cells μL^−1^), and lymphocyte counts (4800–9840 cells μL^−1^) were below their respective reference intervals in both treated groups. Although these reductions were not accompanied by biochemical abnormalities or clinical signs of systemic toxicity, they warrant cautious interpretation and further investigation.

Overall, topical administration of BSLE-NE was not associated with detectable alterations in hepatic or renal biochemical markers relative to Copaiba-NE under the experimental conditions evaluated. However, the reduced leukocyte, neutrophil, and lymphocyte counts observed in both treatment groups preclude definitive conclusions regarding systemic safety and indicate the need for additional hematological assessment (Tables S3 and S4).

## 3. Discussion

### 3.1. Chemical Profiles of Brugmansia suaveolens Leaf Extract and Copaiba Oil

Three chromatographic features were putatively annotated as tropane alkaloids in the *Brugmansia suaveolens* (Willd.) Sweet hydroethanolic leaf extract (BSLE) profile. Scopolamine, 3-hydroxy-6-tigloyloxytropane, and noratropine were provisionally assigned based on their chromatographic behavior and mass spectral characteristics. These alkaloids are characteristic constituents of species belonging to the Solanaceae family and are associated with diverse pharmacological activities, providing a relevant framework for interpreting the antimicrobial and cell-type-dependent responses observed in the present study [7,8]. However, because these assignments were based on qualitative chromatographic and mass spectrometric analyses, definitive structural confirmation and quantification will require complementary analytical approaches and comparison with authentic reference standards. Overall, UHPLC–ESI–QTOF–MS provided a qualitative feature profile of BSLE, with compound-level assignments restricted to the three supported putative annotations.

GC–MS analysis demonstrated that copaiba oil was composed predominantly of sesquiterpenes, with β-caryophyllene accounting for 48.91% of the total chromatographic peak area. This value closely agrees with a published GC–MS profile of copaiba oil containing 48.29% β-caryophyllene [23]. Moreover, the concurrent detection of β-caryophyllene, α-copaene, α-caryophyllene (α-humulene), γ-muurolene, and β-bisabolene in the analyzed batch is consistent with the characteristic sesquiterpene profile reported for authenticated copaiba oil-resin samples [24]. Together, these findings demonstrate that the sesquiterpene composition of the analyzed batch is consistent with published copaiba oil profiles [23,24].

Previous experimental studies have indicated that β-caryophyllene can promote cellular migration and re-epithelialization while modulating inflammatory responses during cutaneous wound repair [25]. The presence of other constituents, particularly germacrene D, α-himachalene, and δ-cadinene, further demonstrates the chemical complexity of the oil phase. Accordingly, BSLE-NE is best interpreted as a dual-component nanoemulsion in which copaiba oil functions both as the dispersed phase and as a source of bioactive sesquiterpenes, while BSLE provides a complementary phytochemical contribution. This interpretation is consistent with the biological response observed for Copaiba-NE and the additional improvement produced by BSLE-NE.

### 3.2. In Silico Prediction of Biological Activity and Pharmacokinetic Properties

The in silico findings indicated complementary predicted biological and physicochemical properties among the evaluated tropane alkaloids and β-caryophyllene. The tropane alkaloids exhibited predicted activities associated with inflammatory modulation and antimicrobial effects, whereas β-caryophyllene combined predicted anti-inflammatory and antimicrobial activities with pronounced lipophilicity.

The predicted physicochemical differences among the selected compounds suggest that their behavior within the formulation and at the skin surface may vary. The tropane alkaloids exhibited intermediate lipophilicity and predicted skin permeability, whereas the high lipophilicity and low polar surface area of β-caryophyllene were consistent with greater predicted skin permeability.

These computational findings should be interpreted as hypothesis-generating rather than as direct evidence of biological activity, cutaneous absorption, or therapeutic mechanisms. Hyoscyamine/atropine and 6-hydroxyatropine were included as literature-derived representatives and were not analytically confirmed in the BSLE batch evaluated in this study [7,8]. Accordingly, the predicted activities require validation through targeted chemical analysis, biological assays, and skin permeation studies.

### 3.3. Optimization and Physicochemical Characterization of Nanoemulsions

The physicochemical characteristics of BSLE-NE reflect the effectiveness of high-energy emulsification by probe ultrasonication. In this process, acoustic cavitation generates intense local shear forces that disrupt larger oil droplets into nanoscale structures. The final droplet size depends on the balance between droplet disruption and recoalescence, which is influenced by energy input, formulation composition, and the rapid adsorption of surfactant molecules at the oil–water interface [26,27,28].

The low PDI observed for BSLE-NE indicates a relatively homogeneous droplet population, suggesting that droplet disruption and interfacial stabilization were sufficient to limit recoalescence during processing (Figure 2A–D and Figure S2). Pluronic^®^ F-127 likely contributed to this behavior by forming a stabilizing interfacial layer around the dispersed droplets. Adequate surfactant coverage can reduce droplet aggregation and limit instability processes, including flocculation, coalescence, and Ostwald ripening, particularly when the droplet size distribution remains narrow [14,15].

The 28-day stability assessment further demonstrated that storage temperature influenced the physicochemical behavior of the formulation. Higher temperatures may increase droplet mobility and collision frequency, thereby favoring broader size distributions and progressive destabilization. The more pronounced changes in PDI observed at 40 ± 0.5 °C are consistent with this interpretation, whereas the maintenance of nanoscale droplet diameters under laboratory and refrigerated conditions supports short-term colloidal stability (Figure 2G,H). The progressive reduction in pH may reflect chemical changes within the formulation, including hydrolytic or oxidative processes involving plant-derived constituents or components of the oil phase (Figure 2I). However, these mechanisms were not directly investigated and therefore remain speculative. Although the pH values remained compatible with topical application, longer-term stability studies are necessary to determine whether these changes affect formulation performance, chemical composition, or shelf life.

The small droplet diameter and relatively uniform size distribution of BSLE-NE increase the interfacial area available for contact with the skin surface. This characteristic may facilitate the dispersion of lipophilic constituents and support their interaction with the stratum corneum, potentially influencing local retention and partitioning into superficial skin layers. Nanoemulsions are frequently investigated for topical delivery because they can improve the physicochemical dispersion of poorly water-soluble compounds while maintaining formulation homogeneity [14,15,16,20]. Nevertheless, enhanced cutaneous penetration or tissue retention cannot be inferred solely from droplet size and must be verified experimentally.

Collectively, these findings support BSLE-NE as a dual-component nanoscale system integrating the chemically characterized copaiba oil phase with *B. suaveolens* hydroethanolic leaf extract, while highlighting the need for extended stability and skin-delivery investigations.

### 3.4. Antimicrobial Activity of BSLE and BSLE-NE

The pronounced inhibitory activity of BSLE and BSLE-NE against *K. pneumoniae* and *P. mirabilis* is relevant to wound management because these microorganisms may contribute to persistent infection, biofilm formation, prolonged inflammation, and delayed tissue repair. The observed responses indicate that BSLE contains constituents capable of inhibiting these bacteria under the experimental conditions employed.

The maintenance of antibacterial activity following nanoemulsification suggests that the formulation process did not substantially compromise the antimicrobial properties of BSLE against the most susceptible strains. This finding is particularly relevant because nanoemulsions can improve the dispersion of plant-derived constituents with different polarity profiles and may facilitate their interaction with microbial surfaces in topical systems [16,17]. However, because antimicrobial activity was assessed using an agar diffusion method, the measured inhibitory response may reflect both intrinsic antimicrobial activity and the diffusion characteristics of the formulation through the agar matrix.

The lower activity of BSLE-NE against *Y. lipolytica* suggests that nanoemulsification altered the apparent availability of active constituents against this microorganism. One possible explanation is that partitioning of extract components into the oil phase or surfactant-rich interfacial region reduced their immediate release and diffusion through the agar. Alternatively, yeast-specific differences in cell envelope composition and susceptibility may have contributed to the observed response. These possibilities require confirmation through complementary assays, including broth microdilution, time-kill studies, and formulation release experiments.

Conversely, the greater inhibition of *S. aureus* by BSLE-NE suggests that nanoemulsification may have improved the accessibility or dispersion of constituents active against this organism. Increased interfacial contact between nanoemulsion droplets and bacterial cells or improved distribution of lipophilic compounds could contribute to this effect. Nevertheless, these proposed mechanisms were not directly evaluated, and the absolute susceptibility of *S. aureus* remained lower than that of *K. pneumoniae* and *P. mirabilis*.

The microorganism-dependent activity observed for BSLE and BSLE-NE is consistent with the chemically complex nature of the extract and formulation. Because the individual BSLE constituents were not isolated or evaluated separately, the antimicrobial findings are interpreted at the whole-extract and formulation levels [8,29].

The substantial inhibition of *K. pneumoniae* and *P. mirabilis* is noteworthy because the outer membrane and efflux systems of Gram-negative bacteria can limit the intracellular accumulation of antimicrobial compounds. The preservation of activity following incorporation into BSLE-NE indicates that the nanoemulsion maintained the functional antimicrobial response of the extract and may have supported the dispersion of bioactive constituents [16,17].

Overall, the findings support the antimicrobial potential of BSLE-NE as a topical formulation while demonstrating that the effects of nanoemulsification vary according to the microorganism evaluated. Further studies using quantitative susceptibility methods, clinically relevant isolates, and biofilm models are required to define its antimicrobial scope more precisely.

### 3.5. DPPH Radical-Scavenging Activity of BSLE

The antioxidant activity of BSLE is relevant to cutaneous wound repair because excessive oxidative stress can prolong inflammation, impair fibroblast and keratinocyte function, and promote extracellular matrix degradation [1,2]. The concentration-dependent DPPH radical-scavenging activity observed in this study indicates that BSLE contains constituents capable of reducing a stable free radical under the conditions of this chemical assay.

The greater DPPH inhibition observed for BSLE than for quercetin at selected concentrations may reflect the combined effects of multiple redox-active constituents within the extract. Nevertheless, comparisons between a chemically complex extract and an isolated reference compound should be interpreted cautiously because differences in molecular composition, concentration basis, solubility, and assay behavior can influence the apparent response. Furthermore, DPPH scavenging does not directly establish antioxidant activity in living cells or wound tissue.

From a formulation perspective, the presence of antioxidant activity in BSLE supports its investigation as a component of a topical delivery system. Nanoemulsions have been studied as platforms for improving the dispersion, protection, and local availability of plant-derived bioactive compounds in cutaneous applications [14,15,16,17]. These properties are consistent with the nanoscale droplet size, low PDI, and preliminary short-term stability observed for BSLE-NE. However, because antioxidant activity was assessed for the free extract rather than the nanoemulsion, preservation or enhancement of antioxidant activity following formulation cannot be established from the present results.

Accordingly, the DPPH findings support the biological rationale for incorporating BSLE into a topical formulation, while additional cell-based oxidative stress assays and direct evaluation of BSLE-NE are needed to establish its antioxidant relevance to wound healing.

### 3.6. Cytotoxicity Evaluation

The preservation of HFB viability is relevant to wound repair because fibroblasts play essential roles in extracellular matrix production, collagen deposition, granulation tissue formation, and wound contraction [1,2]. Both BSLE and BSLE-NE maintained fibroblast viability above the threshold defined by ISO 10993-5:2009, indicating acceptable cytocompatibility in this cell model under the evaluated conditions [21]. The numerically higher viability observed with BSLE-NE suggests that nanoemulsification may have modified cellular exposure to extract constituents, although the mechanism underlying this effect was not directly investigated. Similar changes in the interaction between bioactive compounds and cells have been described for nanostructured delivery systems [16,17].

The maintenance of HaCaT keratinocyte viability further indicates that incorporation of BSLE into the nanoemulsion did not increase cytotoxicity toward epithelial cells. This observation is relevant because keratinocytes are essential for re-epithelialization and restoration of the epidermal barrier during wound closure [1,2]. The favorable viability profile of BSLE-NE is consistent with reports indicating that nanocarrier systems can improve the dispersion of plant-derived compounds while maintaining epithelial cell compatibility [16,18].

The most pronounced formulation-dependent difference was observed in J774 macrophages. Whereas free BSLE reduced viability below the 70% threshold established by ISO 10993-5:2009, BSLE-NE maintained macrophage viability above this limit [21]. This result suggests that nanoemulsification attenuated the cytotoxic response of the extract in this immune cell model. Possible explanations include altered partitioning of bioactive constituents, reduced direct exposure to free compounds, and changes in their interaction with the cellular interface. However, controlled release, intracellular uptake, and the mechanisms responsible for the improved viability were not directly assessed and therefore cannot be confirmed [17,18].

Macrophage compatibility is particularly relevant because these cells regulate inflammatory signaling, removal of cellular debris, and progression from the inflammatory to the proliferative phase of wound healing [1,2]. Accordingly, the improved viability observed with BSLE-NE may be advantageous for preserving cellular functions associated with tissue repair. Nevertheless, viability alone does not establish that macrophage inflammatory activity, polarization, or phagocytic function remains unaffected.

The reduced viability associated with Pluronic^®^ F-127 at 10% in J774 cells also indicates that formulation components can exhibit cell-type-dependent effects. However, the response to an isolated component cannot be directly extrapolated to its behavior within the complete nanoemulsion because concentration, molecular organization, and interactions with other formulation constituents may differ.

Overall, the cytotoxicity findings support the improved compatibility of BSLE-NE with macrophages and its favorable profile in fibroblasts and keratinocytes. Additional investigations addressing concentration–response relationships, inflammatory biomarkers, repeated exposure, and primary human cell models would further clarify the biological safety of the formulation [16,17,18,21].

### 3.7. In Vivo Wound-Healing Assessment

The accelerated reduction in wound diameter observed with BSLE-NE indicates that the formulation promoted cutaneous wound closure under the experimental conditions evaluated. The superior response relative to both the untreated control and Copaiba-NE suggests that incorporation of BSLE contributed additional biological activity beyond that associated with the copaiba oil-based vehicle. Nevertheless, the improvement observed with Copaiba-NE also indicates that the vehicle itself was biologically active, consistent with the recognized wound-healing and anti-inflammatory potential of copaiba oil constituents, particularly β-caryophyllene [25].

The observed wound-healing response may reflect the combined contributions of antimicrobial activity, antioxidant potential, favorable cytocompatibility, and the physicochemical characteristics of the nanoemulsion. These properties are relevant to the control of microbial burden, regulation of oxidative stress, preservation of repair-associated cells, and local delivery of bioactive compounds [1,2,16,17]. However, the study was not designed to distinguish the relative contribution of each process or to establish a specific mechanism of action.

Interpretation of the animal model also requires consideration of interspecies differences in wound repair. Rodent skin contains the *panniculus carnosus* muscle, which contributes substantially to wound contraction and differs from the predominantly re-epithelialization-driven healing process observed in human skin [30]. Consequently, the reduction in wound diameter observed in rats cannot be directly extrapolated to clinical efficacy in humans.

The experimental design compared BSLE-NE with an untreated control and a corresponding copaiba oil nanoemulsion without BSLE, enabling assessment of the extract-containing formulation relative to its vehicle. However, a dose-matched group treated with non-formulated BSLE was not included. Therefore, although the results demonstrate that BSLE-NE was more effective than the corresponding vehicle, they do not establish whether nanoemulsification improved wound healing compared with the free extract.

Future investigations should incorporate non-formulated BSLE, additional dose levels, quantitative wound area measurements, and extended follow-up to clarify the specific contribution of nanoemulsification and to better characterize the therapeutic potential of the formulation.

### 3.8. Histopathological Evaluation of Wound Healing

The histopathological findings complement the morphometric results by indicating that BSLE-NE improved not only the rate of wound closure but also the structural organization of the repaired tissue. Although complete re-epithelialization was observed in all groups by day 15, the higher histopathological score in the BSLE-NE group reflected more advanced keratinization, granulation tissue organization, scar characteristics, and tissue remodeling. These findings suggest that the formulation promoted a more mature repair response than either the untreated control or Copaiba-NE.

The improved tissue organization observed after BSLE-NE treatment is interpreted at the level of the dual-component formulation. Because the individual BSLE constituents and the proposed wound-repair mechanisms were not directly evaluated, compound-specific attribution is not supported by the present dataset.

The favorable histopathological response may also reflect the physicochemical characteristics of the nanoemulsion. Nanoscale delivery systems can improve the dispersion of plant-derived constituents and potentially influence their local availability at the wound surface, thereby supporting interactions with the repairing tissue [16,17]. Nevertheless, because cutaneous permeation, tissue retention, and release kinetics were not investigated, the contribution of enhanced local delivery remains unconfirmed.

Previous investigations of *B. suaveolens* leaf extracts have reported chemical diversity, antioxidant and antimicrobial activities, and cell-type-dependent cytotoxicity [8]. These observations support the rationale for examining the extract within a topical delivery platform. Similarly, nanoemulsions containing *Plantago major* and *Achyrocline satureioides* extracts have demonstrated formulation-dependent effects on re-epithelialization, inflammation, angiogenesis, and histological organization [13,17]. The present study extends this body of evidence by incorporating BSLE into a copaiba oil-based nanoemulsion and integrating physicochemical characterization with antimicrobial, cytocompatibility, morphometric, and histopathological assessments.

Despite these encouraging findings, histopathological interpretation should consider the semiquantitative nature of the scoring system and the single evaluation time point. Additional studies incorporating blinded quantitative histomorphometry, collagen-specific staining, angiogenesis markers, inflammatory mediators, and serial tissue sampling would provide a more detailed understanding of the mechanisms and temporal progression of tissue repair.

### 3.9. Biochemical and Hematological Evaluation

The biochemical findings did not indicate marked hepatic or renal alterations following topical exposure to Copaiba-NE or BSLE-NE under the experimental conditions evaluated. Creatinine and urea values remained within or close to the reported physiological reference ranges, suggesting preserved renal function, whereas ALT and AST values did not demonstrate a pattern consistent with overt hepatocellular injury. Thus, the available in vivo biochemical data did not indicate detectable hepatocellular injury after 15 days of topical treatment under the evaluated conditions.

However, interpretation of the hematological results requires greater caution. Total leukocyte, neutrophil, and lymphocyte counts were below their respective reference intervals in both treatment groups. Although this pattern occurred in animals treated with either Copaiba-NE or BSLE-NE and was not accompanied by biochemical abnormalities or reported clinical signs of systemic toxicity, the observed reductions cannot be dismissed as biologically irrelevant. In the absence of appropriate baseline measurements, an untreated hematological control, longitudinal monitoring, and statistical comparisons, it is not possible to determine whether these alterations reflect treatment exposure, physiological variability, experimental handling, or other factors.

Accordingly, the available data do not support definitive conclusions regarding systemic hematological safety. Instead, they indicate that BSLE-NE was not associated with clear hepatic or renal biochemical abnormalities relative to Copaiba-NE while highlighting an unresolved leukocyte-related finding that warrants further investigation. Although topical nanostructured systems are generally designed to favor local delivery and limit nonspecific systemic exposure, such properties cannot be assumed without direct assessment of systemic absorption and toxicological outcomes [16,17].

The present study was designed as an integrated preliminary evaluation of BSLE-NE, combining a 28-day physicochemical stability assessment with a 15-day wound-healing model. Future investigations should include extended stability studies, dose-matched comparisons with non-formulated BSLE, cutaneous permeation and tissue-retention experiments, comprehensive hematological monitoring, and organ-relevant hepatic safety assessments, including HepG2 cytotoxicity and liver histopathology. These additional studies will be necessary to define the formulation-specific contribution of nanoemulsification and establish the safety and translational potential of BSLE-NE more rigorously.

## 4. Materials and Methods

### 4.1. Plant Material and Extraction

Leaves of Brugmansia suaveolens (Willd.) Sweet were collected during the flowering period, in April and September 2024, at Serra dos Órgãos National Park (PARNASO), Teresópolis, RJ, Brazil (895 m; 22°26′09.38″ S, 42°58′33.22″ W), under authorization from the Brazilian National System for the Management of Genetic Heritage and Associated Traditional Knowledge (SISGEN; registration no. A76B905).

Fresh leaves were cleaned, manually cut into fragments of approximately 2 cm in their largest dimension, and processed within 2 h of collection. The hydroethanolic leaf extract was obtained by dynamic cold maceration for seven days at room temperature in amber glass containers, using ethanol–water (70:30, *v*/*v*) as the extraction solvent. The macerate was manually homogenized once every 24 h by shaking the containers for approximately 1 min. A solvent-to-plant material ratio of 4 L per 1 kg of leaves was employed.

After extraction, the hydroethanolic solution was filtered by gravity through filter paper using a funnel and subsequently concentrated under reduced pressure using a rotary evaporator (Tecnal^®^, Piracicaba, Brazil) at 50 °C. From 1 kg of fresh leaves, approximately 53 g of concentrated extract was obtained, corresponding to an extraction yield of 5.3% (*w*/*w*) relative to the initial fresh plant mass. The concentrated extract was stored at 4 °C until use. This extract was designated as *B. suaveolens* hydroethanolic leaf extract (BSLE) and was used for the development of the nanoemulsion system evaluated in this study.

### 4.2. Chemical Characterization of BSLE and Copaiba Oil

#### 4.2.1. UHPLC–ESI–QTOF–MS Analysis

The phytochemical profile of *B. suaveolens* hydroethanolic leaf extract (BSLE) was determined by ultra-high-performance liquid chromatography coupled with electrospray ionization quadrupole time-of-flight mass spectrometry (UHPLC–ESI–QTOF–MS), using a Shimadzu Nexera system (Japan) coupled to a Bruker Compact mass spectrometer. Chromatographic separation was performed on an ALLTECH C18 column (50 × 2.1 mm, 3.0 μm) at 30 °C, using water containing 0.1% formic acid (A) and acetonitrile containing 0.1% formic acid (B) as the mobile phase, at a flow rate of 0.4 mL min^−1^. For UHPLC–ESI–QTOF–MS analysis, a BSLE stock solution was prepared at 20 mg mL^−1^ in methanol–water (80:20, *v*/*v*), diluted 1:10 to a final concentration of 2 mg mL^−1^, and filtered through a 0.22 μm membrane before injection. The commercial scopolamine butylbromide reference solution used for qualitative comparison had a concentration of 20 mg mL^−1^ (Hipolabor, Belo Holizonte, Brazil).

The gradient program was as follows: 0–2 min, 5% B; 2–17 min, 5–95% B; 17–19 min, 95% B; 19–19.5 min, return to 5% B; and 19.5–21 min, re-equilibration. The injection volume was 2 μL, and analyses were performed in triplicate.

Detection was carried out in positive ion mode with a capillary voltage of 4.5 kV, gas temperature of 220 °C, nitrogen flow of 10 L min^−1^, and nebulizer pressure of 5.0 bar. Data were acquired in full-scan mode over an *m*/*z* range of 50–800.

Putative metabolite annotation was based on the accurate masses of protonated molecular ions ([M + H]^+^), full-scan mass spectral information, retention time, and comparison with spectral databases, PubChem records, and literature data.

#### 4.2.2. GC–MS Analysis

The chemical composition of copaiba oil, selected as the oil phase for the development of the BSLE-loaded nanoemulsion (BSLE-NE), was determined by gas chromatography coupled to mass spectrometry (GC-MS). This analysis was performed to characterize the lipid component of the formulation and to support the use of copaiba oil as a functional oil phase for topical delivery.

Copaiba oil (*Copaifera* sp.) was supplied by the Galenic Development Laboratory, Federal University of Rio de Janeiro (UFRJ, Brazil). The batch used as the oil phase of BSLE-NE was the same batch previously characterized by GC–MS. The analysis was performed using a Shimadzu GC-MS QP2010 Plus^®^ system (Japan) equipped with an AOC-20i autosampler. Chromatographic separation was carried out on a 5% phenyl-methylpolysiloxane capillary column (29.6 m × 0.25 mm × 0.25 μm), using helium as the carrier gas.

The oven temperature program was set at 60 °C for 1 min, followed by a ramp of 10 °C min^−1^ up to 250 °C, which was held for 3 min. The injector temperature was maintained at 250 °C.

For sample preparation, 30 mg of copaiba oil was diluted in 2 mL of ethyl acetate (Merck, Darmstadt, Germany) and placed in an ice bath, after which 2 mL of a 0.4 M trimethylsilyl (TMS) reagent solution in ethyl acetate was slowly added. After derivatization, the solvent was evaporated under a nitrogen stream, and the resulting sample was injected into the GC-MS system.

Compound identification was performed by comparing the obtained mass spectra with those available in the NIST library, considering a similarity index ≥ 90%. The compounds identified in copaiba oil were subsequently considered in the formulation rationale, since this oil constituted the dispersed lipid phase of BSLE-NE.

### 4.3. In Silico Analysis

Representative compounds from the UHPLC–ESI–QTOF–MS profile of BSLE, metabolites previously reported for *B. suaveolens*, and the GC–MS profile of copaiba oil were selected for computational screening to provide chemically relevant representatives of the extract and oil phase.

Scopolamine and noratropine were selected from the putatively annotated BSLE metabolites; hyoscyamine/atropine and 6-hydroxyatropine were included as representative metabolites previously reported for *B. suaveolens* (Willd.) Sweet [7,8]; and β-caryophyllene was selected as the major constituent identified in copaiba oil. Molecular structures were retrieved from the PubChem database and used as input for the in silico analyses.

Prediction of biological activity spectra was performed using the PASS online server (Prediction of Activity Spectra for Substances; (http://www.way2drug.com/passonline, accessed on 10 April 2026), which estimates the probabilities of activity (Pa) and inactivity (Pi) based on structure–activity relationships derived from biologically active compounds. Activities with *Pa* > *Pi* were retained for interpretation [31,32].

Theoretical pharmacokinetic properties, drug-likeness parameters, predicted skin permeability, and structural alerts were estimated using the SwissADME platform (http://www.swissadme.ch, accessed on 10 April 2026). The analysis considered molecular descriptors relevant to topical delivery, including lipophilicity, topological polar surface area, hydrogen bond donors and acceptors, Lipinski’s rule of five, and predicted skin permeability coefficient (logKp) [32].

### 4.4. Experimental Design of Nanoemulsions

The experimental design was performed to optimize the composition and ultrasound processing conditions of oil-in-water nanoemulsions containing *B. suaveolens* (Willd.) Sweet hydroethanolic leaf extract (BSLE). Copaiba oil was used as the oil phase, whereas Pluronic^®^ F-127 was selected as the nonionic stabilizer based on its amphiphilic properties, biocompatibility, and previous application at 10% (*w*/*v*) in ultrasonically prepared nanoemulsions [26]].

During preliminary formulation screening, aqueous Pluronic^®^ F-127 (Sigma-Aldrich, St. Louis, MO, USA) solutions at 10% and 20% (*w*/*v*) were evaluated. Since increasing the concentration to 20% did not improve BSLE solubilization, the 10% solution was retained as the continuous phase to limit the surfactant content while ensuring adequate interfacial stabilization during high-energy emulsification. The 10% (*w*/*v*) value refers to the concentration of Pluronic^®^ F-127 in the aqueous phase used throughout the experimental design. In the optimized formulation, this phase represented 70% (*v*/*v*), corresponding to a nominal final Pluronic^®^ F-127 concentration of 7% (*w*/*v*). Two sequential experimental steps were conducted: first, optimization of the oil phase/aqueous phase ratio; second, optimization of the ultrasound processing parameters.

In the first step, the proportions of copaiba oil phase and aqueous phase were varied to define the most suitable oil-in-water balance for nanoemulsion formation. The experimental matrix used for the oil phase/aqueous phase optimization is shown in Table 2.

In the second step, ultrasound processing was optimized by varying sonication time and amplitude, while maintaining the ultrasonic cycle fixed at 1. The experimental matrix used for processing optimization is shown in Table 3.

At a fixed total BSLE loading of 20 mg mL^−1^, mean droplet diameter and polydispersity index (PDI) were used as response variables for model fitting and formulation selection. Statistical analysis was performed using Statistica 7 software (StatSoft, Tulsa, OK, USA), considering *p* < 0.05. The optimized conditions were subsequently applied to prepare BSLE-NE and the corresponding copaiba oil nanoemulsion, as described in Section 4.5. Instrument-generated DLS reports for the formulation-composition optimization, ultrasound-processing optimization, and final optimized BSLE-NE are provided in Supplementary Data S1 and S2.

### 4.5. Development of Nanoemulsions

BSLE-NE and Copaiba-NE were prepared by a high-energy emulsification method using probe ultrasonication (UP100H, Hielscher Ultrasonics, Berlin, Germany). BSLE-NE was defined as the nanoemulsion containing *B. suaveolens* (Willd.) Sweet hydroethanolic leaf extract (BSLE), whereas Copaiba-NE was prepared without BSLE and used as the nanoemulsion control in the in vivo wound healing assay. The composition of each formulation is described in Table 4.

For BSLE-NE preparation, the BSLE was incorporated into copaiba oil under magnetic stirring until visual homogeneity. For Copaiba-NE, the same procedure was performed without addition of BSLE. The aqueous phase containing 10% (*w*/*v*) of Pluronic^®^ F-127 was then gradually added under continuous stirring to obtain a coarse oil-in-water pre-emulsion.

BSLE was incorporated as the plant-derived active phytocomplex. BSLE was introduced through the copaiba oil phase, which was selected during preformulation screening as the incorporation medium before formation of the oil-in-water pre-emulsion. As BSLE is a multicomponent hydroethanolic extract, the distribution of its individual constituents between the dispersed oil phase, the oil–water interface, and the aqueous continuous phase may vary according to their physicochemical properties. The aqueous Pluronic^®^ F-127 solution constituted the continuous phase, with poloxamer 407 acting as the nonionic interfacial stabilizer: its hydrophobic poly(propylene oxide) (PPO) blocks associate with the oil phase, whereas the hydrated poly(ethylene oxide) (PEO) chains extend into the aqueous medium, providing steric stabilization against droplet aggregation and coalescence. The 10% (*w*/*v*) concentration provided adequate interfacial stabilization while avoiding an unnecessary increase in surfactant content.

The pre-emulsions were processed using a UP100H probe-type ultrasonic processor (Hielscher Ultrasonics, Berlin, Germany; 100 W, 30 kHz) at cycle 1 and 70% amplitude for 3 min while maintained in an ice bath at approximately 5 °C. The selected process generated oil-in-water nanoemulsions with copaiba oil as the dispersed lipid phase and Pluronic^®^ F-127 aqueous solution as the continuous phase. BSLE-NE was used as the main formulation for physicochemical characterization, in vitro assays, and in vivo wound healing evaluation. Copaiba-NE was used as the nanoemulsion control in the in vivo assay [26,33].

### 4.6. Characterization of Nanoemulsions

Nanoemulsions were characterized in terms of mean hydrodynamic droplet diameter and polydispersity index (PDI) by dynamic light scattering (DLS) using a Zetasizer Nano ZS (Malvern Instruments, Worcestershire, UK). Samples were diluted in distilled water (1:20, *v*/*v*) and analyzed at 25 °C in disposable sizing cuvettes using backscatter detection at 173°, with the measurement position set at 4.65 mm and attenuator setting 8.

Three consecutive records were acquired for each sample. Results were reported as Z-average hydrodynamic droplet diameter, polydispersity index (PDI), and intensity-weighted size distribution, with Z-average and PDI values expressed as mean ± standard deviation (SD) (n = 3). Measurements were carried out immediately after preparation and after 1, 7, 14, and 28 days of storage to evaluate the colloidal stability of the formulations [26,34].

The pH of the nanoemulsions was measured using a calibrated digital pH meter with standard buffer solutions (pH 4.0 and 7.0). Samples were diluted to 10% (*v*/*v*) in distilled water, and all measurements were performed in independent triplicate.

### 4.7. Preliminary Short-Term Physicochemical Stability Assessment

The physicochemical behavior of BSLE-NE was monitored for 28 days as a preliminary short-term assessment conducted during formulation development to identify early changes under different storage temperatures. This assessment was not designed as a formal accelerated stability study and was not used to establish or predict the shelf life of the formulation. Freshly prepared samples were stored in hermetically sealed amber glass vials under three controlled thermal conditions: a temperature-controlled laboratory environment (25 ± 0.5 °C), refrigeration (8 ± 0.5 °C), and a temperature-controlled incubator (40 ± 0.5 °C). Because the primary containers were hermetically sealed, relative humidity was not included as an independent stress variable in this preliminary assessment.

Analyses were performed after 1, 7, 14, and 28 days of storage. Macroscopic parameters, including color, odor, brightness, precipitate formation, visible creaming, and phase separation, were assessed by visual inspection. Mean droplet diameter, polydispersity index (PDI), and pH were determined as described in Section 4.6. All analyses were performed in independent triplicate [26].

### 4.8. Transmission Electron Microscopy

The morphology of BSLE-NE was evaluated by transmission electron microscopy (TEM) using a HITACHI HT7800 microscope (Hitachi High-Tech Corporation, Tokyo, Japan) operated at 100 kV. The formulation was diluted in distilled water (1:1, *v*/*v*), and 10 μL of the diluted dispersion was deposited onto copper grids coated with a formvar film.

Excess liquid was carefully removed using filter paper, and the grids were air-dried at room temperature. Negative staining was performed using uranyl acetate solution to enhance the visualization of the dispersed droplets. The micrographs were used to assess droplet morphology, size distribution, and structural organization of the BSLE-NE system [26].

### 4.9. DPPH Radical-Scavenging Activity of BSLE

The 2,2-diphenyl-1-picrylhydrazyl (DPPH; Sigma-Aldrich, St. Louis, MO, USA) assay was selected as an initial concentration–response screening method to evaluate the radical-scavenging capacity of non-formulated BSLE in 96-well microplates. BSLE-NE and the formulation components were not included in this assay. BSLE was dissolved in methanol and diluted to concentrations ranging from 5 to 500 μg mL^−1^. For each well, 100 μL of the BSLE solution and 100 μL of methanolic DPPH solution (0.1 mM) were added.

After incubation for 30 min in the dark, absorbance was measured at 515 nm using a SpectraMax 340^®^ (San Jose, CA, USA) microplate reader (Molecular Devices). Quercetin was used as a positive control.

The antioxidant activity (AA) was calculated using the following equation:

%AA = [(Abs_control − Abs_sample)/Abs_control] × 100

where Abs_control corresponds to the absorbance of the DPPH solution and Abs_sample to the absorbance of the mixture containing the extract. All experiments were performed in independent triplicate (n = 3). The half-maximal effective concentration (EC_50_) values were determined by regression analysis of the concentration–antioxidant activity curves [35].

### 4.10. Antimicrobial Activity

The antimicrobial activity of *B. suaveolens* (Willd.) Sweet hydroethanolic leaf extract (BSLE) and the BSLE-loaded nanoemulsion (BSLE-NE) was evaluated using the agar disc-diffusion method adapted from the susceptibility assay described by Bauer et al. [36]. To allow direct comparison between the free and nanoemulsified extract, BSLE and BSLE-NE were tested at an equivalent BSLE concentration of 250 μg mL^−1^. BSLE-NE was diluted from the optimized formulation containing 20 mg mL^−1^ BSLE to obtain the same extract-equivalent concentration used for free BSLE.

The microorganisms evaluated were *Staphylococcus aureus*, *Escherichia coli* CCBH 04631, and *Pseudomonas aeruginosa* CCBH 04635, obtained from the Coleção de Culturas de Bactérias de Origem Hospitalar at Fundação Oswaldo Cruz (CCBH/Fiocruz, Rio de Janeiro, Brazil); clinical isolates of *Klebsiella pneumoniae* and *Proteus mirabilis*, with *K. pneumoniae* originating from a urinary tract infection; *Candida albicans*, obtained from the Microbiology Laboratory of Centro Universitário Serra dos Órgãos (UNIFESO, Teresópolis, Brazil); and *Yarrowia lipolytica* IMUFRJ 50682, obtained from the BIOSE Laboratory at the Federal University of Rio de Janeiro (UFRJ, Rio de Janeiro, Brazil). Microbial suspensions were adjusted to a 0.5 McFarland standard, corresponding to approximately 1 × 10^8^ colony-forming units (CFU) mL^−1^, and inoculum density was standardized before plating. The suspensions were inoculated onto Mueller–Hinton agar plates (Reagen, Colombo, Brazil) using sterile swabs.

Sterile paper discs (6 mm) were impregnated with 10 μL of BSLE solution prepared in 70% (*v*/*v*) ethanol or 10 μL of BSLE-NE diluted in sterile distilled water, corresponding to 2.5 μg of BSLE per disc in both groups. To account for the contribution of the formulation matrix, Copaiba-NE, which had the same copaiba oil and Pluronic^®^ F-127 composition and was prepared under the same processing conditions as BSLE-NE but without BSLE, was diluted in sterile distilled water and evaluated under equivalent conditions as the matched formulation control. The same 70% (*v*/*v*) aqueous ethanol used to prepare the free BSLE solution was applied at 10 μL per disc as its matched vehicle control, and the following standardized antimicrobial discs were used as positive controls according to the microorganism tested: ampicillin (10 µg), trimethoprim-sulfamethoxazole (25 µg), tetracycline (30 µg), gentamicin (10 µg), ciprofloxacin (5 µg), levofloxacin (5 µg), cephalothin (30 µg), and imipenem (10 µg) (Laborclin, Pinhais, PR, Brazil).

Plates were incubated at 37 °C for 24 h for bacterial strains and at 30 °C for 48 h for yeasts. After incubation, inhibition-zone diameters were measured, and antimicrobial activity was expressed as a control-corrected relative inhibition index (%I), calculated according to the following equation:

%I = [(H_sample − H_control)/H_sample] × 100

where H_sample is the mean inhibition-zone diameter obtained with BSLE or BSLE-NE and H_control is the mean inhibition-zone diameter obtained with the corresponding control condition (70% ethanol for BSLE and Copaiba-NE diluted in sterile distilled water for BSLE-NE). Thus, %I represents the relative inhibition associated with each sample after correction for its matched control. All assays were performed in independent triplicate (n = 3).

### 4.11. Cytotoxicity Assay

Cytotoxicity was evaluated using the resazurin (Sigma-Aldrich, St. Louis, MO, USA) reduction assay in J774 macrophages obtained from the National Institute of Metrology, Quality and Technology (INMETRO-RJ, Xerén, Brazil), HaCaT keratinocytes obtained from the Bioinovar Laboratory at the Institute of Microbiology Paulo de Góes, Federal University of Rio de Janeiro (IMPG-UFRJ, Rio de Janeiro, Brazil), and human fibroblasts obtained from the American Type Culture Collection (HFB; ATCC^®^ PCS-201-012™). The tested samples were *B. suaveolens* (Willd.) Sweet hydroethanolic leaf extract (BSLE), the BSLE-loaded nanoemulsion (BSLE-NE), Pluronic^®^ F-127 aqueous solution at 10%, and copaiba oil. Pluronic^®^ F-127 and copaiba oil were evaluated as formulation-component controls to assess their individual influence on cell viability.

Cells were cultured in Dulbecco’s Modified Eagle Medium (DMEM; Sigma-Aldrich, St. Louis, MO, USA) supplemented with 10% fetal bovine serum (FBS; Sigma-Aldrich, St. Louis, MO, USA) and maintained at 37 °C in a humidified atmosphere containing 5% CO_2_. Cell suspensions (1 × 10^6^ cells mL^−1^) were seeded into 96-well microplates and incubated for 6 h to allow cell adhesion. Subsequently, cells were exposed for 48 h to free BSLE or BSLE-NE at matched extract concentrations of 3.90, 7.81, 15.62, 31.25, 62.5, 125, 250, and 500 μg mL^−1^. For BSLE-NE, these concentrations were calculated from the BSLE content of the optimized formulation (20 mg mL^−1^) and expressed as BSLE-equivalent concentrations, ensuring that each nanoemulsion dilution contained the same nominal BSLE concentration as the corresponding free-extract solution. Pluronic^®^ F-127 and copaiba oil were evaluated under the corresponding formulation-component conditions.

After treatment, 20 μL of resazurin solution (5 mg mL^−1^) was added to each well, and plates were incubated for 4 h. Absorbance was measured at 490 and 595 nm using a SpectraMax M5^®^ microplate reader (Molecular Devices, USA). Background absorbance was subtracted before calculations. Cell viability was calculated according to the following equation:

% Viability = [(OD_sample − OD_blank)/(OD_control − OD_blank)] × 100

where OD_sample corresponds to the absorbance of treated cells, OD_blank corresponds to the absorbance of wells without cells, and OD_control corresponds to untreated cells. Concentrations causing 50% and 90% reductions in cell viability (CC_50_ and CC_90_, respectively) were estimated by nonlinear regression analysis when concentration-response curves were obtained. All assays were performed in independent triplicate (n = 3) [37].

### 4.12. In Vivo Wound Healing Assay and Wound Contraction Analysis

The wound healing activity of BSLE-NE was evaluated in an excisional wound model using 15 female *Wistar* rats (*Rattus norvegicus albinus*), aged three months and weighing 300 ± 20 g, obtained from the animal facility of Centro Universitário Serra dos Órgãos (UNIFESO, Teresópolis, Brazil). Animals were maintained under controlled temperature conditions (24 ± 2 °C), a 12 h light/dark cycle, and had free access to food and water. Animals were monitored daily for signs of distress. All experimental procedures were approved by the Ethics Committee on Animal Use (CEUA-UNIFESO; protocol n^o^. 569/2024).

Following intraperitoneal anesthesia with ketamine (100 mg kg^−1^) and xylazine (10 mg kg^−1^), both supplied by Syntec do Brasil Ltd.a. (Santana de Parnaíba, SP, Brazil), the dorsal region was shaved and disinfected. Circular excisional wounds were created using a sterile dermatological punch with an 8 mm diameter. Animals were randomly allocated into three experimental groups (n = 5 per group): untreated control, Copaiba-NE, and BSLE-NE. BSLE-NE was evaluated as the test formulation containing *B. suaveolens* (Willd.) Sweet hydroethanolic leaf extract, whereas Copaiba-NE, prepared with the same copaiba oil/Pluronic^®^ F-127 matrix but without BSLE, served as the matched formulation control. This design allowed the biological response associated with the BSLE-containing formulation to be assessed relative to the corresponding nanoemulsion vehicle. A volume of 20 μL of the respective nanoemulsion (BSLE-NE or Copaiba-NE) was applied topically to each wound once daily until the end of the experimental period.

Wound healing progression was assessed by macroscopic and morphometric analysis of the lesions on days 1, 4, 8, and 15 after wound induction. Wounds were photographed using a Basike Ba-CAM215 digital camera (Basike, Begin, China) positioned at a fixed distance, with a millimeter scale included in each image for calibration. Wound diameter was measured in millimeters using ImageJ (version 1.54p)^®^ software (National Institutes of Health, Bethesda, MD, USA).

The wound contraction rate was calculated according to the following equation:

Wound contraction (%) = [(D_i_ − D_t_)/D_i_] × 100

where D_i_ represents the initial wound diameter measured after wound induction, and D_t_ represents the wound diameter measured at each experimental time point. Measurements were performed by two independent blinded evaluators, and results were expressed as mean ± standard deviation.

### 4.13. Histological Evaluation of Wound Healing

At the end of the experimental period, tissue samples from the wound area were collected and fixed in 10% buffered formaldehyde for 24 h. Samples were dehydrated through a graded ethanol series, cleared in xylene, and embedded in paraffin.

Histological sections (4 μm) were obtained using a rotary microtome (Leica Biosystems^®^, São Paulo, Brazil) and stained with hematoxylin and eosin (H&E). Slides were examined under a light microscope (Nikon E-200, Shinagawa, Japan) at magnifications of 4×, 10×, and 40×. Image acquisition and measurements were performed using ImageJ software (National Institutes of Health, Bestheda, MD, USA).

Histopathological evaluation was conducted by a blinded examiner using the murine cutaneous wound scoring system described by Vyver et al. [38], which includes assessment of re-epithelialization, epithelial thickness, keratinization, granulation tissue, scar elevation index, and tissue remodeling. The total score ranged from 0 to 12, indicating the stage of wound healing. Analyses were performed under standardized conditions.

### 4.14. Biochemical and Hematological Analysis

Biochemical and hematological evaluations were performed at the end of the experimental period, on day 15, to investigate potential systemic effects associated with topical treatment with the nanoemulsion formulations. The evaluated groups were Copaiba-NE and BSLE-NE.

Blood samples were collected by intracardiac puncture under deep anesthesia using sterile syringes. For hematological analysis, blood was collected into tubes containing 3.8% sodium citrate at a ratio of 1:9 (anticoagulant:blood). For biochemical analysis, blood samples were collected without anticoagulant and processed immediately after collection by centrifugation at 4000 rpm for 15 min to obtain serum.

Serum biochemical analysis included alanine aminotransferase (ALT) and aspartate aminotransferase (AST) as hepatic biochemical markers, together with creatinine and urea as renal biochemical markers. ALT and AST activities were determined using ultraviolet kinetic methods based on spectrophotometric monitoring of reduced nicotinamide adenine dinucleotide (NADH) oxidation. Creatinine was determined by a kinetic method, and urea was determined by an enzymatic ultraviolet method. All determinations were performed using veterinary clinical chemistry kits (Bioclin Vet; Quibasa Química Básica Ltd.a., Belo Horizonte, MG, Brazil) according to the manufacturer’s instructions, with readings obtained using an Aud-BQ VET veterinary biochemical analyzer (Labtest Diagnóstica S.A., Lagoa Santa, MG, Brazil).

Hematological evaluation was performed by complete blood count, including erythrocyte count, hemoglobin, hematocrit, erythrocyte indices, total leukocyte count, differential leukocyte count, and platelet count. Results were expressed in standardized units for each parameter and compared with physiological reference ranges reported for *Rattus norvegicus* under experimental conditions [39].

### 4.15. Statistical Analysis

Statistical analysis was performed using GraphPad Prism software version 8.0.1 (GraphPad Software, San Diego, CA, USA). Results were expressed as mean ± standard deviation (SD). Differences were considered statistically significant at *p* < 0.05, with a 95% confidence interval.

Antimicrobial activity data were analyzed by ordinary two-way ANOVA, considering formulation and microorganism as independent factors, followed by Šídák’s multiple comparisons test. Other comparisons among independent experimental groups were performed using ordinary one-way ANOVA followed by Tukey’s post hoc test, when applicable. In vitro experiments were performed in independent triplicate (n = 3).

## 5. Conclusions

Qualitative UHPLC–ESI–QTOF–MS analysis of the *Brugmansia suaveolens* (Willd.) Sweet hydroethanolic leaf extract (BSLE) detected 39 chromatographic features, including three putatively annotated tropane alkaloids scopolamine, 3-hydroxy-6-tigloyloxytropane, and noratropine. Copaiba oil exhibited a predominantly sesquiterpenic composition, with β-caryophyllene as its principal constituent. The optimized formulation, BSLE-NE, was a Pluronic^®^ F-127-stabilized oil-in-water nanoemulsion characterized by nanoscale droplets, low polydispersity, predominantly spherical morphology, and preliminary short-term physicochemical stability.

Nanoemulsification preserved the antimicrobial activity of BSLE against the most susceptible bacterial strains, particularly *Klebsiella pneumoniae* and *Proteus mirabilis*, while improving cytocompatibility in J774 macrophages and maintaining high viability in human fibroblasts and HaCaT keratinocytes. In the experimental wound-healing model, BSLE-NE significantly reduced residual wound diameter and improved histological tissue organization compared with both the untreated control and the copaiba oil nanoemulsion without BSLE.

Collectively, these findings support BSLE-NE as a promising dual-component topical formulation integrating *B. suaveolens* leaf extract and bioactive copaiba oil for cutaneous wound repair. However, the absence of a dose-matched non-formulated BSLE control precludes definitive conclusions regarding the specific contribution of nanoemulsification to the observed therapeutic response. Furthermore, the reduced leukocyte, neutrophil, and lymphocyte counts observed in both treated groups warrant additional investigation. Future studies should include extended physicochemical stability testing, comparisons with non-formulated BSLE, cutaneous permeation and tissue-retention analyses, and comprehensive long-term toxicological and hematological assessments to clarify the formulation’s safety, delivery characteristics, and translational potential.

## Figures and Tables

**Figure 1:**
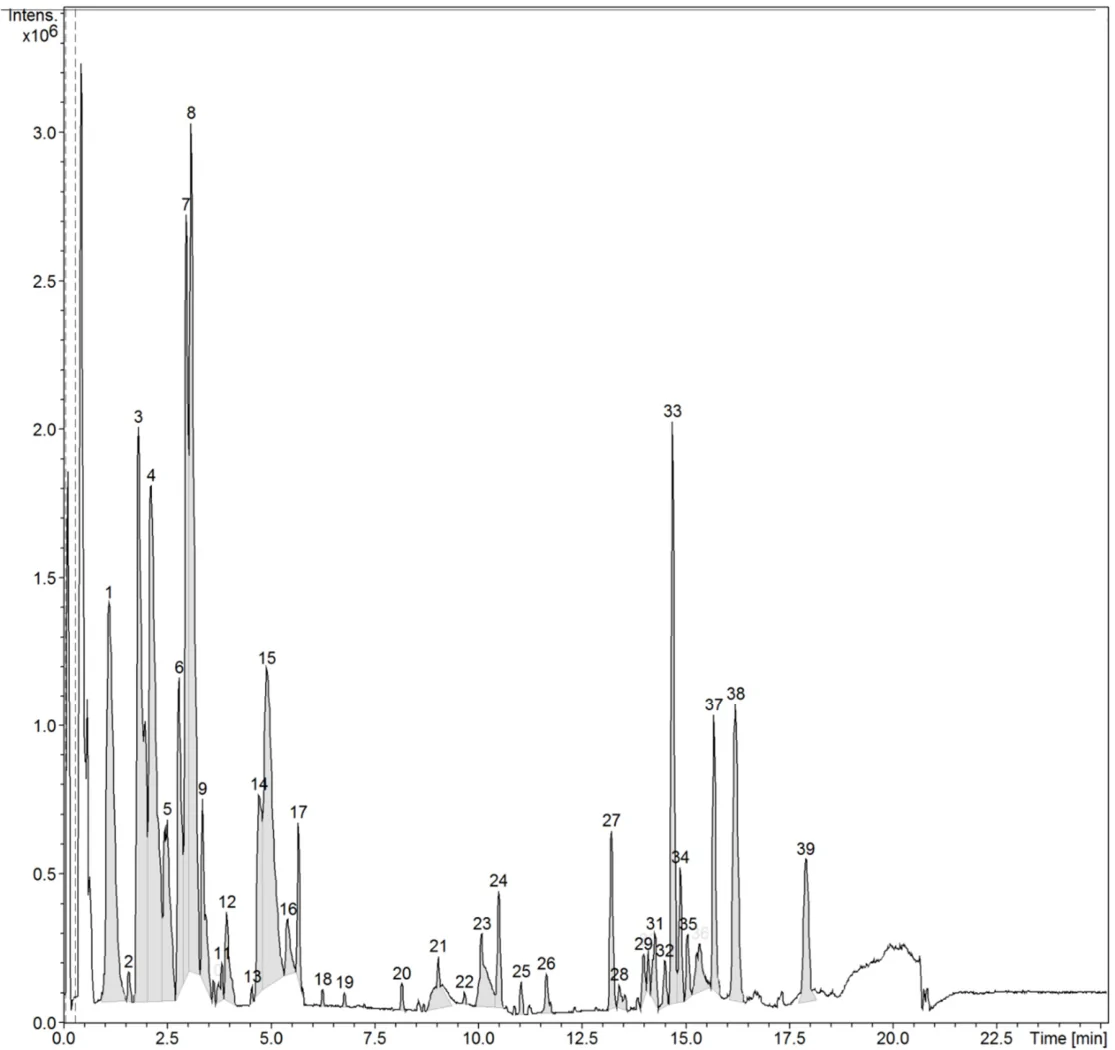
UHPLC–ESI–QTOF–MS base peak chromatogram of *B. suaveolens* hydroethanolic leaf extract (BSLE) obtained in positive ionization mode. Numbers 1–39 correspond to the detected chromatographic features listed in Table S6. Representative metabolites with supported putative annotations are summarized in Table 1 of the main manuscript. BSLE, *B. suaveolens* (Willd.) Sweet hydroethanolic leaf extract.

**Figure 2:**
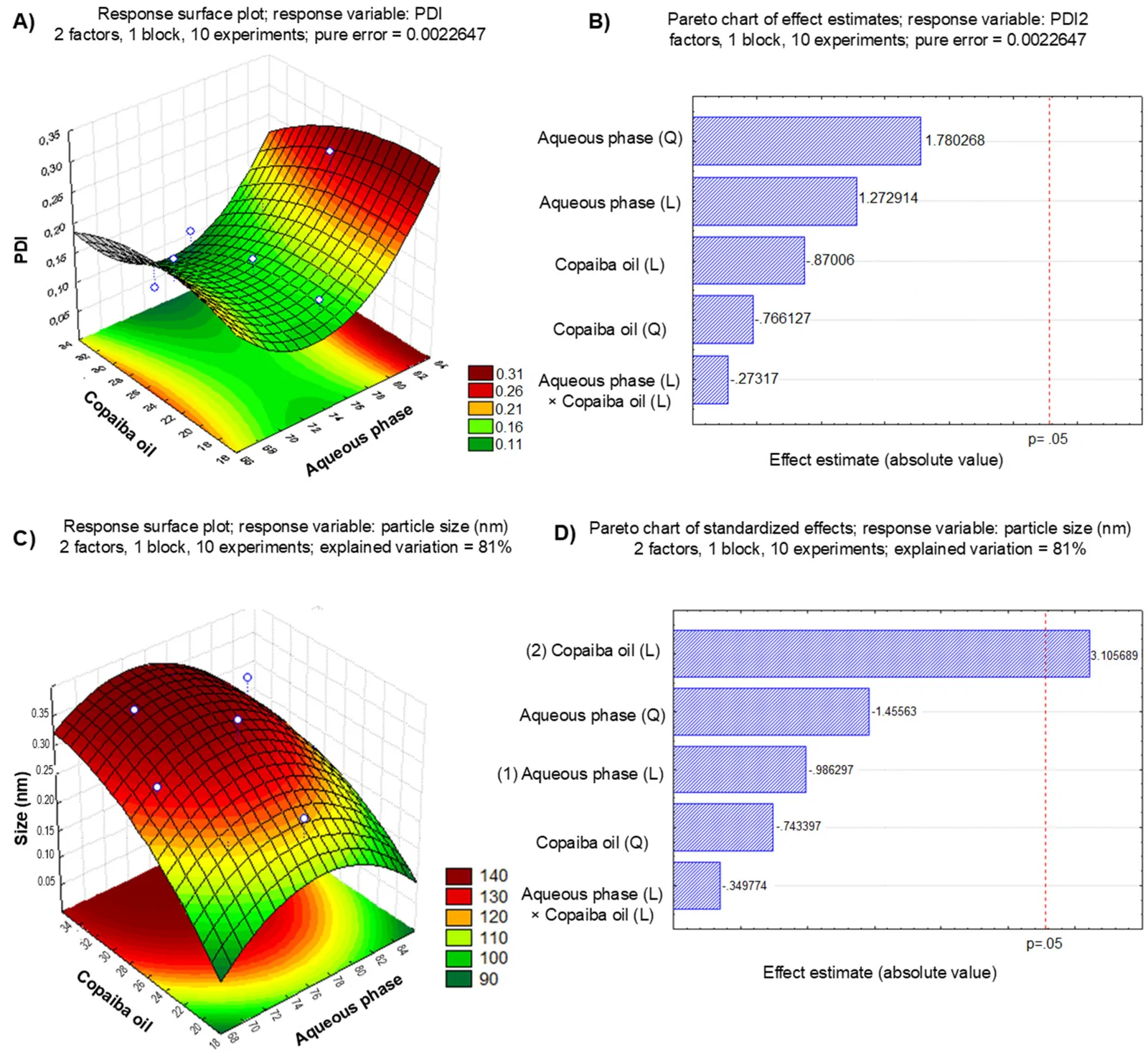

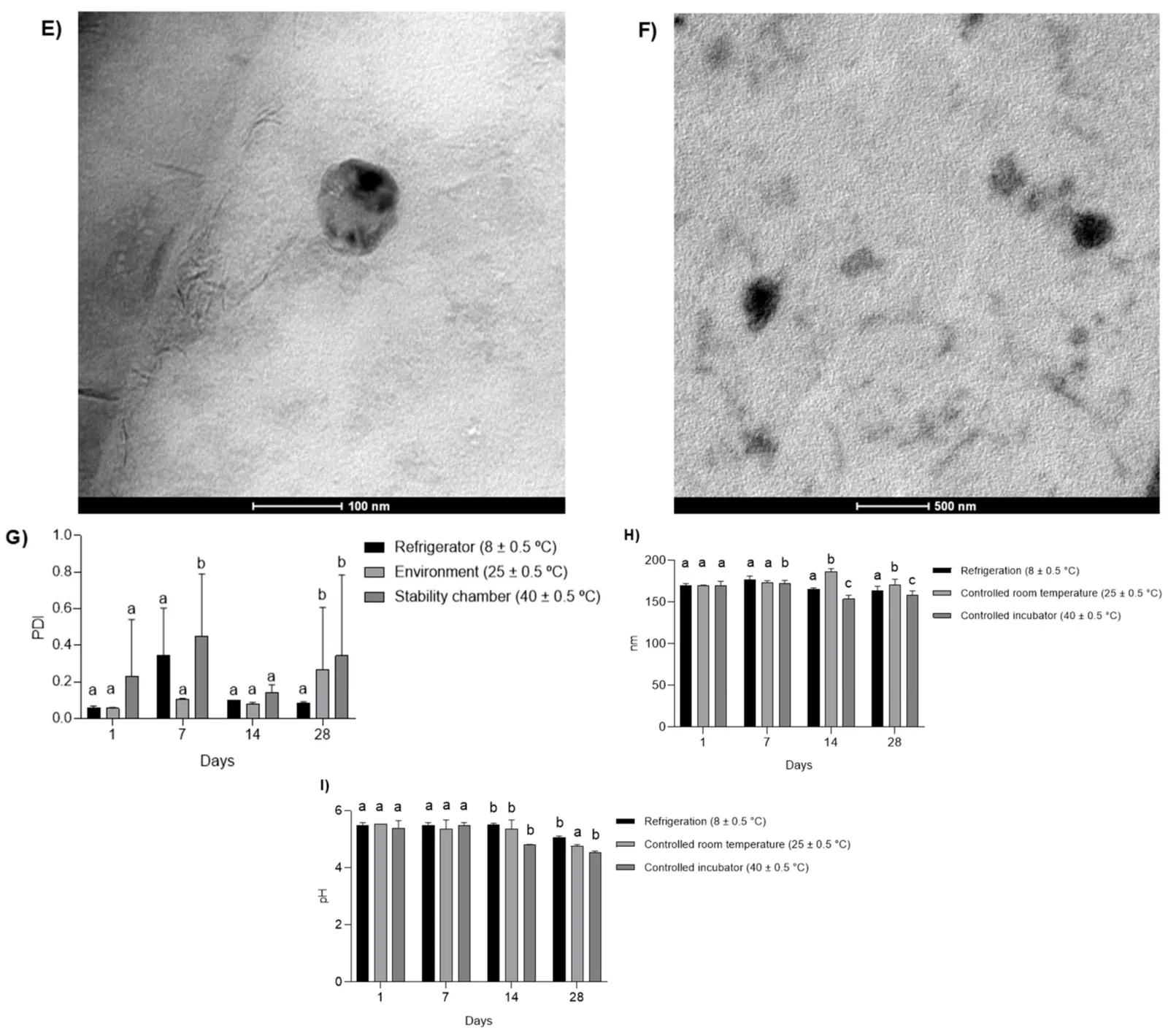
Physicochemical optimization, morphology, and stability of BSLE-NE. (**A**,**B**) Response surface plot and Pareto chart for PDI. (**C**,**D**) Response surface plot and Pareto chart for mean droplet diameter. (**E**,**F**) TEM images of BSLE-NE droplets: E shows a representative TEM visualization at higher magnification, with a 100 nm scale bar; F shows a panoramic TEM field at lower magnification, with a 500 nm scale bar. (**G**–**I**) Stability profile of BSLE-NE over 28 days under different storage conditions—refrigerated storage (8 ± 0.5 °C), a temperature-controlled laboratory environment (25 ± 0.5 °C), and storage in a temperature-controlled incubator (40 ± 0.5 °C): (**G**) shows the variation in PDI; (**H**) shows the mean droplet diameter; and (**I**) shows the pH values at days 1, 7, 14, and 28. Data are expressed as mean ± standard deviation (SD) (n = 3). At each time point, different lowercase letters indicate significant differences among storage conditions (*p* < 0.05). BSLE-NE, nanoemulsion containing *B. suaveolens* hydroethanolic leaf extract; PDI, polydispersity index; TEM, transmission electron microscopy.

**Figure 3:**
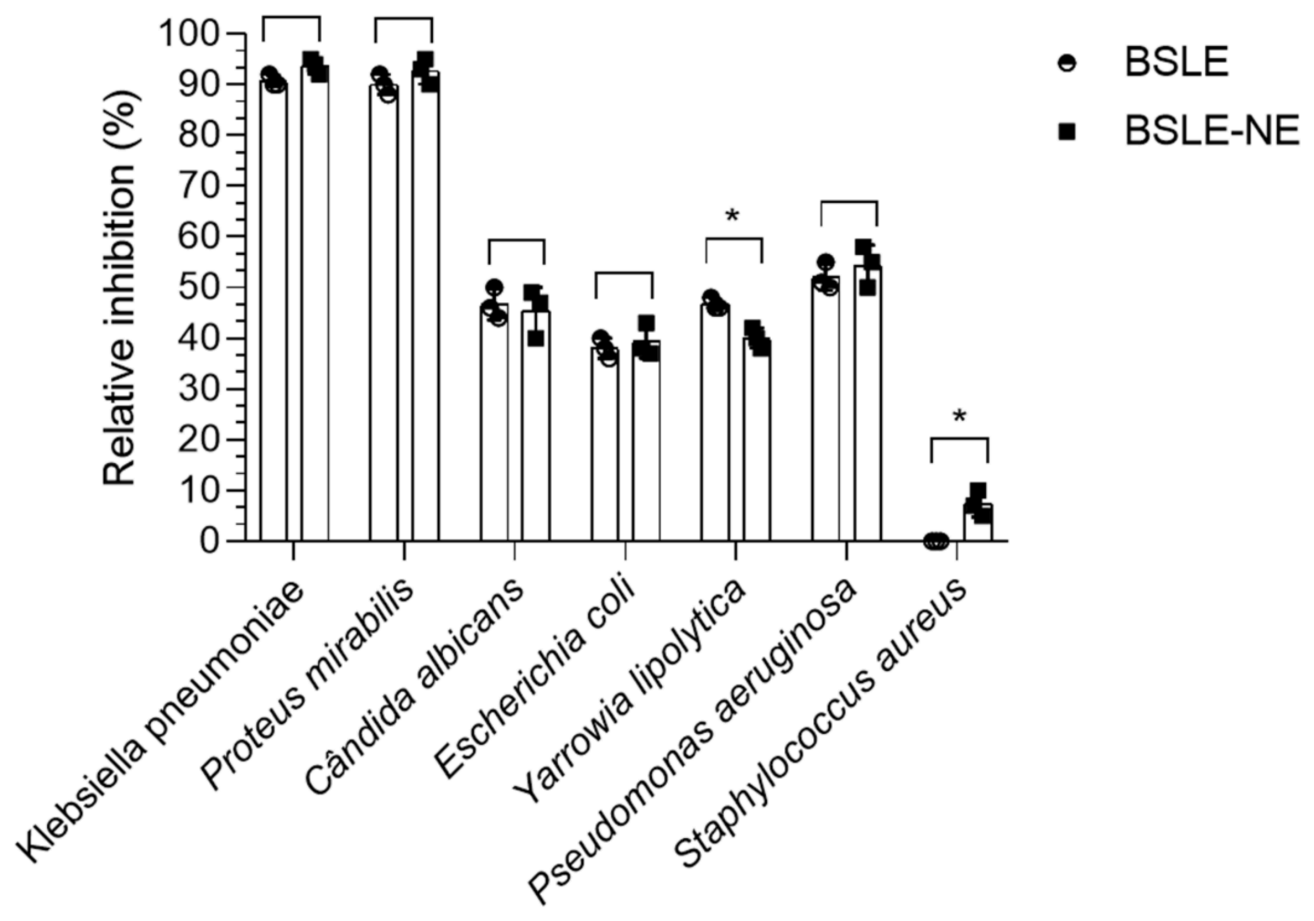
Control-corrected relative antimicrobial inhibition of BSLE and BSLE-NE against selected microorganisms. Antimicrobial activity of *B. suaveolens* hydroethanolic leaf extract (BSLE) and BSLE-loaded nanoemulsion (BSLE-NE) against *Klebsiella pneumoniae*, *Proteus mirabilis*, *Candida albicans*, *Escherichia coli*, *Yarrowia lipolytica*, *Pseudomonas aeruginosa*, and *Staphylococcus aureus*. Values represent relative inhibition based on inhibition-zone diameters after correction for the corresponding control. Data are expressed as mean ± SD (n = 3). Statistical analysis was performed using ordinary two-way ANOVA followed by Šídák’s multiple comparisons test comparing BSLE and BSLE-NE within each microorganism. * *p* < 0.05. BSLE, *B. suaveolens* (Willd.) Sweet hydroethanolic leaf extract; BSLE-NE, nanoemulsion containing BSLE.

**Figure 4:**
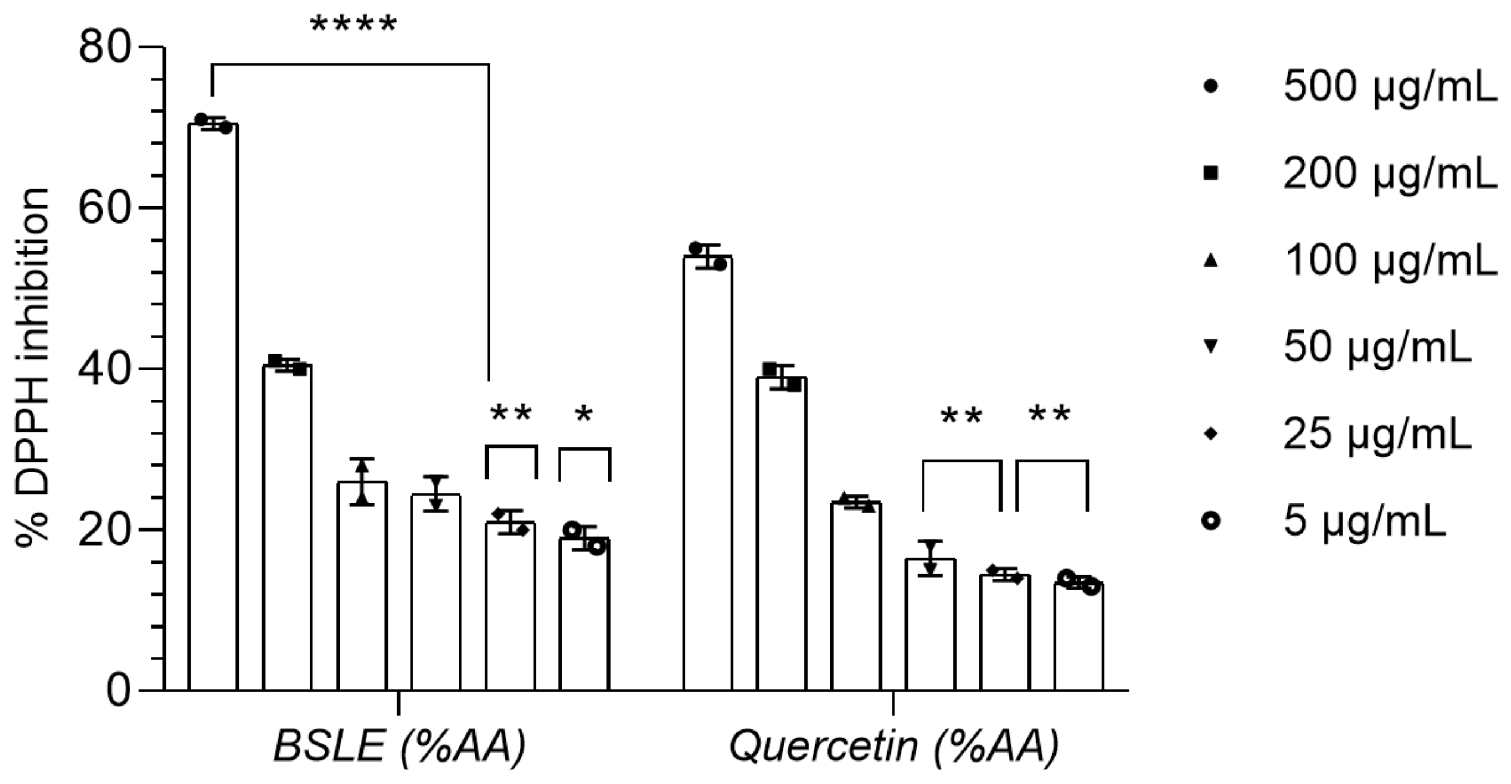
Antioxidant activity of BSLE compared with quercetin. DPPH radical scavenging activity of *B. suaveolens* hydroethanolic leaf extract (BSLE) and quercetin at concentrations ranging from 5 to 500 µg mL^−1^. Data are expressed as mean ± SD. Statistical analysis was performed using ordinary two-way ANOVA followed by Šídák’s multiple comparisons test comparing BSLE and quercetin within each concentration. * *p* < 0.05, ** *p* < 0.01, **** *p* < 0.0001. BSLE, *B. suaveolens* (Willd.) Sweet hydroethanolic leaf extract; %AA, antioxidant activity.

**Figure 5:**
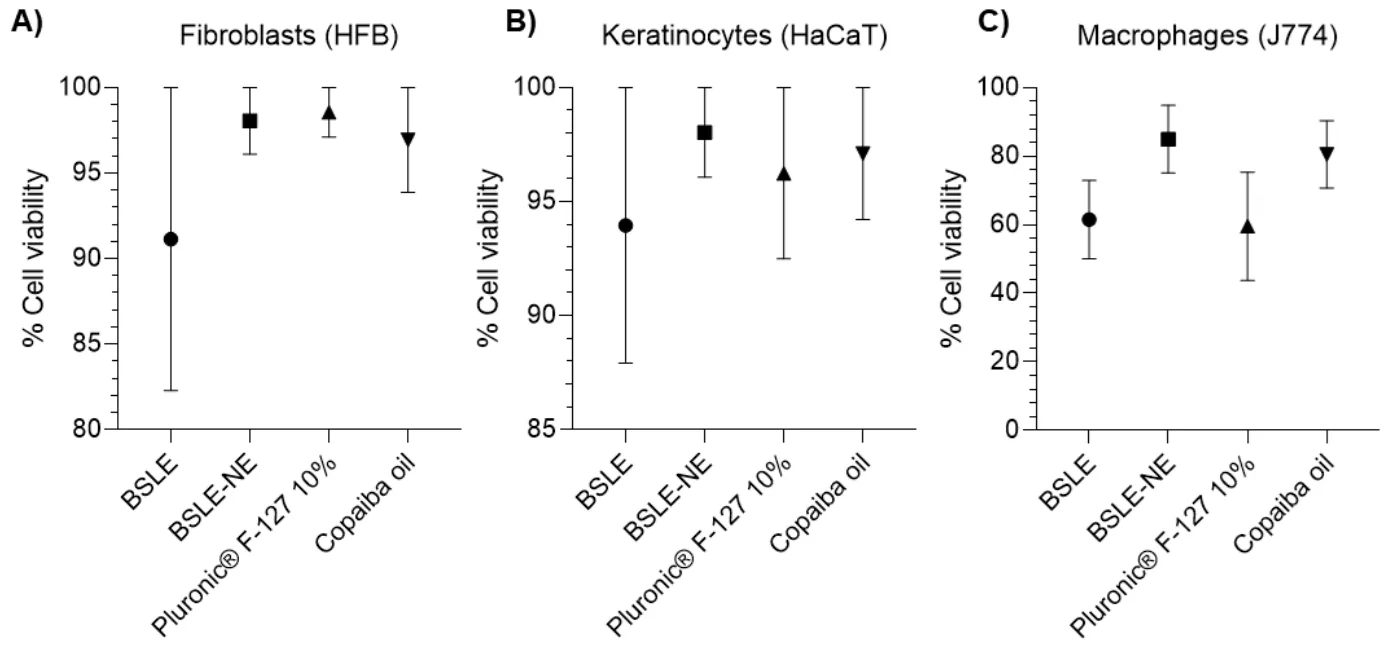
Cell viability of HFB, HaCaT and J774 cells after exposure to BSLE and formulation components. (**A**) Human fibroblasts (HFB), (**B**) HaCaT keratinocytes, and (**C**) J774 macrophages after exposure to *B. suaveolens* hydroethanolic leaf extract (BSLE), BSLE-loaded nanoemulsion (BSLE-NE), Pluronic^®^ F-127 at 10%, and copaiba oil, evaluated at 500 μg mL^−1^. Data are expressed as mean ± SD. The dashed line indicates the 70% cell viability threshold used for cytotoxicity interpretation. BSLE, *B. suaveolens* hydroethanolic leaf extract; BSLE-NE, nanoemulsion containing BSLE; HFB, human fibroblasts.

**Figure 6:**
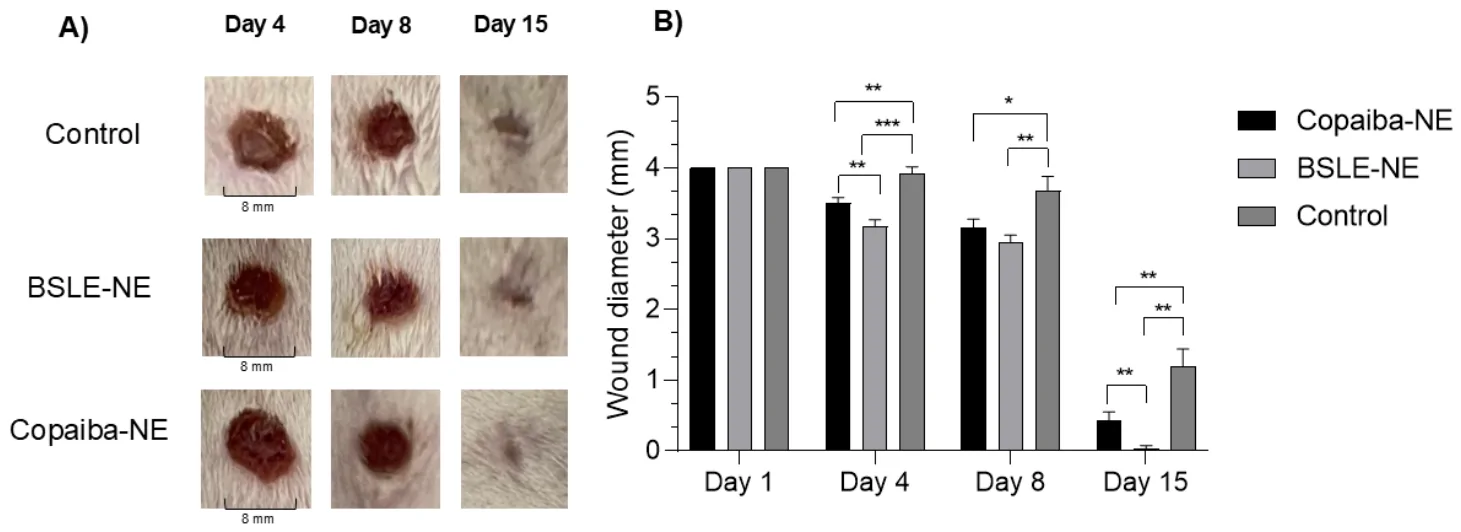
Effect of nanoemulsion treatments on in vivo wound healing. (**A**) Representative macroscopic images of wounds from untreated control, BSLE-NE, and Copaiba-NE groups at days 4, 8, and 15 after wound induction. (**B**) Wound diameter over time in untreated control, Copaiba-NE, and BSLE-NE groups. Data are expressed as mean ± SD. Statistical analysis was performed using two-way repeated-measures ANOVA followed by Tukey’s multiple comparisons test. * *p* < 0.05, ** *p* < 0.01, *** *p* < 0.001. BSLE-NE, nanoemulsion containing *B. suaveolens* (Willd.) Sweet hydroethanolic leaf extract; Copaiba-NE, copaiba oil nanoemulsion without BSLE.

**Figure 7:**
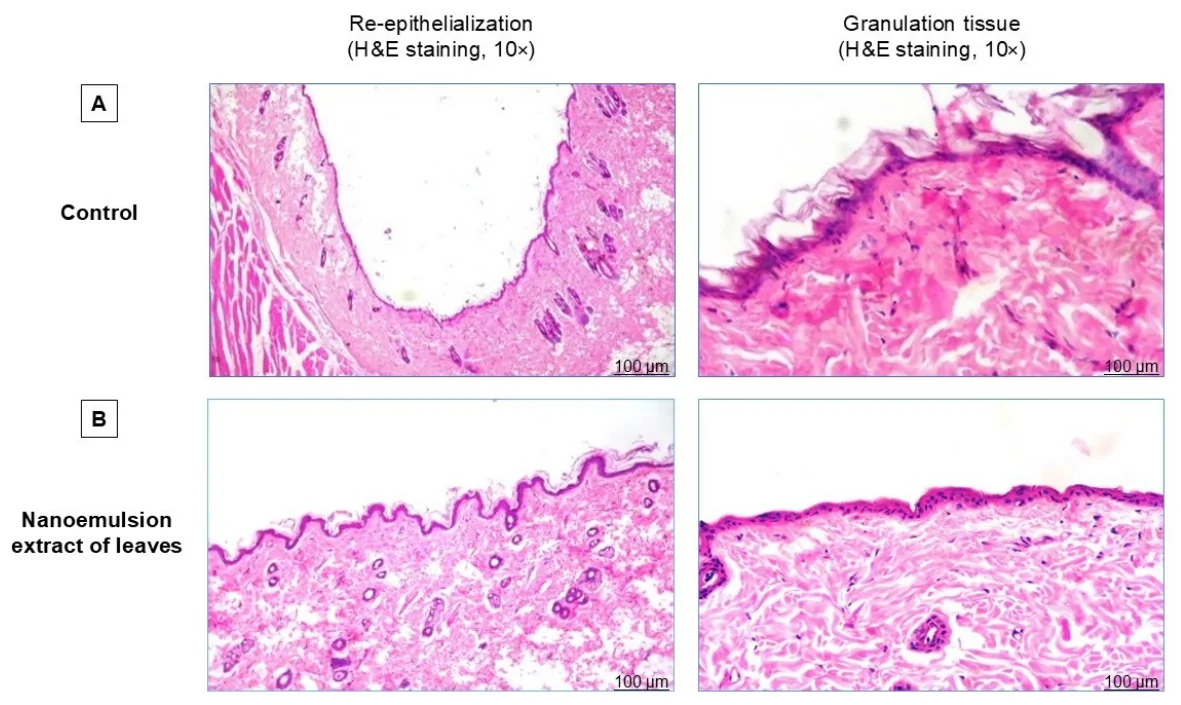
Histopathological features of wound healing after BSLE-NE treatment. Representative hematoxylin and eosin-stained wound sections on day 15 after wound induction. (**A**) Untreated control group, showing re-epithelialization and granulation tissue features. (**B**) BSLE-NE-treated group, showing improved epithelial organization and more structured granulation tissue. The left column shows re-epithelialization, and the right column shows granulation tissue organization. Scale bars: 100 µm. BSLE-NE, nanoemulsion containing *B. suaveolens* (Willd.) Sweet hydroethanolic leaf extract.

**Table 1:** Putative annotation of representative tropane alkaloids detected in *B. suaveolens* (Willd.) Sweet hydroethanolic leaf extract by UHPLC–ESI–QTOF–MS.

Peak	RT (min)	*m*/*z*	Chemical Class	Putative Annotation
4	2.1	304.1567	Tropane alkaloid	Scopolamine
5	2.5	240.1615	Tropane alkaloid	3-Hydroxy-6-tigloyloxytropane
6	2.8	276.1615	Tropane alkaloid	Noratropine

Peak numbers correspond to the chromatographic features presented in Figure 1 and Table S6. Putative annotations were based on observed *m*/*z* values, chromatographic behavior, mass spectral data, and comparisons with literature information. RT, retention time; *m*/*z*, mass-to-charge ratio.

**Table 2:** Experimental design for optimization of the oil phase/aqueous phase ratio in nanoemulsions.

Experiment	Final BSLE Concentration (mg mL^−1^)	Aqueous Phase Containing 10% (*w*/*v*) of Pluronic^®^ F-127 (% *v*/*v*)	Copaiba Oil Phase (% *v*/*v*)
BSLE-NE-01	20	80.0	20.0
BSLE-NE-02	20	78.0	22.0
BSLE-NE-03	20	75.0	25.0
BSLE-NE-04	20	70.0	30.0
BSLE-NE-05	20	82.0	18.0
BSLE-NE-06	20	68.0	32.0
BSLE-NE-07	20	72.0	28.0
BSLE-NE-08	20	77.0	23.0
BSLE-NE-09	20	76.0	24.0
BSLE-NE-10	20	73.0	27.0

BSLE-NE, nanoemulsion containing *B. suaveolens* hydroethanolic leaf extract. The final volume of each formulation was 5 mL. BSLE-NE-04, containing 20 mg mL^−1^ BSLE and 30% (*v*/*v*) copaiba oil, was selected for subsequent studies.

**Table 3:** Experimental design for ultrasound processing optimization of BSLE-NE.

Experiment	Sonication Time (min)	Amplitude (%)
BSLE-NE-01	3	20
BSLE-NE-02	3	70
BSLE-NE-03	9	20
BSLE-NE-04	9	70
BSLE-NE-05	1	50
BSLE-NE-06	9	50
BSLE-NE-07	5	20
BSLE-NE-08	5	85
BSLE-NE-09	5	50
BSLE-NE-10	5	50

BSLE-NE, nanoemulsion containing *B. suaveolens* hydroethanolic leaf extract.

**Table 4:** Composition and identity of the nanoemulsion formulations.

Formulation	Final BSLE Concentration (mg mL^−1^)	Final Copaiba Oil Concentration (% *v*/*v*)	Aqueous Phase (% *v*/*v*)	Final Pluronic^®^ F-127 Concentration (% *w*/*v*)	Processing Condition
BSLE-NE	20	30	70	7	Cycle 1, 70% amplitude, 3 min
Copaiba-NE	0	30	70	7	Cycle 1, 70% amplitude, 3 min

BSLE-NE, nanoemulsion containing *B. suaveolens* hydroethanolic leaf extract; Copaiba-NE, corresponding copaiba oil nanoemulsion without BSLE. The aqueous phase was prepared with Pluronic^®^ F-127 at 10% (*w*/*v*). Because this phase represented 70% (*v*/*v*) of the final formulation, the nominal final Pluronic^®^ F-127 concentration was 7% (*w*/*v*).

## Data Availability

The data presented in this study are available in the article and Supplementary Materials. Additional raw data supporting the findings of this study are available from the corresponding author upon reasonable request.

## Supplementary Materials

The following supporting information can be downloaded at: https://www.mdpi.com/article/10.3390/plants15192901/s1, Table S1: In silico prediction of biological activities of selected representative compounds; Table S2: Predicted physicochemical and pharmacokinetic parameters of selected representative compounds; Table S3: Serum biochemical parameters of treated animals at day 15; Table S4: Hematological parameters of treated animals at day 15; Table S5: Semi-quantitative histopathological scoring of wound healing at day 15; Table S6: UHPLC–ESI–QTOF–MS chromatographic features detected *in B. suaveolens* (Willd.) Sweet hydroethanolic leaf extract; Table S7: Chemical composition of copaiba oil (*Copaifera* sp.) determined by GC–MS; Figure S1: Comprehensive histopathological evaluation of wound healing in different treatment groups; Figure S2: DLS size distribution of the optimized BSLE-NE; Figure S3: Macroscopic appearance of the optimized BSLE-NE; Figure S4: Representative agar disc-diffusion plates showing the antimicrobial activity of BSLE against the seven tested microorganisms; Supplementary Data S1: Instrument-generated DLS reports for the optimization of the oil phase/aqueous phase ratio; Supplementary Data S2: Instrument-generated DLS reports for ultrasound-processing optimization and the final optimized BSLE-NE.

## Abbreviations

The following abbreviations are used in this manuscript:

AA	Antioxidant activity
ALT	Alanine aminotransferase
ANOVA	Analysis of variance
AST	Aspartate aminotransferase
ATCC	American Type Culture Collection
BSLE	*Brugmansia suaveolens* hydroethanolic leaf extract
BSLE-NE	Nanoemulsion containing *Brugmansia suaveolens* hydroethanolic leaf extract
CAPES	Coordenação de Aperfeiçoamento de Pessoal de Nível Superior
CEUA	Ethics Committee on Animal Use
CFU	Colony-forming units
CC_50_	Concentration causing 50% reduction in cell viability
CC_90_	Concentration causing 90% reduction in cell viability
DLS	Dynamic light scattering
DMEM	Dulbecco’s Modified Eagle Medium
DPPH	2,2-Diphenyl-1-picrylhydrazyl
EC_50_	Half-maximal effective concentration
FAPERJ	Fundação Carlos Chagas Filho de Amparo à Pesquisa do Estado do Rio de Janeiro
FBS	Fetal bovine serum
GC–MS	Gas chromatography coupled to mass spectrometry
H&E	Hematoxylin and eosin
HaCaT	Human keratinocyte cell line
HFB	Human fibroblasts
ISO	International Organization for Standardization
J774	Murine macrophage cell line
logKp	Skin permeability coefficient
LogP	Partition coefficient
*m*/*z*	Mass-to-charge ratio
NADH	Reduced nicotinamide adenine dinucleotide
NIST	National Institute of Standards and Technology
Pa	Probability of activity
PARNASO	Serra dos Órgãos National Park
PASS	Prediction of Activity Spectra for Substances
PDI	Polydispersity index
Pi	Probability of inactivity
RI	Retention index
SD	Standard deviation
SISGEN	Brazilian National System for the Management of Genetic Heritage and Associated Traditional Knowledge
TEM	Transmission electron microscopy
TMS	Trimethylsilyl
TPSA	Topological polar surface area
UFRJ	Federal University of Rio de Janeiro
UHPLC–ESI–QTOF–MS	Ultra-high-performance liquid chromatography coupled with electrospray ionization quadrupole time-of-flight mass spectrometry
UHPLC–MS	Ultra-high-performance liquid chromatography coupled with mass spectrometry
UNIFESO	Centro Universitário Serra dos Órgãos
