# Supplementary material for: Dual-Component Nanoemulsion Containing Brugmansia suaveolens Leaf Extract and Copaiba Oil: Antimicrobial Activity and Preclinical Wound-Healing Potential
Source: Plants (Basel). 2026 Sep 23;15(19):2901. doi: 10.3390/plants15192901 (PMC13666830; doi:10.3390/plants15192901)
Supplement: Supplementary file 1 [file plants-15-02901-s001.zip › plants-4468747-supplementary.pdf]

Supplementary material

Supplementary Table

Table S1 *In silico* prediction of biological activities of selected representative compounds

Compound	Predicted activity	Pa	Pi
Scopolamine	Anti-inflammatory	0.742	0.012
Scopolamine	Wound healing stimulant	0.689	0.023
Hyoscyamine (Atropine)	Anti-inflammatory	0.715	0.018
Hyoscyamine (Atropine)	Antibacterial	0.621	0.037
Noratropine	Antimicrobial	0.598	0.044
6-Hydroxyatropine	Anti-inflammatory	0.566	0.052
β-Caryophyllene	Anti-inflammatory	0.854	0.006
β-Caryophyllene	Antimicrobial	0.712	0.021

Biological activities related to wound healing, anti-inflammatory and antimicrobial effects were predicted using the PASS online platform. Activities were considered pharmacologically relevant when the probability of activity (Pa) exceeded the probability of inactivity (Pi).

Abbreviations: Pa, probability of activity; Pi, probability of inactivity.

Table S2. Predicted physicochemical and pharmacokinetic parameters of selected representative compounds.

Compound	MW (g mol^{-1})	*LogP*	TPSA (Å^2)	Skin permeation *logKp* (cm/s)	*Lipinski* violations	Synthetic accessibility
Scopolamine	303.35	1.23	55.8	−5.81	0	4.2
Hyoscyamine	289.37	2.34	49.7	−5.62	0	3.9
Noratropine	275.34	1.87	46.1	−5.74	0	3.7
6-Hydroxyatropine	305.36	1.98	62.5	−5.79	0	4.1
β-Caryophyllene	204.36	4.52	0.0	−4.62	0	2.8

Physicochemical and pharmacokinetic parameters were predicted using the SwissADME platform, including molecular weight (MW), lipophilicity (LogP), topological polar surface area (TPSA), predicted skin permeation coefficient (logKp), Lipinski rule violations and synthetic accessibility.

Abbreviations: MW, molecular weight; LogP, partition coefficient; TPSA, topological polar surface area; logKp, skin permeation coefficient.

Table S3. Serum biochemical parameters of treated animals at day 15.

Parameter	Unit	Copaiba-NE	BSLE-NE	Reference range (female rats)
ALT	U/L	26.0	22.0	16.53–37.95 U L^{-1}
AST	U/L	68.0	71.5	72.94–204.13 U L^{-1}
Creatinine	mg/dL	0.40	0.64	0.35–0.87 mg dL^{-1}
Urea	mg/dL	42.6	30.8	23.75–60.74 mg dL^{-1}

Serum biochemical parameters evaluated at day 15 (D15) following topical treatment with Copaiba-NE or BSLE-NE. Values are presented as means and interpreted descriptively because no inferential statistical analysis was performed. Reference intervals correspond to 10–14-week-old female *Wistar* rats [39].

Abbreviations: ALT, alanine aminotransferase; AST, aspartate aminotransferase.

Table S4. Hematological parameters of treated animals at day 15

Parameter	Unit	Copaiba-NE	BSLE-NE	Reference range
RBC	$\times 10^6/\mu L$	8.39	8.54	3.3–8.3
Hemoglobin	g/dL	11.4	11.7	7.2–16
Hematocrit	%	38	38	28–50
Total leukocytes	cells/μL	1,900	2,000	4,000–12,000
Neutrophils	cells/μL	209	240	1,200–5,400
Lymphocytes	cells/μL	1,691	1,740	4,800–9,840
Platelets	cells/μL	749,000	714,000	638,000–1,177,000

Hematological parameters evaluated at day 15 (D15) following topical treatment with Copaiba-NE or BSLE-NE. Values are presented as mean values and analyzed descriptively. The hematological profile showed reduced leukocyte and neutrophil counts with relative lymphocyte predominance, without association with biochemical alterations or clinical signs of systemic toxicity.

Abbreviations: RBC, red blood cells.

Table S5. Semi-quantitative histopathological scoring of wound healing at day 15

Parameter	Control	BSLE-NE	Copaiba-NE
Re-epithelialization	2	2	2
Epidermal thickness index (ETI)	0	1	1
Keratinization	0	2	2
Granulation tissue	1	2	1
Scar elevation index (SEI)	1	2	1
Tissue remodeling	1	2	1
Total score (0–12)	5	11	8

Semi-quantitative histopathological evaluation of wound healing at day 15 in the untreated control, BSLE-NE, and Copaiba-NE groups. Scoring was performed based on six parameters: re-epithelialization, epidermal thickness index, keratinization, granulation tissue, scar elevation index, and tissue remodeling. Each parameter was scored on a scale from 0 to 2, where higher scores indicate more advanced stages of tissue repair and structural organization. The total score represents the sum of all evaluated parameters (maximum score = 12).

Abbreviations: ETI, epidermal thickness index; SEI, scar elevation index.

Table S6: UHPLC–ESI–QTOF–MS chromatographic features detected in *B. suaveolens* (Willd.) Sweet hydroethanolic leaf extract and their annotation status.

Peak	RT (min)	Area	S/N	Maximum m/z	Putative annotation/status
1	1.1	16,496,522	103.3	172.1342	Unassigned
2	1.6	442,366	7.7	251.1411	Unassigned
3	1.8	19,734,634	148.6	256.1563	Unassigned
4	2.1	21,123,908	133.5	304.1567	Scopolamine
5	2.5	6,770,026	47.0	240.1615	3-Hydroxy-6-tigloyloxytropane
6	2.8	7,026,940	81.6	276.1615	Noratropine
7	3.0	14,314,350	197.2	292.1568	Unassigned
8	3.1	21,791,522	218.1	292.1569	Unassigned
9	3.3	3,750,527	46.5	535.2811	Unassigned
10	3.6	234,596	5.6	505.2695	Unassigned
11	3.8	709,940	9.5	373.2252	Unassigned
12	3.9	2,113,084	22.8	438.2432	Unassigned
13	4.5	157,577	4.2	496.2800	Unassigned
14	4.7	5,111,772	51.0	404.2104	Unassigned
15	4.9	16,172,703	82.5	404.2106	Unassigned
16	5.4	1,462,052	14.3	388.2160	Unassigned
17	5.7	2,117,106	40.7	584.2830	Unassigned
18	6.2	202,741	4.3	190.1238	Unassigned
19	6.8	179,702	3.8	158.1548	Unassigned
20	8.2	339,280	6.8	699.3648	Unassigned
21	9.0	1,997,631	13.5	353.2713	Unassigned
22	9.7	120,978	3.1	539.3247	Unassigned
23	10.1	2,916,711	18.8	301.1436	Unassigned
24	10.5	2,001,046	30.2	698.4609	Unassigned
25	11.0	468,235	8.4	425.2184	Unassigned
26	11.6	770,120	10.3	377.2694	Unassigned
27	13.2	3,118,510	46.0	593.2824	Unassigned
28	13.4	633,909	6.1	379.3234	Unassigned
29	14.0	736,455	11.6	613.4901	Unassigned
30	14.1	499,117	11.0	391.2879	Unassigned
31	14.3	1,382,037	18.0	381.3399	Unassigned
32	14.5	826,793	11.8	413.3664	Unassigned
33	14.7	10,689,036	150.6	621.3146	Unassigned
34	14.9	2,185,352	34.8	621.3145	Unassigned
35	15.1	1,158,782	16.6	613.4898	Unassigned
36	15.3	1,909,538	12.5	114.0916	Unassigned
37	15.7	4,606,070	71.1	429.3770	Unassigned
38	16.2	8,227,359	76.9	338.3442	Unassigned
39	17.9	4,258,030	36.8	410.4390	Unassigned

RT, retention time; S/N, signal-to-noise ratio; m/z, mass-to-charge ratio. Peak area represents the instrument-derived chromatographic response and was used to describe feature prominence rather than compound concentration. Compound-level putative annotations were restricted to peaks 4–6 based on the available accurate-mass, chromatographic, and literature evidence. The remaining features are reported as unassigned at the confidence level applied and were retained to document the complete qualitative chromatographic profile. The BSLE stock solution was prepared at 20 mg mL^{-1} in methanol–water (80:20, v/v), diluted 1:10 to a final concentration of 2 mg mL^{-1}, and filtered through a 0.22 μm membrane before injection. The commercial scopolamine butylbromide reference solution used for qualitative comparison had a concentration of 20 mg mL^{-1} (Hipolabor, Brazil).

Table S7. Chemical composition of copaiba oil (*Copaifera* sp.) determined by GC–MS.

Peak	RT (min)	Relative area (%)	Compound
1	5.875	0.37	δ-Elemene
2	5.998	0.69	α-Cubebene
3	6.309	6.33	α-Copaene
4	6.399	2.13	β-Elemene
5	6.781	48.91	β-Caryophyllene
6	7.090	6.88	α-Caryophyllene
7	7.207	1.95	γ-Muurolene
8	7.269	1.03	β-Farnesene
9	7.306	7.70	Germacrene D
10	7.393	2.22	β-Bisabolene
11	7.428	5.88	α-Himachalene
12	7.576	4.08	δ-Cadinene
13	8.022	1.00	γ-Elemene
14	8.233	1.37	Caryophyllene oxide
15	8.584	1.46	Cubenol
16	8.691	1.17	α-Muurolol
17	11.435	0.74	Palmitic acid
18	12.537	2.96	Linoleic acid
19	12.566	2.22	Elaidic acid
20	13.443	0.91	Unidentified

RT, retention time. Relative areas were calculated from integrated chromatographic peak areas. Compound identification was based on comparison with the NIST mass spectral library, considering similarity indices of 90–100%.

Supplementary Figures

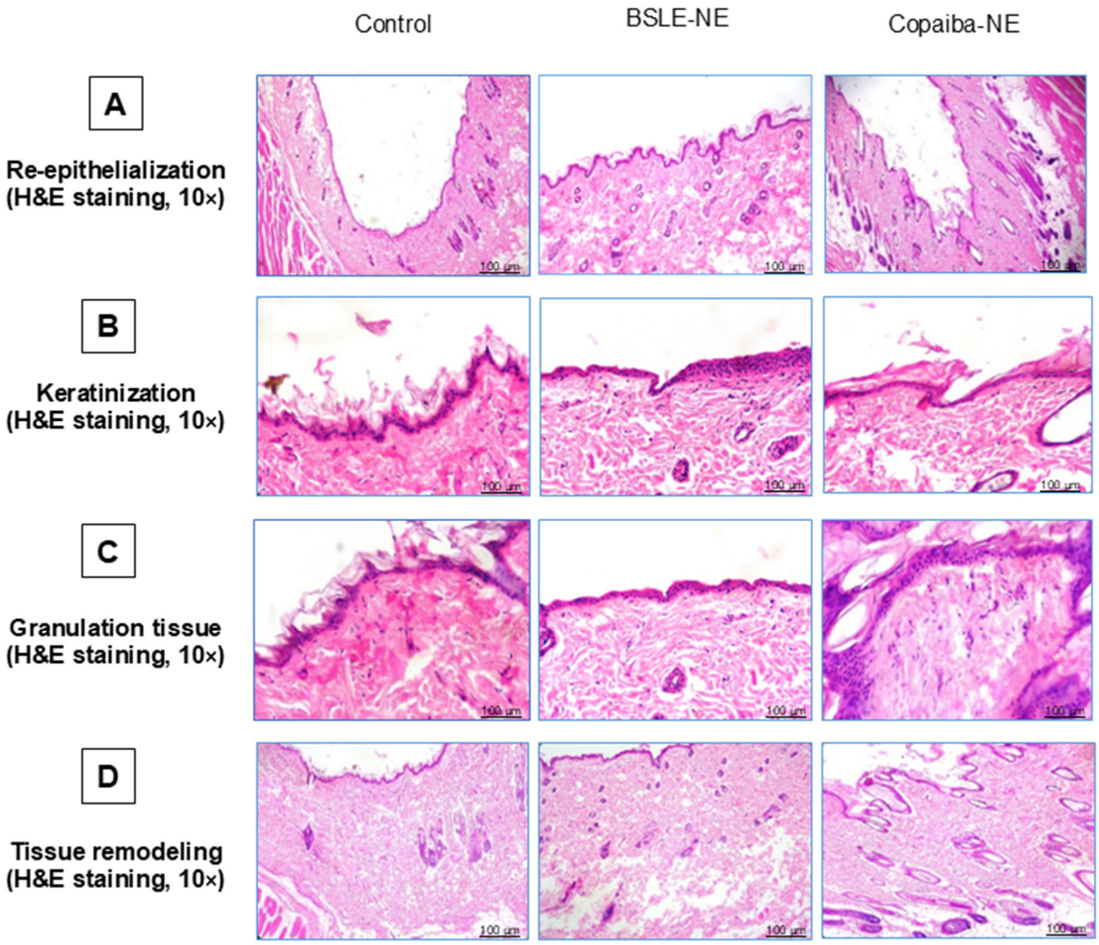

Figure S1. Comprehensive histopathological evaluation of wound healing in different treatment groups. Representative hematoxylin and eosin (H&E) stained sections of skin wounds from control, BSLE-NE, and Copaiba-NE groups at day 15 after injury. (A) Re-epithelialization, showing the extent of epithelial coverage over the wound surface. (B) Keratinization, indicating the presence or absence of a keratinized layer in the epidermis. (C) Granulation tissue, illustrating the presence, thickness, and organization of granulation tissue within the dermis. (D) Tissue remodeling, highlighting dermal organization, collagen distribution, and the presence of skin appendages. All images were acquired under identical conditions (H&E staining, 10× magnification) to allow qualitative comparison between groups.

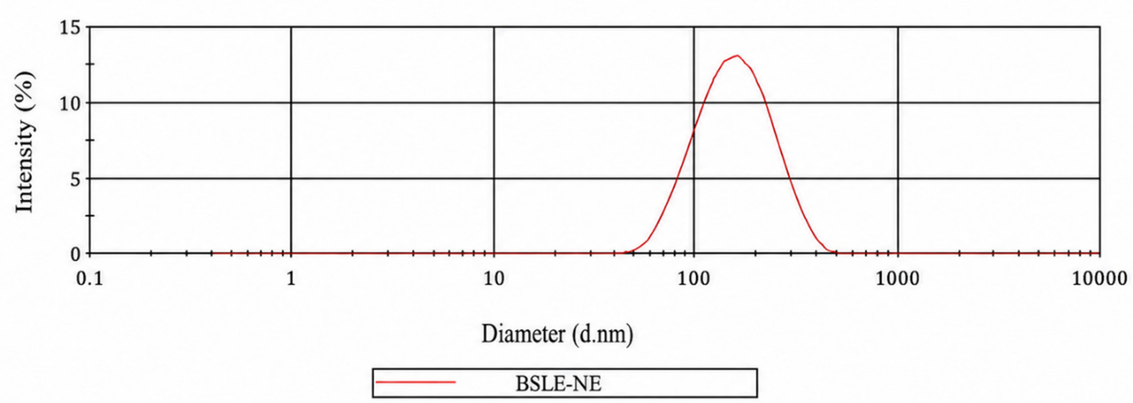

Figure S2. DLS size distribution of the optimized BSLE-NE**.** Representative dynamic light scattering profile of the optimized nanoemulsion containing *B. suaveolens* (Willd.) Sweet hydroethanolic leaf extract, showing a unimodal droplet size distribution in the nanometric range. The optimized BSLE-NE presented a mean droplet diameter of 144 nm and a polydispersity index of 0.135. BSLE-NE, nanoemulsion containing *B. suaveolens* hydroethanolic leaf extract; DLS, dynamic light scattering.

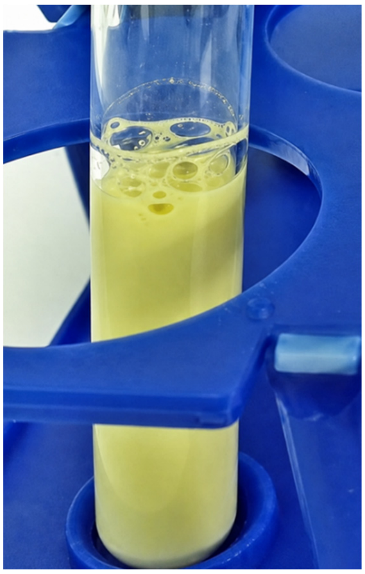

Figure S3. Macroscopic appearance of the optimized BSLE-NE. Representative image of the optimized nanoemulsion containing *B. suaveolens* (Willd.) Sweet hydroethanolic leaf extract, showing a homogeneous pale-yellow dispersion compatible with the formation of an oil-in-water nanoemulsion system. BSLE-NE, nanoemulsion containing *B. suaveolens* (Willd.) Sweet hydroethanolic leaf extract.

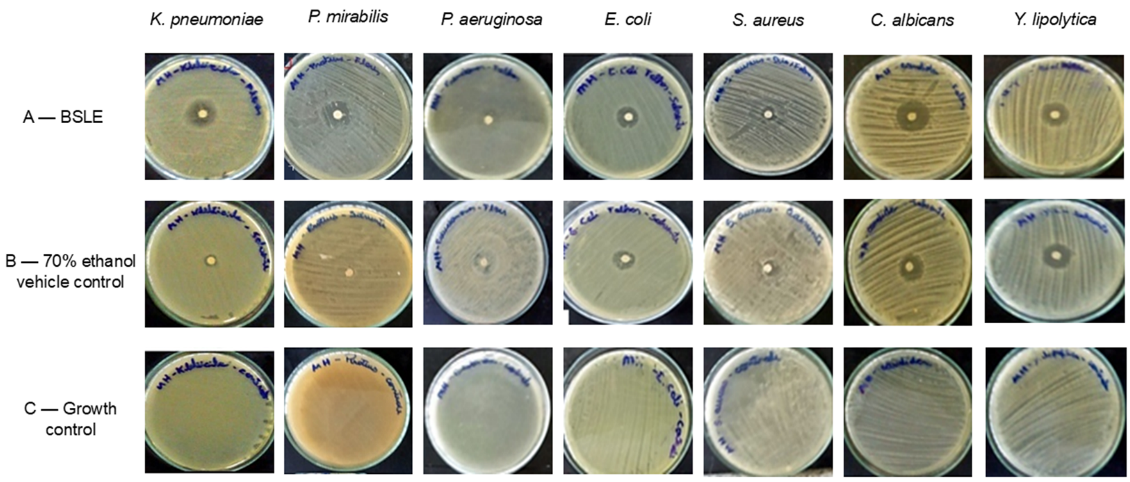

Figure S4. Representative agar disc-diffusion plates showing the antimicrobial activity of *Brugmansia suaveolens* (Willd.) Sweet hydroethanolic leaf extract (BSLE) against *Klebsiella pneumoniae*, *Proteus mirabilis*, *Pseudomonas aeruginosa* CCBH 04635, *Escherichia coli* CCBH 04631, *Staphylococcus aureus*, *Candida albicans*, and *Yarrowia lipolytica* IMUFRJ 50682. Columns correspond to the tested microorganisms, whereas rows show BSLE at 250 μg mL^{-1}, equivalent to 2.5 μg per 6 mm disc (A); the matched 70% (v/v) ethanol vehicle control (B); and the untreated microbial growth control (C). For each microorganism, the BSLE-treated plate shown corresponds to the replicate with an inhibition-zone diameter closest to the mean of three independent determinations. Quantitative inhibition indices presented in Figure 3 were calculated from all independent determinations (n = 3).
