# Supplementary material for: Dual-Component Nanoemulsion Containing Brugmansia suaveolens Leaf Extract and Copaiba Oil: Antimicrobial Activity and Preclinical Wound-Healing Potential
Source: Plants (Basel). 2026 Sep 23;15(19):2901. doi: 10.3390/plants15192901 (PMC13666830; doi:10.3390/plants15192901)

Correlogram Report
v2.0

Malvern Instruments Ltd - © Copyright 2008

Sample Details

Sample Name: NE OC - 1 P 1

SOP Name: mansettings.nano

General Notes:

**File Name:**	NE OC - 1 P.dts	**Dispersant Name:**	Water
**Record Number:**	1	**Dispersant RI:**	1,330
**Material RI:**	1,50	**Viscosity (cP):**	0,8872
**Material Absorbtion:**	0,000	**Measurement Date and Time:**	quarta-feira, 31 de julho ...

System

**Temperature (°C):**	25,0	**Duration Used (s):**	50
**Count Rate (kcps):**	366,0	**Measurement Position (mm):**	4,65
**Cell Description:**	Disposable sizing cuvette	**Attenuator:**	8

Results

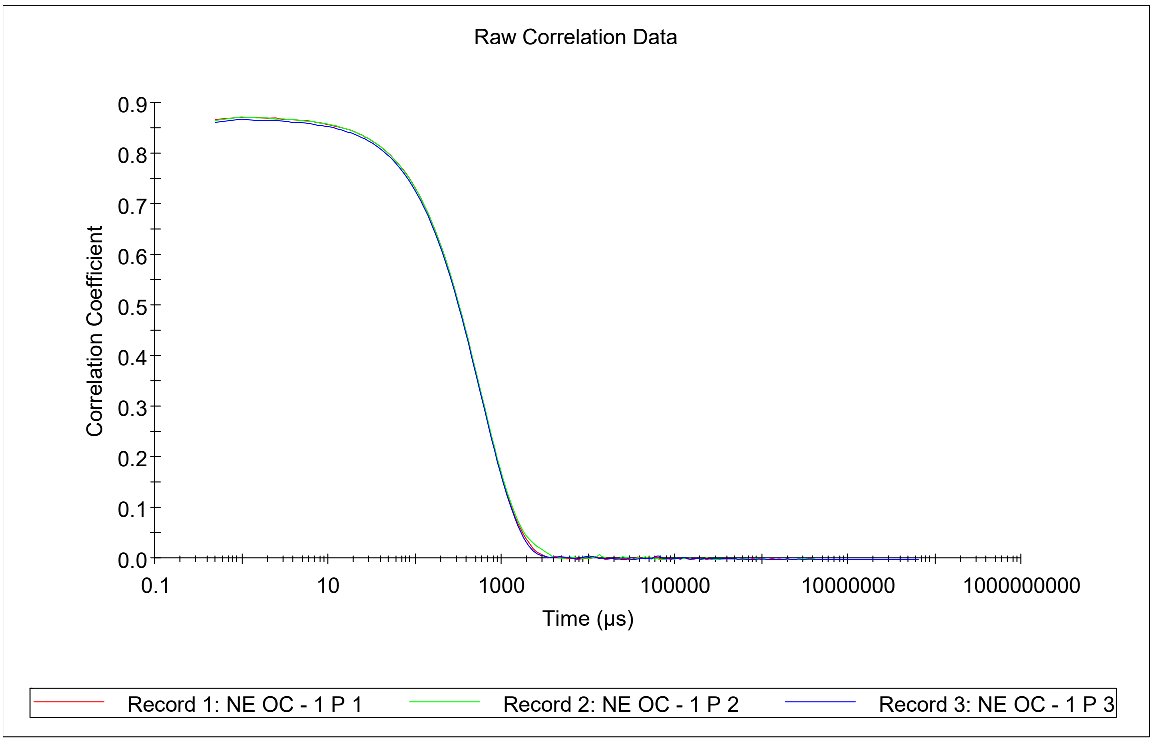

Size Distribution Report by Intensity

v2.2

Sample Details

Sample Name: NE OC - 1 P 1

SOP Name: mansettings.nano

General Notes:

**File Name:**	NE OC - 1 P.dts	**Dispersant Name:**	Water
**Record Number:**	1	**Dispersant RI:**	1,330
**Material RI:**	1,50	**Viscosity (cP):**	0,8872
**Material Absorbtion:**	0,000	**Measurement Date and Time:**	quarta-feira, 31 de julho de ...

System

**Temperature (°C):**	25,0	**Duration Used (s):**	50
**Count Rate (kcps):**	366,0	**Measurement Position (mm):**	4,65
**Cell Description:**	Disposable sizing cuvette	**Attenuator:**	8

Results

			Size (d.n...	% Intensity:	St Dev (d.n...
**Z-Average (d.nm):**	197,3	**Peak 1:**	217,4	100,0	69,06
**PdI:**	0,082	**Peak 2:**	0,000	0,0	0,000
**Intercept:**	0,933	**Peak 3:**	0,000	0,0	0,000

Result quality **Good**

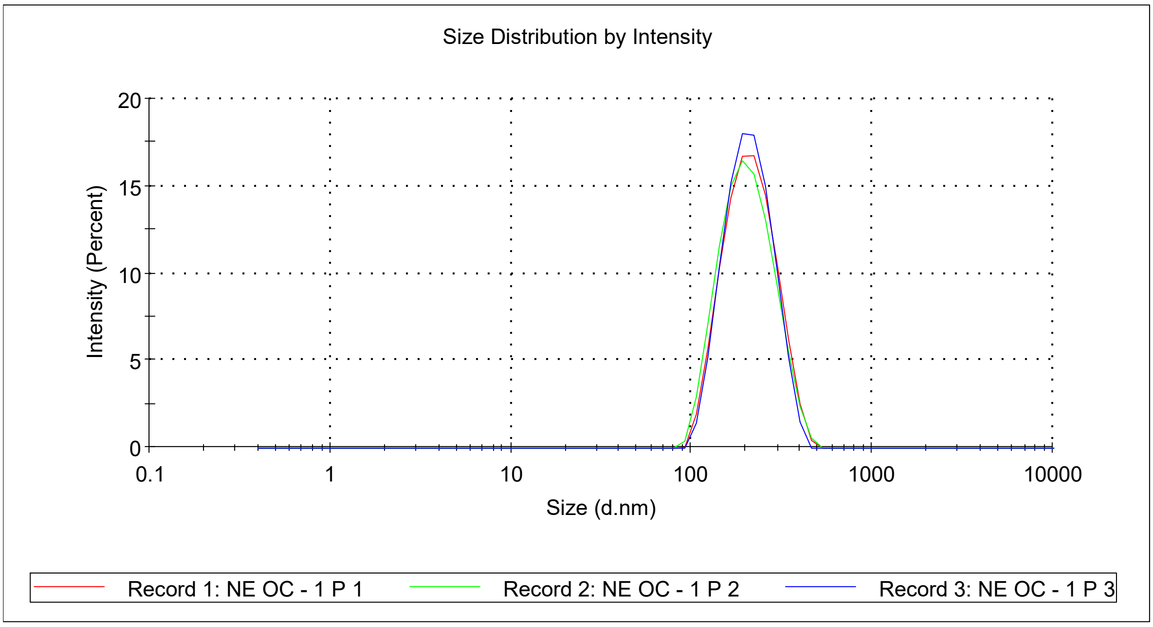

Size Quality Report

v2.0

Malvern Instruments Ltd - © Copyright 2008

Sample Name: NE OC - 1 P 1

SOP Name: mansettings.nano

File Name: NE OC - 1 P.dts

Record Number: 1

Measurement Date and Time: quarta-feira, 31 de julho de 2019 16:43:23

Temperature (°C): 25,0

RESULT MEETS QUALITY CRITERIA

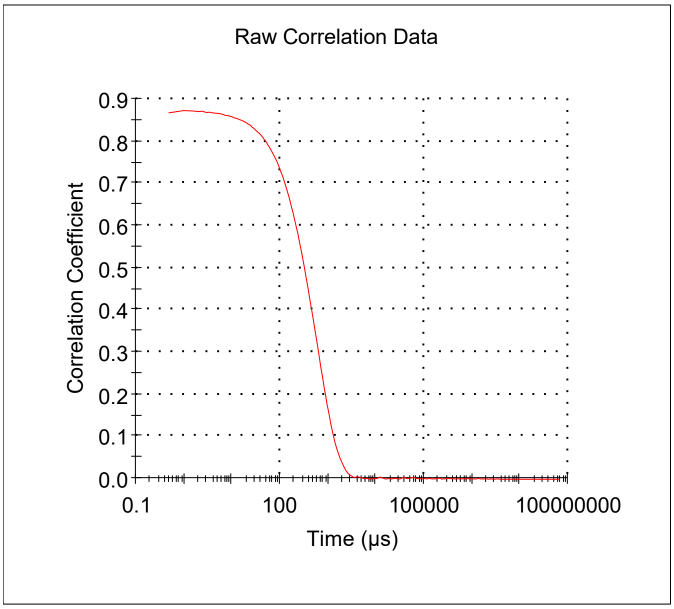

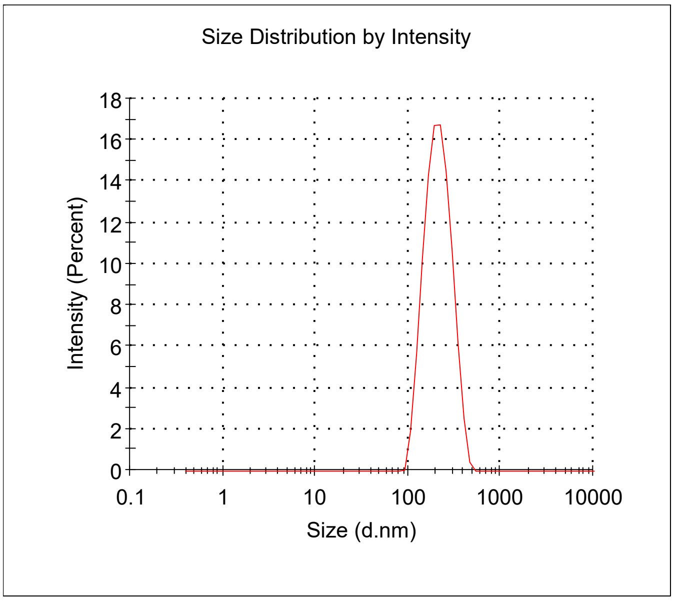

Size Distribution Report by Intensity

v2.2

Malvern

Sample Details

Sample Name: NE OC - 2 P 1

SOP Name: mansettings.nano

General Notes:

**File Name:**	NE OC - 2 P.dts	**Dispersant Name:**	Water
**Record Number:**	1	**Dispersant RI:**	1,330
**Material RI:**	1,50	**Viscosity (cP):**	0,8872
**Material Absorbtion:**	0,000	**Measurement Date and Time:**	quarta-feira, 31 de julho de ...

System

**Temperature (°C):**	25,0	**Duration Used (s):**	50
**Count Rate (kcps):**	227,8	**Measurement Position (mm):**	4,65
**Cell Description:**	Disposable sizing cuvette	**Attenuator:**	8

Results

			**Size (d.n...**	**% Intensity:**	**St Dev (d.n...**
**Z-Average (d.nm):**	196,2	**Peak 1:**	206,0	100,0	48,63
**PdI:**	0,023	**Peak 2:**	0,000	0,0	0,000
**Intercept:**	0,910	**Peak 3:**	0,000	0,0	0,000

Result quality **Good**

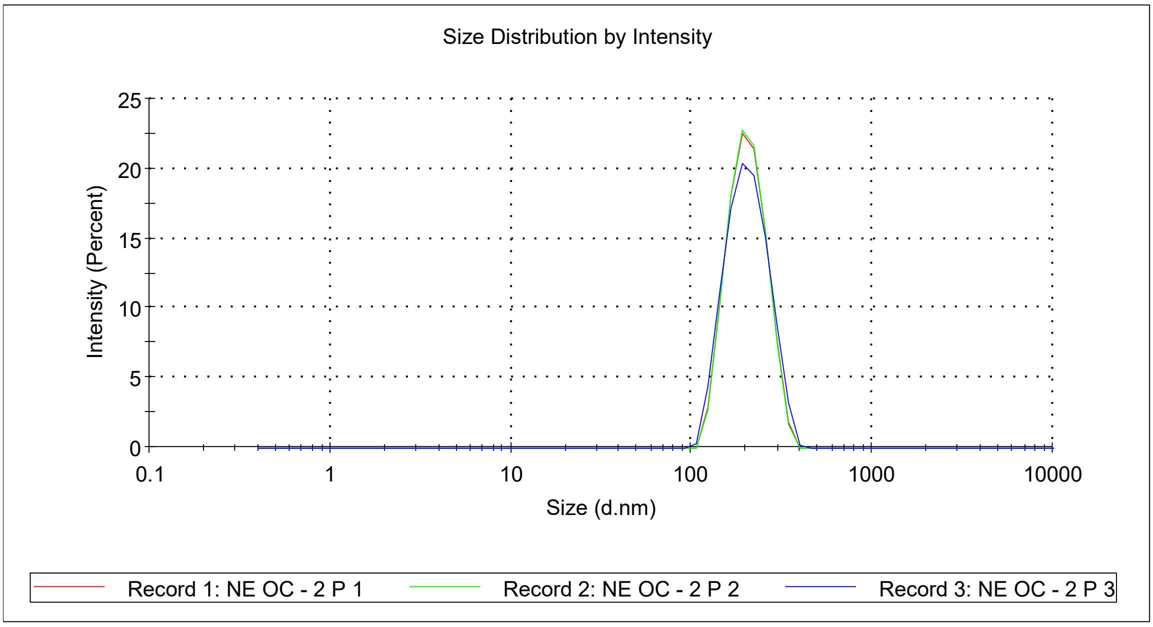

Size Quality Report

v2.0

Malvern Instruments Ltd - © Copyright 2008

Sample Name: NE OC - 2 P 1

SOP Name: mansettings.nano

File Name: NE OC - 2 P.dts

Record Number: 1

Measurement Date and Time: quarta-feira, 31 de julho de 2019 16:50:10

Temperature (°C): 25,0

RESULT MEETS QUALITY CRITERIA

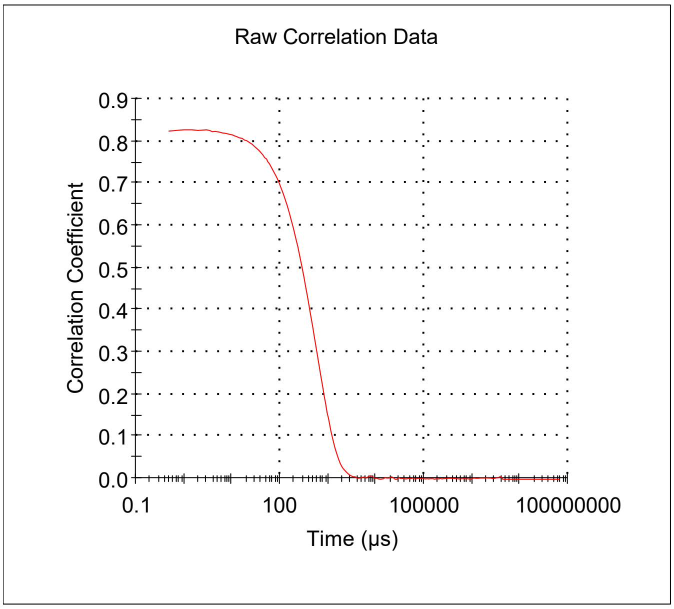

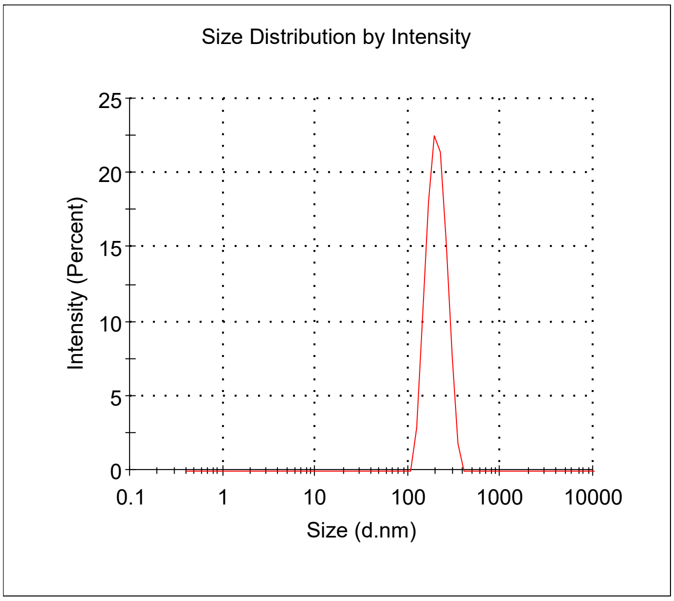

Correlogram Report

v2.0

Malvern Instruments Ltd - © Copyright 2008

Sample Details

Sample Name: NE OC - 2 P 1

SOP Name: mansettings.nano

General Notes:

**File Name:**	NE OC - 2 P.dts	**Dispersant Name:**	Water
**Record Number:**	1	**Dispersant RI:**	1,330
**Material RI:**	1,50	**Viscosity (cP):**	0,8872
**Material Absorbtion:**	0,000	**Measurement Date and Time:**	quarta-feira, 31 de julho ...

System

**Temperature (°C):**	25,0	**Duration Used (s):**	50
**Count Rate (kcps):**	227,8	**Measurement Position (mm):**	4,65
**Cell Description:**	Disposable sizing cuvette	**Attenuator:**	8

Results

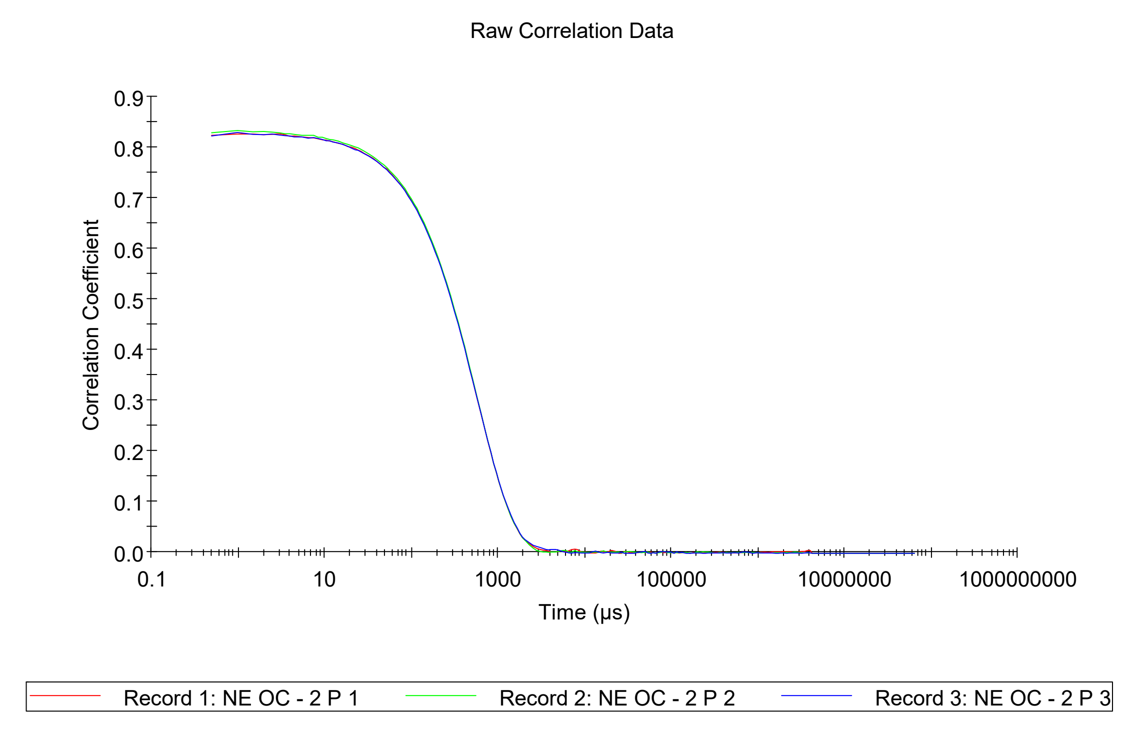

Correlogram Report

v2.0

Malvern Instruments Ltd - © Copyright 2008

Sample Details

Sample Name: NE OC - 3 P 1

SOP Name: mansettings.nano

General Notes:

**File Name:**	NE OC - 3 P.dts	**Dispersant Name:**	Water
**Record Number:**	1	**Dispersant RI:**	1,330
**Material RI:**	1,50	**Viscosity (cP):**	0,8872
**Material Absorbtion:**	0,000	**Measurement Date and Time:**	quarta-feira, 31 de julho ...

System

**Temperature (°C):**	25,0	**Duration Used (s):**	50
**Count Rate (kcps):**	301,2	**Measurement Position (mm):**	4,65
**Cell Description:**	Disposable sizing cuvette	**Attenuator:**	8

Results

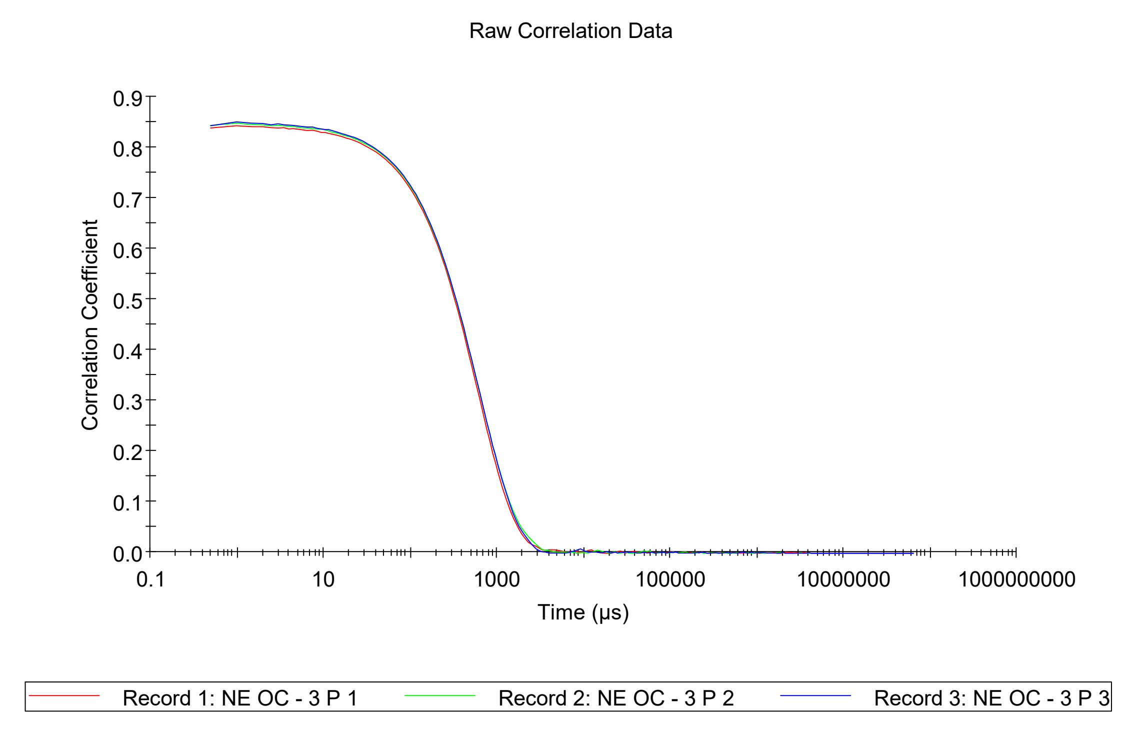

Size Distribution Report by Intensity

v2.2

Sample Details

Sample Name: NE OC - 3 P 1

SOP Name: mansettings.nano

General Notes:

**File Name:**	NE OC - 3 P.dts	**Dispersant Name:**	Water
**Record Number:**	1	**Dispersant RI:**	1,330
**Material RI:**	1,50	**Viscosity (cP):**	0,8872
**Material Absorbtion:**	0,000	**Measurement Date and Time:**	quarta-feira, 31 de julho de ...

System

**Temperature (°C):**	25,0	**Duration Used (s):**	50
**Count Rate (kcps):**	301,2	**Measurement Position (mm):**	4,65
**Cell Description:**	Disposable sizing cuvette	**Attenuator:**	8

Results

			Size (d.n...	% Intensity:	St Dev (d.n...
**Z-Average (d.nm):**	213,9	**Peak 1:**	219,6	100,0	42,33
**PdI:**	0,030	**Peak 2:**	0,000	0,0	0,000
**Intercept:**	0,917	**Peak 3:**	0,000	0,0	0,000

Result quality Good

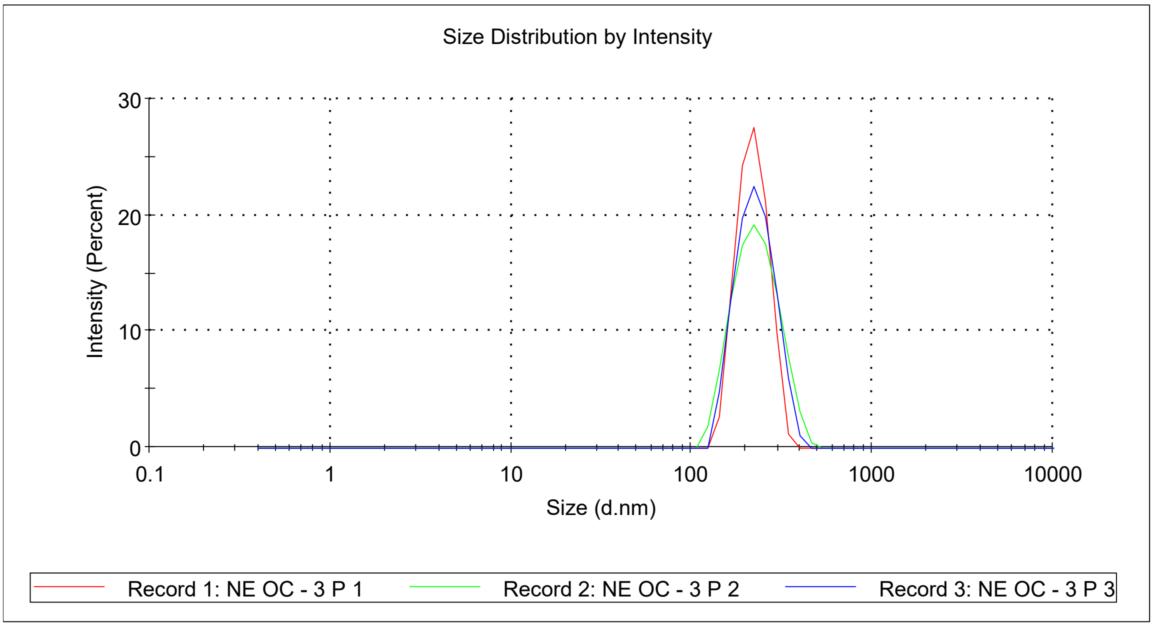

Size Quality Report

v2.0

Malvern Instruments Ltd - © Copyright 2008

Sample Name: NE OC - 3 P 1

SOP Name: mansettings.nano

File Name: NE OC - 3 P.dts

Record Number: 1

Measurement Date and Time: quarta-feira, 31 de julho de 2019 16:57:00

Temperature (°C): 25,0

RESULT MEETS QUALITY CRITERIA

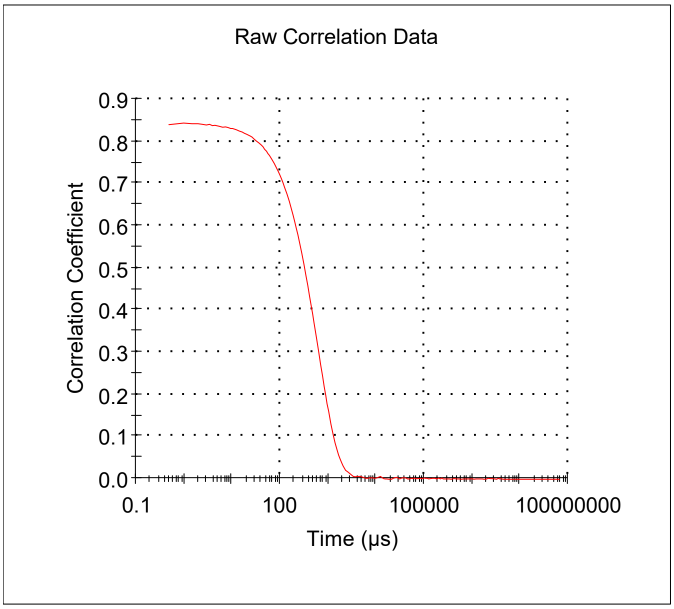

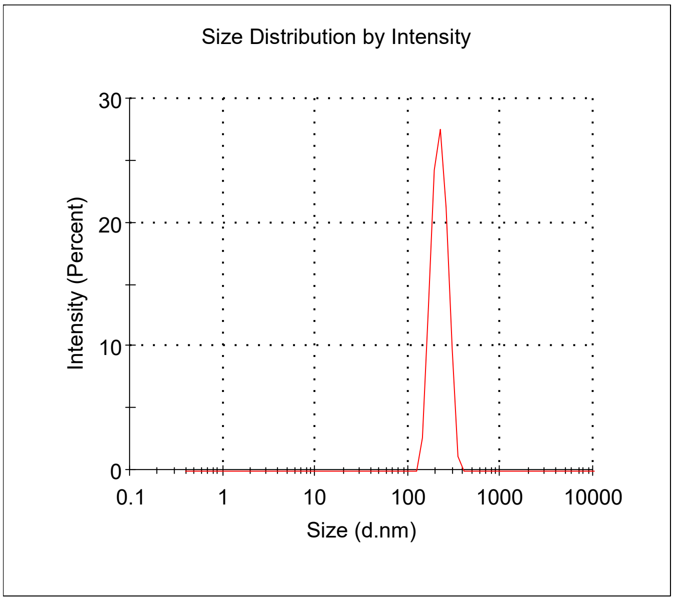

Size Distribution Report by Intensity

v2.2

Sample Details

Sample Name: NE OC - 4 P 1

SOP Name: mansettings.nano

General Notes:

**File Name:**	NE OC - 4 P.dts	**Dispersant Name:**	Water
**Record Number:**	1	**Dispersant RI:**	1,330
**Material RI:**	1,50	**Viscosity (cP):**	0,8872
**Material Absorbtion:**	0,000	**Measurement Date and Time:**	quarta-feira, 31 de julho de ...

System

**Temperature (°C):**	25,0	**Duration Used (s):**	50
**Count Rate (kcps):**	387,9	**Measurement Position (mm):**	4,65
**Cell Description:**	Disposable sizing cuvette	**Attenuator:**	8

Results

			Size (d.n...	% Intensity:	St Dev (d.n...
**Z-Average (d.nm):**	202,5	**Peak 1:**	211,6	100,0	45,18
**PdI:**	0,032	**Peak 2:**	0,000	0,0	0,000
**Intercept:**	0,912	**Peak 3:**	0,000	0,0	0,000

Result quality Good

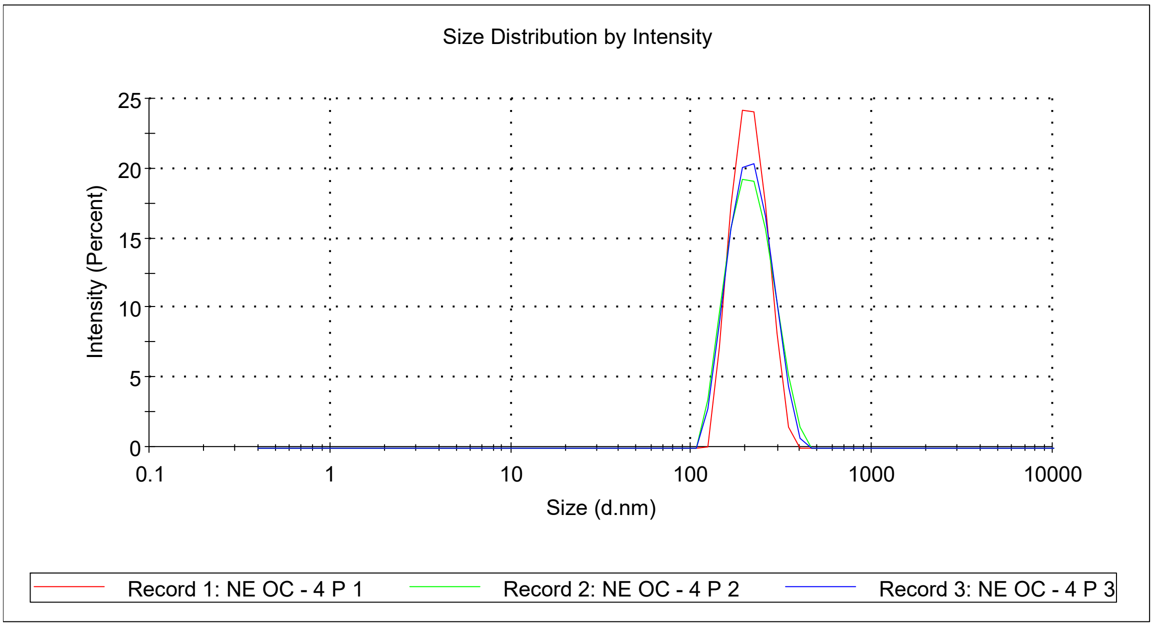

Size Quality Report

v2.0

Malvern Instruments Ltd - © Copyright 2008

Sample Name: NE OC - 4 P 1

SOP Name: mansettings.nano

File Name: NE OC - 4 P.dts

Record Number: 1

Measurement Date and Time: quarta-feira, 31 de julho de 2019 17:03:38

Temperature (°C): 25,0

RESULT MEETS QUALITY CRITERIA

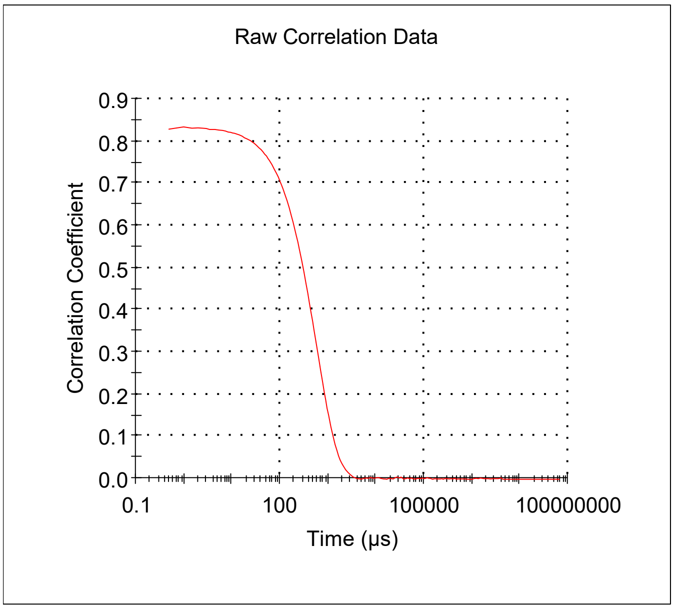

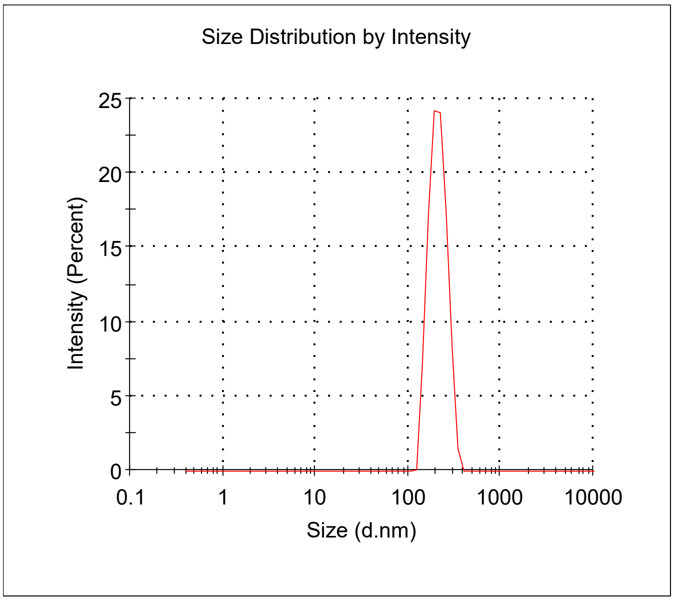

Correlogram Report

v2.0

Malvern Instruments Ltd - © Copyright 2008

Sample Details

Sample Name: NE OC - 4 P 1

SOP Name: mansettings.nano

General Notes:

**File Name:**	NE OC - 4 P.dts	**Dispersant Name:**	Water
**Record Number:**	1	**Dispersant RI:**	1,330
**Material RI:**	1,50	**Viscosity (cP):**	0,8872
**Material Absorbtion:**	0,000	**Measurement Date and Time:**	quarta-feira, 31 de julho ...

System

**Temperature (°C):**	25,0	**Duration Used (s):**	50
**Count Rate (kcps):**	387,9	**Measurement Position (mm):**	4,65
**Cell Description:**	Disposable sizing cuvette	**Attenuator:**	8

Results

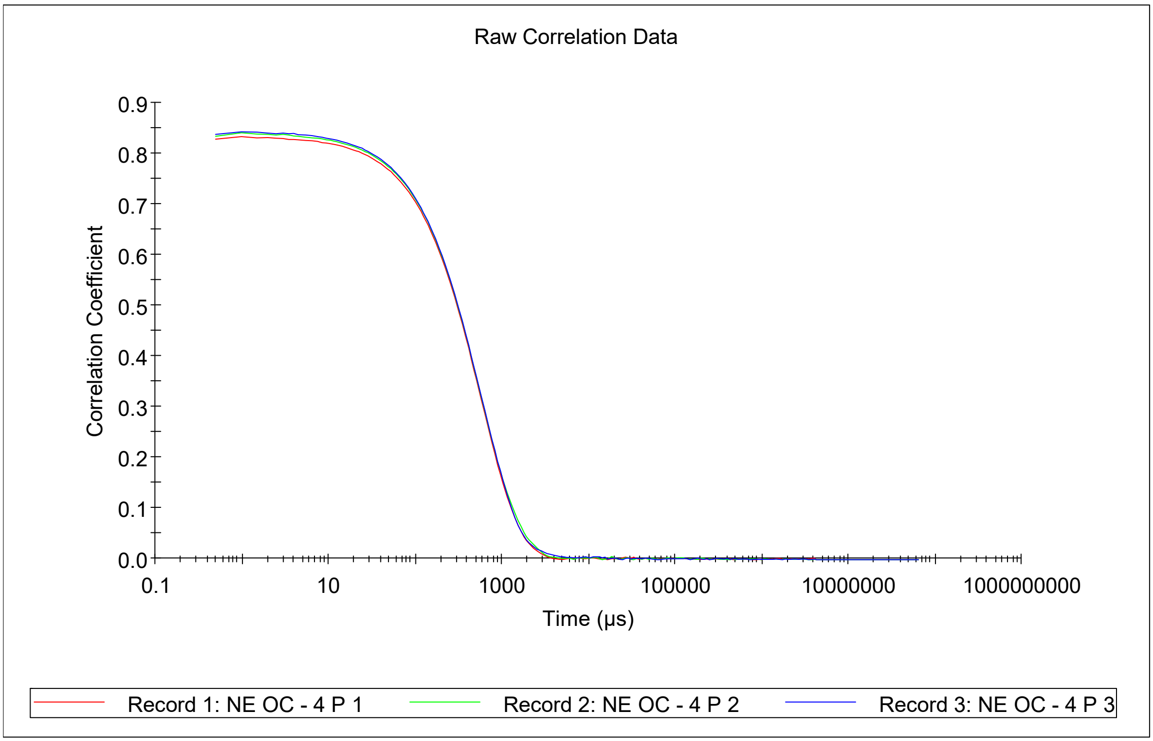

Size Distribution Report by Intensity

v2.2

Sample Details

**Sample Name:**	NE OC - 5 P 1
**SOP Name:**	mansettings.nano
**General Notes:**	

**File Name:**	NE OC - 5 P.dts	**Dispersant Name:**	Water
**Record Number:**	1	**Dispersant RI:**	1,330
**Material RI:**	1,50	**Viscosity (cP):**	0,8872
**Material Absorbtion:**	0,000	**Measurement Date and Time:**	quarta-feira, 31 de julho de ...

System

**Temperature (°C):**	25,0	**Duration Used (s):**	50
**Count Rate (kcps):**	220,7	**Measurement Position (mm):**	4,65
**Cell Description:**	Disposable sizing cuvette	**Attenuator:**	8

Results

			Size (d.n...	% Intensity:	St Dev (d.n...
**Z-Average (d.nm):**	230,9	**Peak 1:**	240,7	100,0	48,10
**PdI:**	0,035	**Peak 2:**	0,000	0,0	0,000
**Intercept:**	0,914	**Peak 3:**	0,000	0,0	0,000

Result quality **Good**

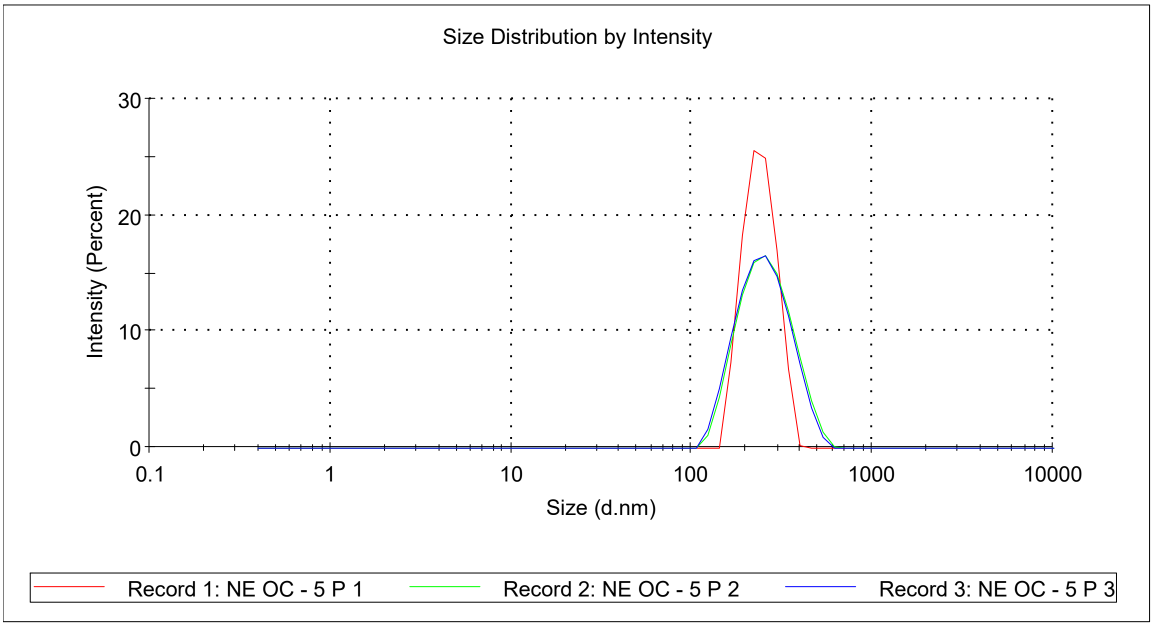

Size Quality Report

v2.0

Malvern Instruments Ltd - © Copyright 2008

Sample Name: NE OC - 5 P 1

SOP Name: mansettings.nano

File Name: NE OC - 5 P.dts

Record Number: 1

Measurement Date and Time: quarta-feira, 31 de julho de 2019 17:10:16

Temperature (°C): 25,0

RESULT MEETS QUALITY CRITERIA

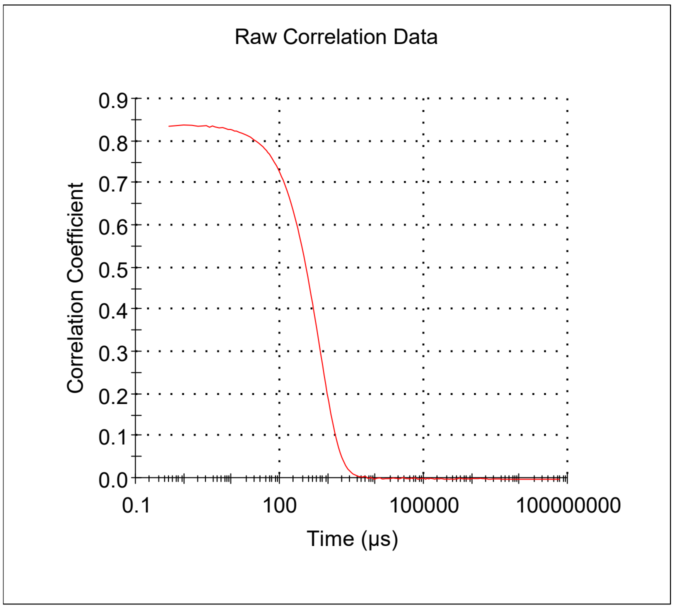

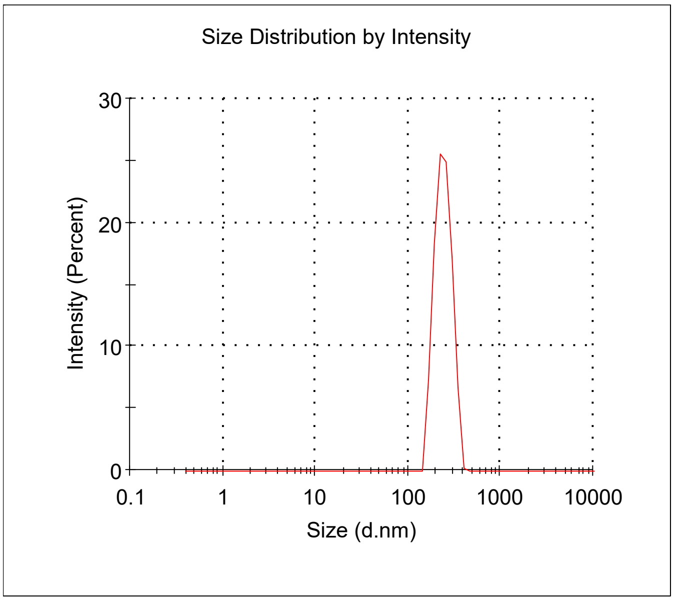

Correlogram Report

v2.0

Malvern Instruments Ltd - © Copyright 2008

Sample Details

Sample Name: NE OC - 5 P 1

SOP Name: mansettings.nano

General Notes:

**File Name:**	NE OC - 5 P.dts	**Dispersant Name:**	Water
**Record Number:**	1	**Dispersant RI:**	1,330
**Material RI:**	1,50	**Viscosity (cP):**	0,8872
**Material Absorbtion:**	0,000	**Measurement Date and Time:**	quarta-feira, 31 de julho ...

System

**Temperature (°C):**	25,0	**Duration Used (s):**	50
**Count Rate (kcps):**	220,7	**Measurement Position (mm):**	4,65
**Cell Description:**	Disposable sizing cuvette	**Attenuator:**	8

Results

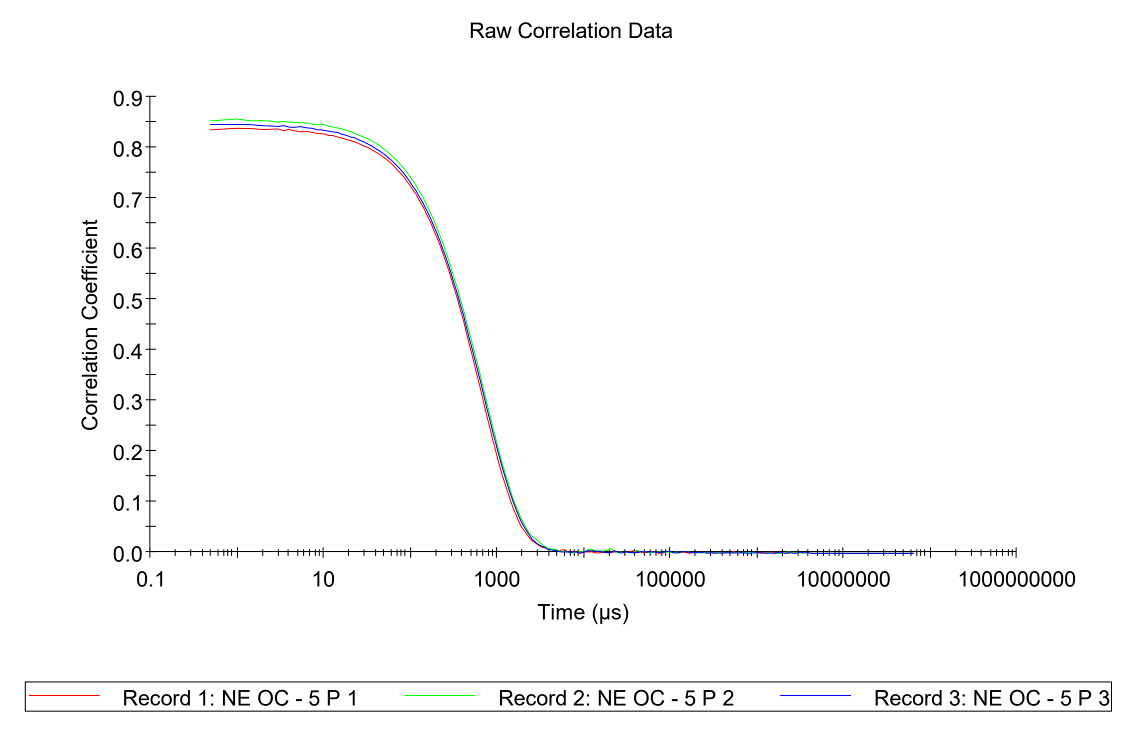

Size Distribution Report by Intensity

v2.2

Malvern

Sample Details

Sample Name: NE OC - 6 P 1

SOP Name: mansettings.nano

General Notes:

**File Name:**	NE OC - 6 P.dts	**Dispersant Name:**	Water
**Record Number:**	1	**Dispersant RI:**	1,330
**Material RI:**	1,50	**Viscosity (cP):**	0,8872
**Material Absorbtion:**	0,000	**Measurement Date and Time:**	quarta-feira, 31 de julho de ...

System

**Temperature (°C):**	25,0	**Duration Used (s):**	50
**Count Rate (kcps):**	222,8	**Measurement Position (mm):**	4,65
**Cell Description:**	Disposable sizing cuvette	**Attenuator:**	8

Results

			**Size (d.n...**	**% Intensity:**	**St Dev (d.n...**
**Z-Average (d.nm):**	231,0	**Peak 1:**	233,3	100,0	43,19
**PdI:**	0,101	**Peak 2:**	0,000	0,0	0,000
**Intercept:**	0,918	**Peak 3:**	0,000	0,0	0,000

Result quality **Good**

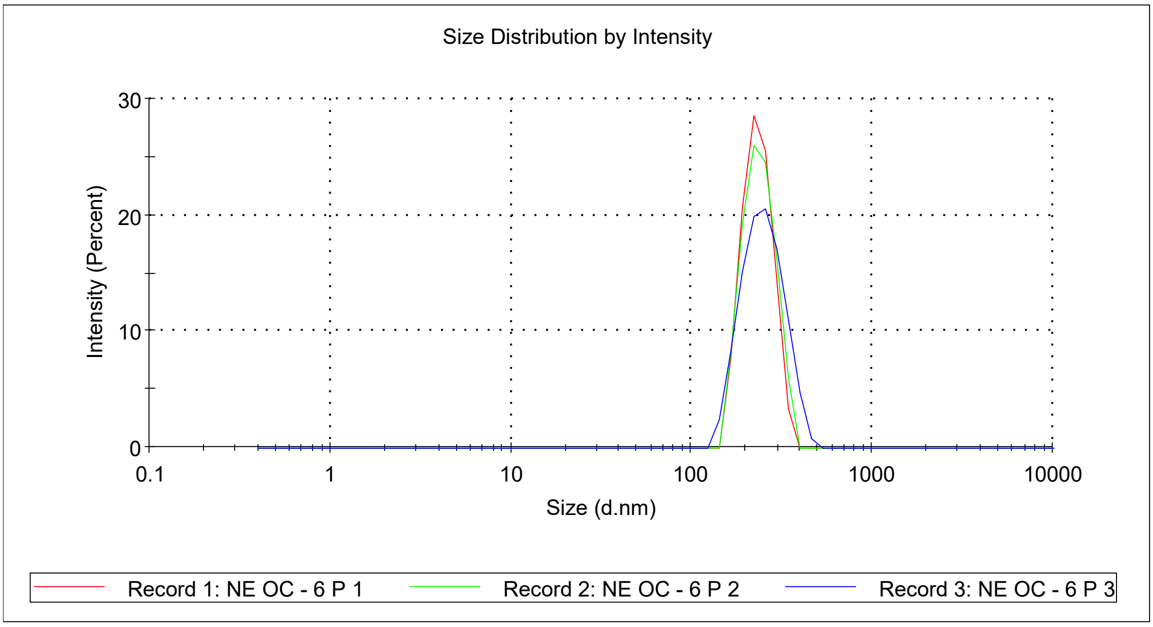

Size Quality Report

v2.0

Malvern Instruments Ltd - © Copyright 2008

Sample Name: NE OC - 6 P 1

SOP Name: mansettings.nano

File Name: NE OC - 6 P.dts

Record Number: 1

Measurement Date and Time: quarta-feira, 31 de julho de 2019 17:16:53

Temperature (°C): 25,0

RESULT MEETS QUALITY CRITERIA

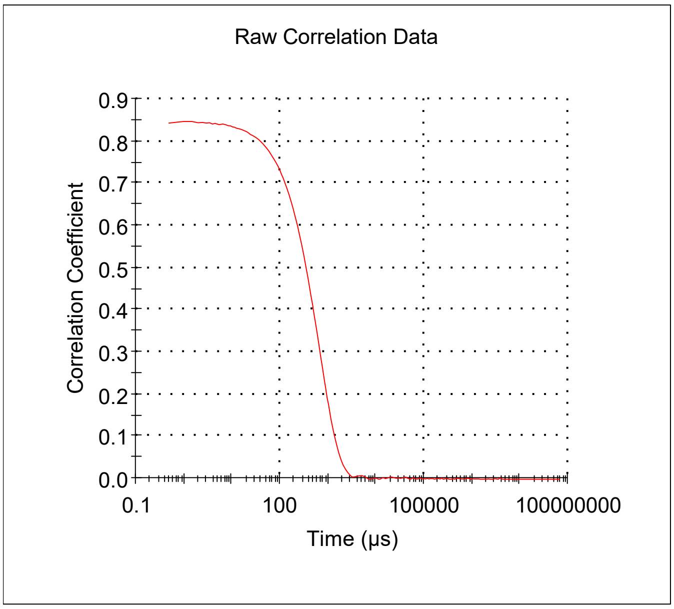

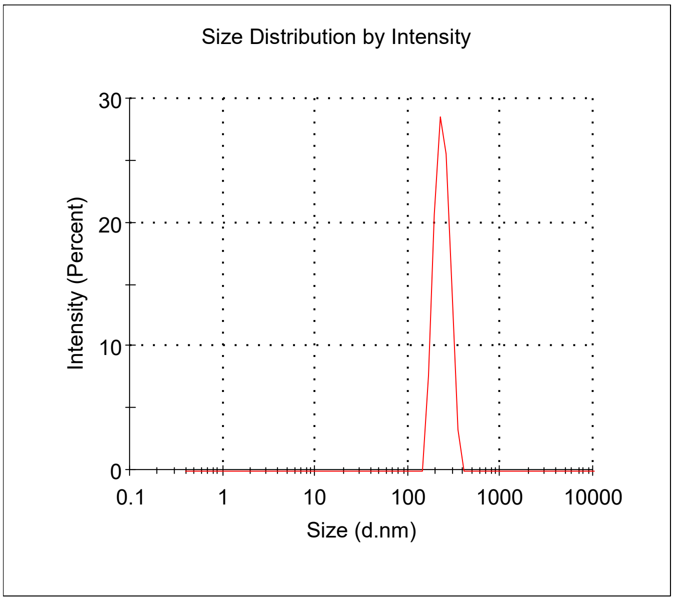

Correlogram Report

v2.0

Malvern Instruments Ltd - © Copyright 2008

Sample Details

Sample Name: NE OC - 6 P 1

SOP Name: mansettings.nano

General Notes:

**File Name:**	NE OC - 6 P.dts	**Dispersant Name:**	Water
**Record Number:**	1	**Dispersant RI:**	1,330
**Material RI:**	1,50	**Viscosity (cP):**	0,8872
**Material Absorbtion:**	0,000	**Measurement Date and Time:**	quarta-feira, 31 de julho ...

System

**Temperature (°C):**	25,0	**Duration Used (s):**	50
**Count Rate (kcps):**	222,8	**Measurement Position (mm):**	4,65
**Cell Description:**	Disposable sizing cuvette	**Attenuator:**	8

Results

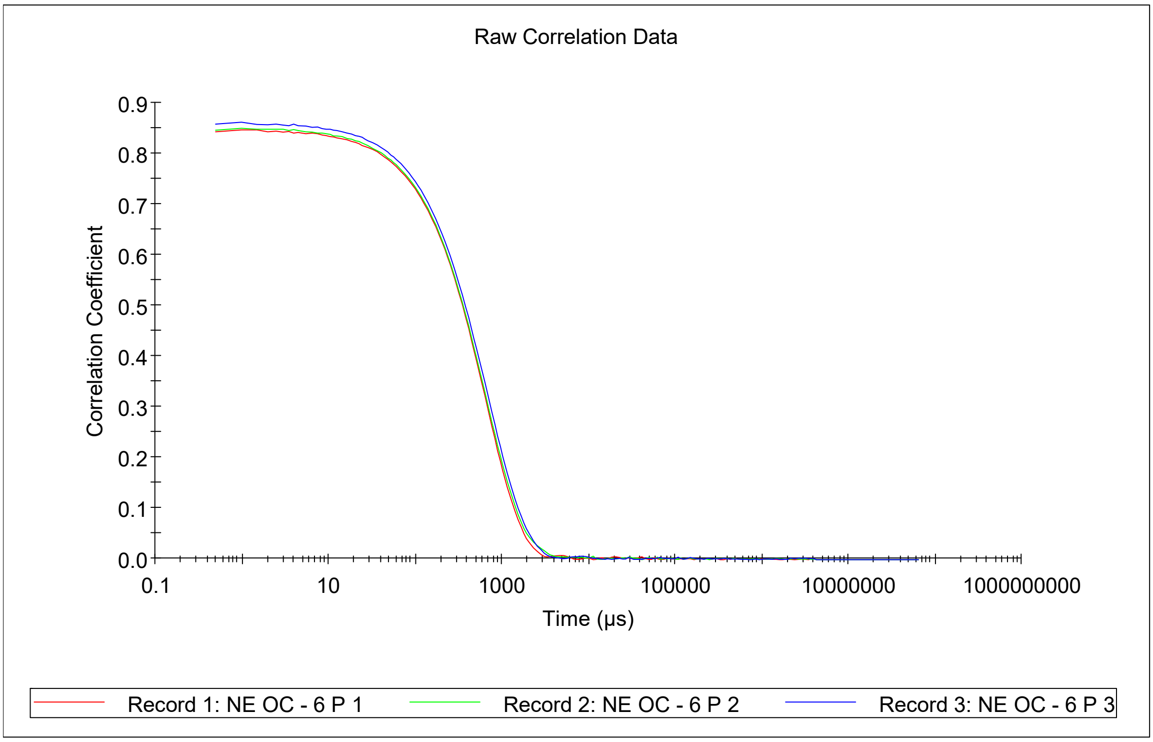

Size Distribution Report by Intensity

v2.2

Sample Details

**Sample Name:**	NE OC - 7 P 1
**SOP Name:**	mansettings.nano
**General Notes:**	

**File Name:**	NE OC - 7 P.dts	**Dispersant Name:**	Water
**Record Number:**	1	**Dispersant RI:**	1,330
**Material RI:**	1,50	**Viscosity (cP):**	0,8872
**Material Absorbtion:**	0,000	**Measurement Date and Time:**	quarta-feira, 31 de julho de ...

System

**Temperature (°C):**	25,0	**Duration Used (s):**	50
**Count Rate (kcps):**	384,9	**Measurement Position (mm):**	4,65
**Cell Description:**	Disposable sizing cuvette	**Attenuator:**	8

Results

			Size (d.n...	% Intensity:	St Dev (d.n...
**Z-Average (d.nm):**	223,2	**Peak 1:**	230,9	100,0	47,63
**PdI:**	0,006	**Peak 2:**	0,000	0,0	0,000
**Intercept:**	0,923	**Peak 3:**	0,000	0,0	0,000

Result quality Good

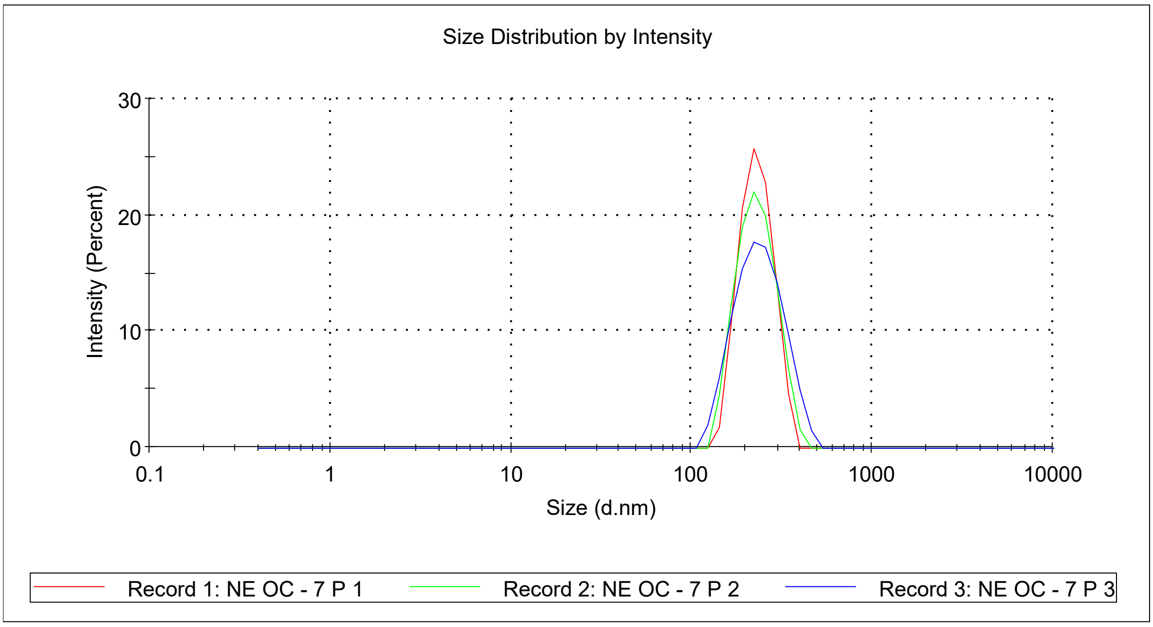

Size Quality Report

v2.0

Malvern Instruments Ltd - © Copyright 2008

Sample Name: NE OC - 7 P 1

SOP Name: mansettings.nano

File Name: NE OC - 7 P.dts

Record Number: 1

Measurement Date and Time: quarta-feira, 31 de julho de 2019 17:23:34

Temperature (°C): 25,0

RESULT MEETS QUALITY CRITERIA

Raw Correlation Data

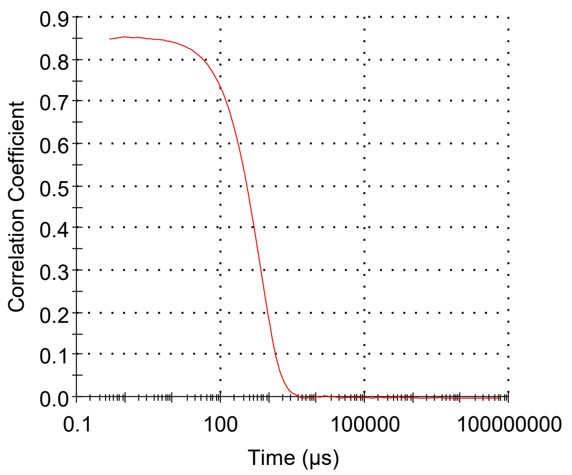

Size Distribution by Intensity

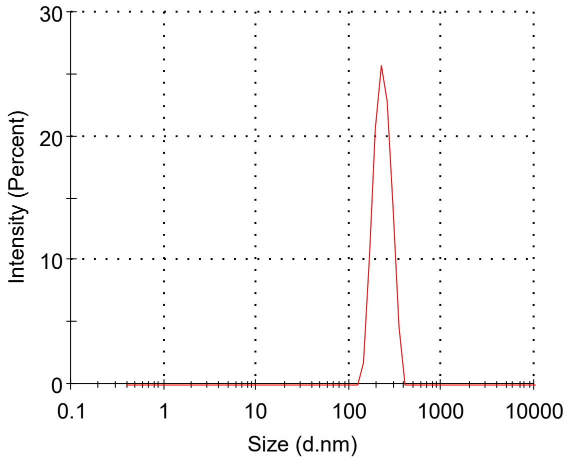

Correlogram Report

v2.0

Malvern Instruments Ltd - © Copyright 2008

Sample Details

Sample Name: NE OC - 7 P 1

SOP Name: mansettings.nano

General Notes:

**File Name:**	NE OC - 7 P.dts	**Dispersant Name:**	Water
**Record Number:**	1	**Dispersant RI:**	1,330
**Material RI:**	1,50	**Viscosity (cP):**	0,8872
**Material Absorbtion:**	0,000	**Measurement Date and Time:**	quarta-feira, 31 de julho ...

System

**Temperature (°C):**	25,0	**Duration Used (s):**	50
**Count Rate (kcps):**	384,9	**Measurement Position (mm):**	4,65
**Cell Description:**	Disposable sizing cuvette	**Attenuator:**	8

Results

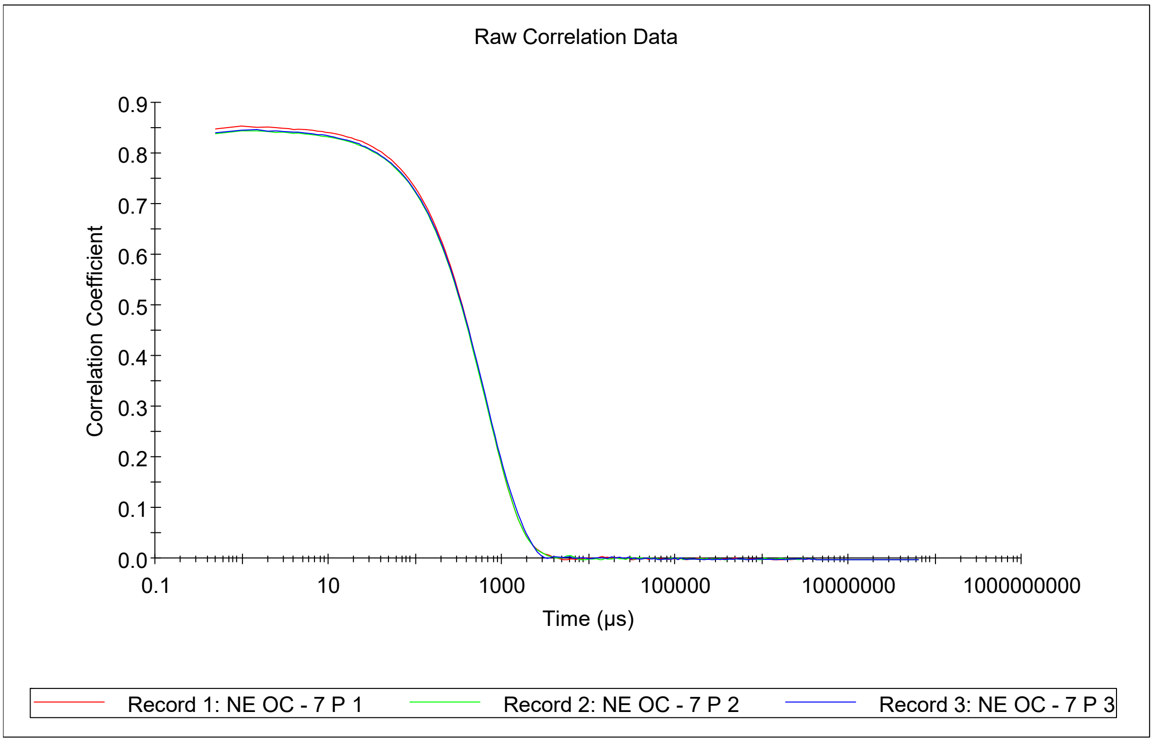

Correlogram Report

v2.0

Malvern Instruments Ltd - © Copyright 2008

Sample Details

Sample Name: NE OC - 8 P 1

SOP Name: mansettings.nano

General Notes:

**File Name:**	NE OC - 8 P.dts	**Dispersant Name:**	Water
**Record Number:**	1	**Dispersant RI:**	1,330
**Material RI:**	1,50	**Viscosity (cP):**	0,8872
**Material Absorbtion:**	0,000	**Measurement Date and Time:**	quarta-feira, 31 de julho ...

System

**Temperature (°C):**	25,0	**Duration Used (s):**	50
**Count Rate (kcps):**	236,3	**Measurement Position (mm):**	4,65
**Cell Description:**	Disposable sizing cuvette	**Attenuator:**	8

Results

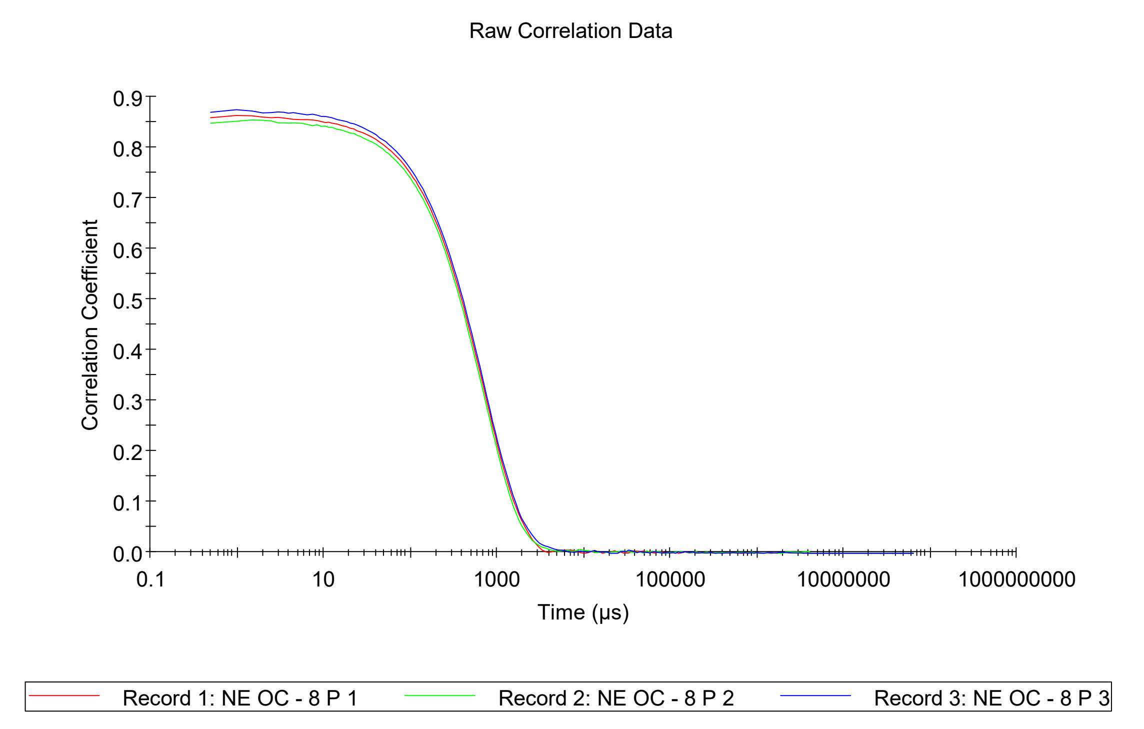

Size Distribution Report by Intensity

v2.2

Malvern

Sample Details

**Sample Name:**	NE OC - 8 P 1
**SOP Name:**	mansettings.nano
**General Notes:**	

**File Name:**	NE OC - 8 P.dts	**Dispersant Name:**	Water
**Record Number:**	1	**Dispersant RI:**	1,330
**Material RI:**	1,50	**Viscosity (cP):**	0,8872
**Material Absorbtion:**	0,000	**Measurement Date and Time:**	quarta-feira, 31 de julho de ...

System

**Temperature (°C):**	25,0	**Duration Used (s):**	50
**Count Rate (kcps):**	236,3	**Measurement Position (mm):**	4,65
**Cell Description:**	Disposable sizing cuvette	**Attenuator:**	8

Results

			**Size (d.n...**	**% Intensity:**	**St Dev (d.n...**
**Z-Average (d.nm):**	244,7	**Peak 1:**	259,0	100,0	64,76
**PdI:**	0,038	**Peak 2:**	0,000	0,0	0,000
**Intercept:**	0,927	**Peak 3:**	0,000	0,0	0,000

Result quality Good

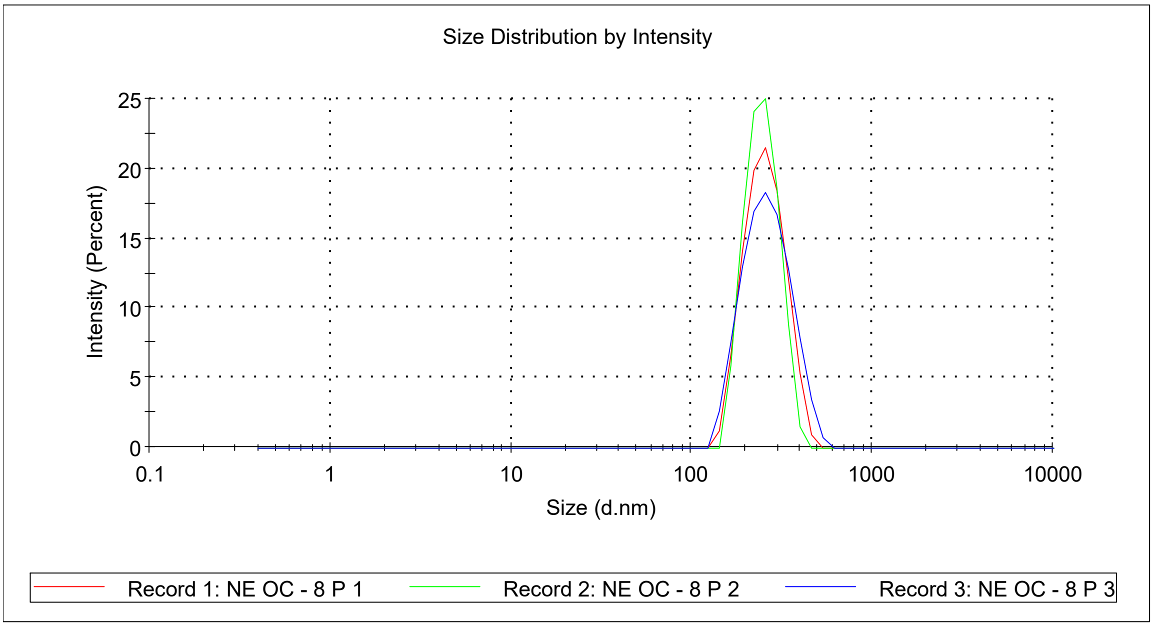

Size Quality Report

v2.0

Malvern Instruments Ltd - © Copyright 2008

Sample Name: NE OC - 8 P 1

SOP Name: mansettings.nano

File Name: NE OC - 8 P.dts

Record Number: 1

Measurement Date and Time: quarta-feira, 31 de julho de 2019 17:30:13

Temperature (°C): 25,0

RESULT MEETS QUALITY CRITERIA

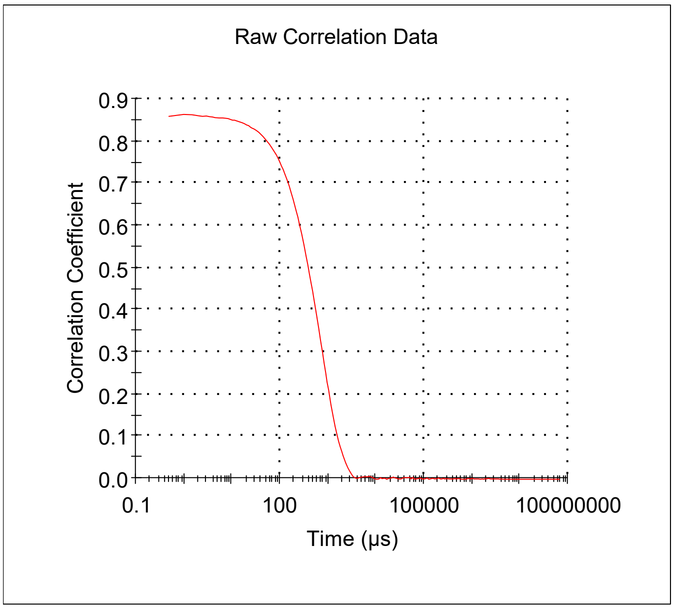

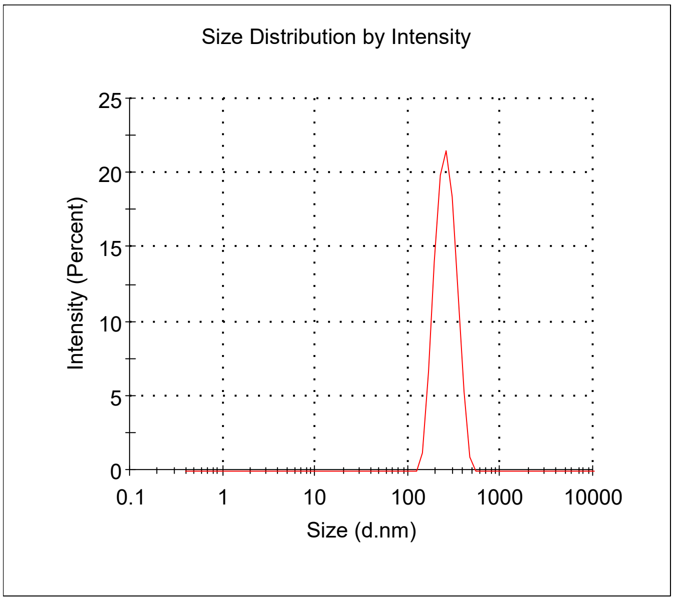

Size Distribution Report by Intensity

v2.2

Malvern

Sample Details

Sample Name: NE OC - 9 P 1

SOP Name: mansettings.nano

General Notes:

**File Name:**	NE OC - 9 P.dts	**Dispersant Name:**	Water
**Record Number:**	1	**Dispersant RI:**	1,330
**Material RI:**	1,50	**Viscosity (cP):**	0,8872
**Material Absorbtion:**	0,000	**Measurement Date and Time:**	quarta-feira, 31 de julho de ...

System

**Temperature (°C):**	25,0	**Duration Used (s):**	50
**Count Rate (kcps):**	195,2	**Measurement Position (mm):**	4,65
**Cell Description:**	Disposable sizing cuvette	**Attenuator:**	8

Results

			Size (d.n...	% Intensity:	St Dev (d.n...
**Z-Average (d.nm):**	228,5	**Peak 1:**	239,3	100,0	53,62
**PdI:**	0,027	**Peak 2:**	0,000	0,0	0,000
**Intercept:**	0,926	**Peak 3:**	0,000	0,0	0,000

Result quality Good

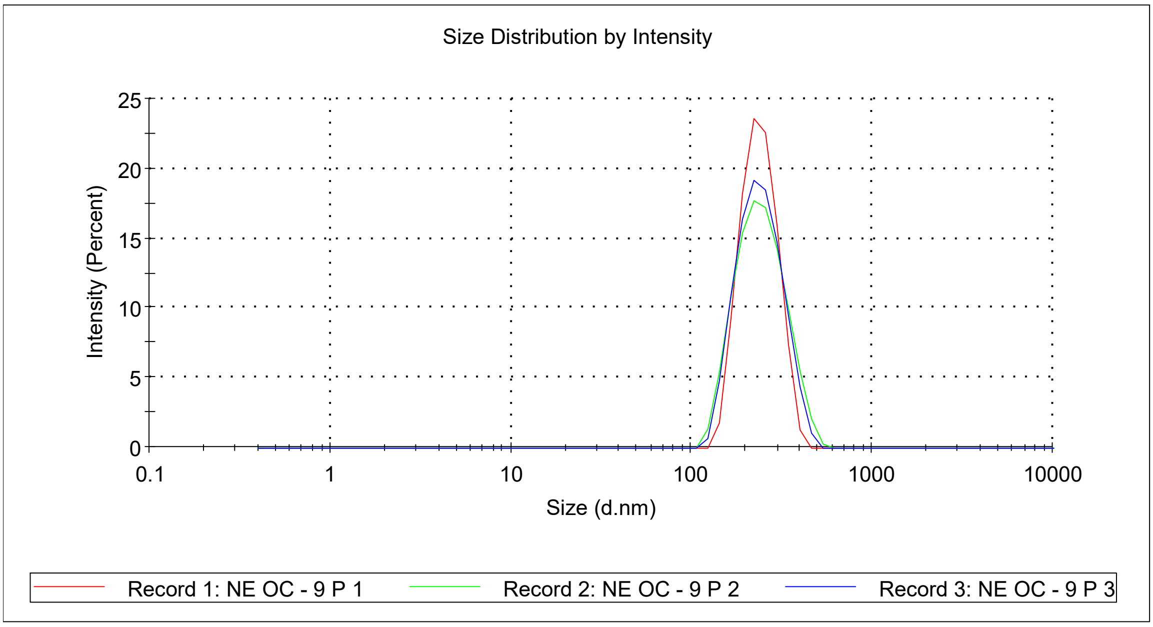

Size Quality Report

v2.0

Malvern Instruments Ltd - © Copyright 2008

Sample Name: NE OC - 9 P 1

SOP Name: mansettings.nano

File Name: NE OC - 9 P.dts

Record Number: 1

Measurement Date and Time: quarta-feira, 31 de julho de 2019 17:36:59

Temperature (°C): 25,0

RESULT MEETS QUALITY CRITERIA

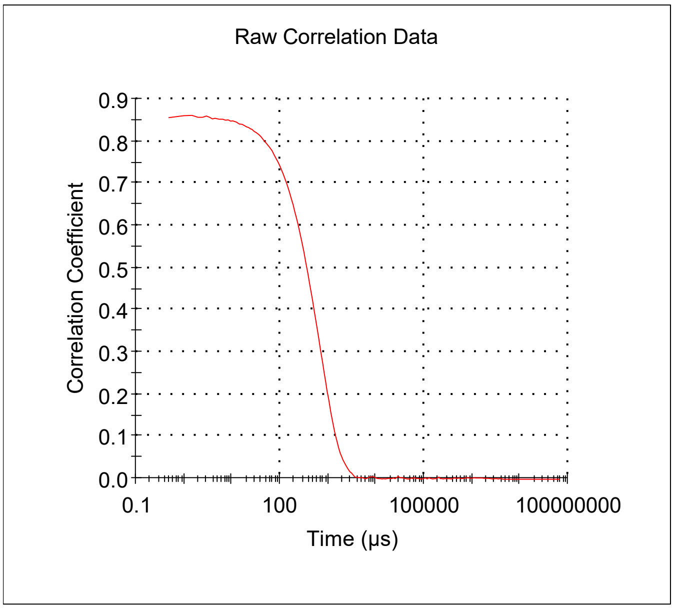

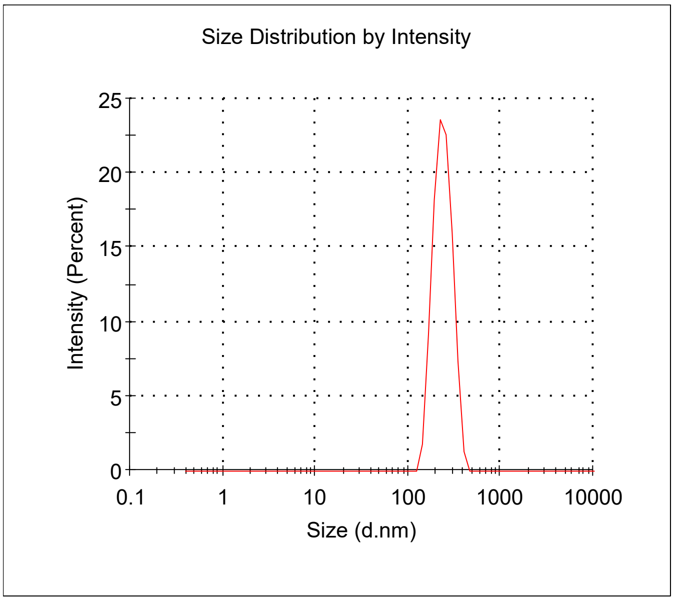

Correlogram Report

v2.0

Malvern Instruments Ltd - © Copyright 2008

Sample Details

Sample Name: NE OC - 9 P 1

SOP Name: mansettings.nano

General Notes:

**File Name:**	NE OC - 9 P.dts	**Dispersant Name:**	Water
**Record Number:**	1	**Dispersant RI:**	1,330
**Material RI:**	1,50	**Viscosity (cP):**	0,8872
**Material Absorbtion:**	0,000	**Measurement Date and Time:**	quarta-feira, 31 de julho ...

System

**Temperature (°C):**	25,0	**Duration Used (s):**	50
**Count Rate (kcps):**	195,2	**Measurement Position (mm):**	4,65
**Cell Description:**	Disposable sizing cuvette	**Attenuator:**	8

Results

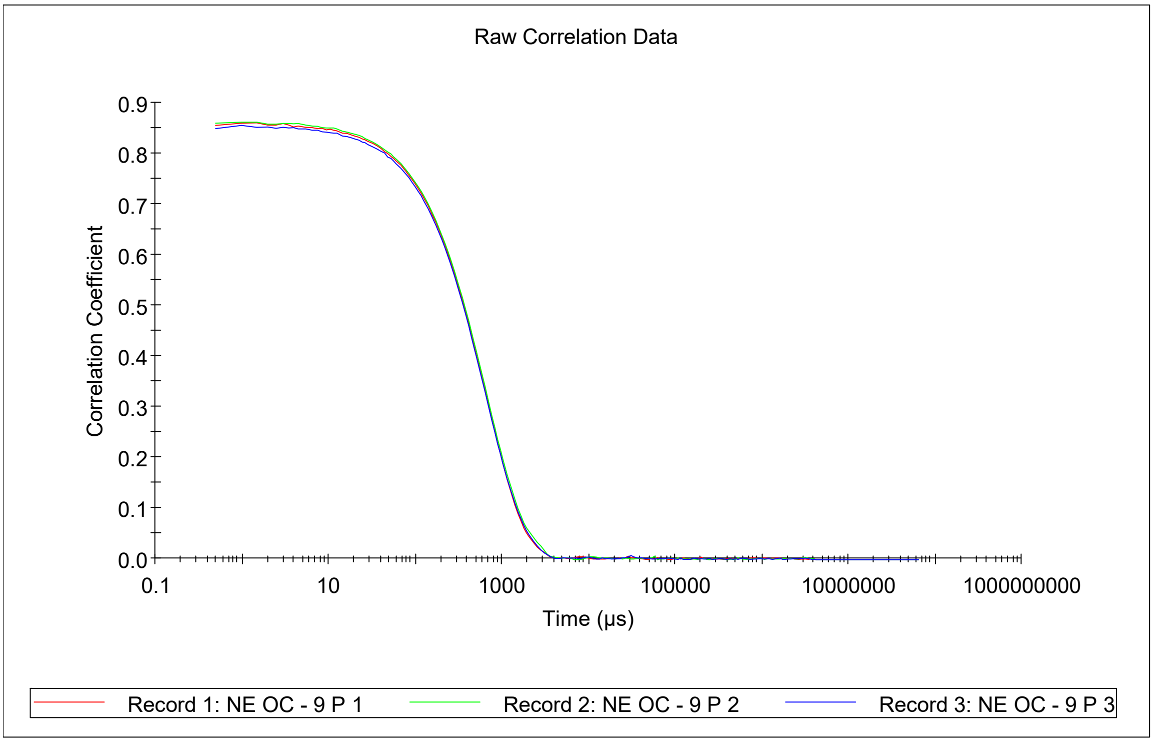

Size Distribution Report by Intensity

v2.2

Sample Details

Sample Name: NE OC - 10 P 1

SOP Name: mansettings.nano

General Notes:

**File Name:**	NE OC - 10 P.dts	**Dispersant Name:**	Water
**Record Number:**	1	**Dispersant RI:**	1,330
**Material RI:**	1,50	**Viscosity (cP):**	0,8872
**Material Absorbtion:**	0,000	**Measurement Date and Time:**	quarta-feira, 31 de julho de ...

System

**Temperature (°C):**	25,0	**Duration Used (s):**	50
**Count Rate (kcps):**	244,0	**Measurement Position (mm):**	4,65
**Cell Description:**	Disposable sizing cuvette	**Attenuator:**	8

Results

			Size (d.n...	% Intensity:	St Dev (d.n...
**Z-Average (d.nm):**	230,0	**Peak 1:**	249,2	100,0	85,53
**PdI:**	0,148	**Peak 2:**	0,000	0,0	0,000
**Intercept:**	0,921	**Peak 3:**	0,000	0,0	0,000

Result quality **Good**

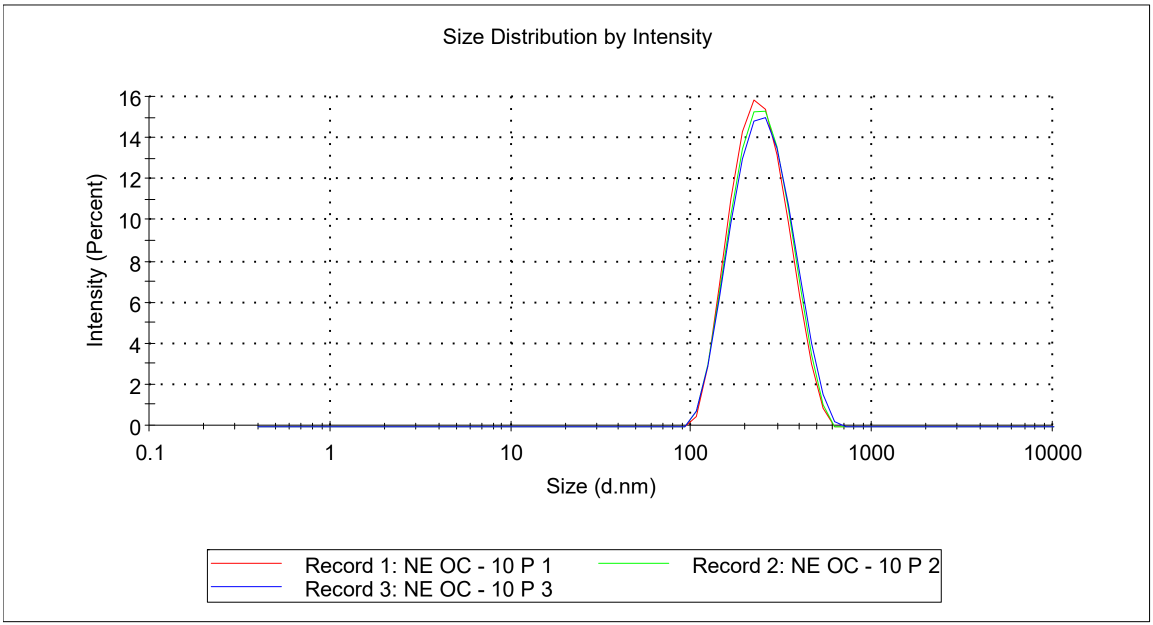

Size Quality Report

v2.0

Malvern Instruments Ltd - © Copyright 2008

Sample Name: NE OC - 10 P 1

SOP Name: mansettings.nano

File Name: NE OC - 10 P.dts

Record Number: 1

Measurement Date and Time: quarta-feira, 31 de julho de 2019 17:43:42

Temperature (°C): 25,0

RESULT MEETS QUALITY CRITERIA

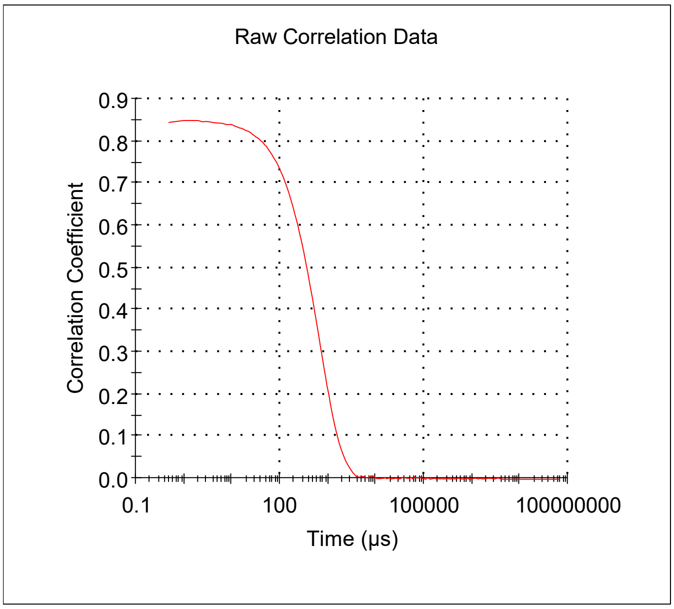

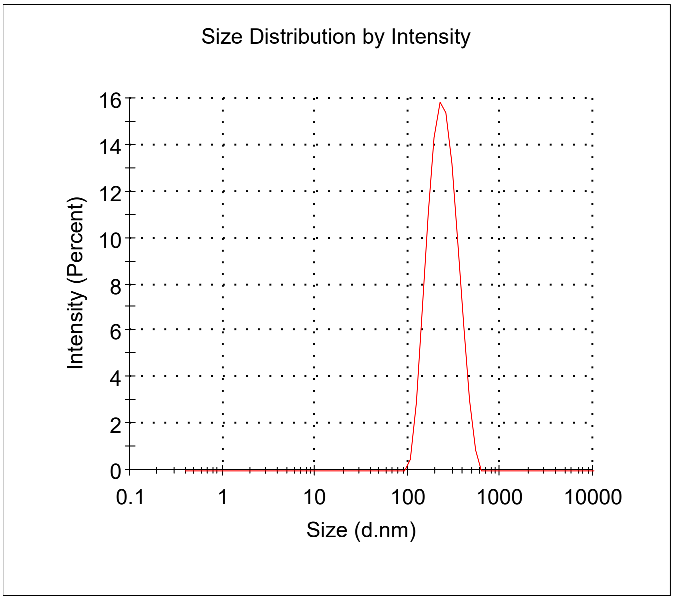

Correlogram Report

v2.0

Malvern Instruments Ltd - © Copyright 2008

Sample Details

Sample Name: NE OC - 10 P 1

SOP Name: mansettings.nano

General Notes:

**File Name:**	NE OC - 10 P.dts	**Dispersant Name:**	Water
**Record Number:**	1	**Dispersant RI:**	1,330
**Material RI:**	1,50	**Viscosity (cP):**	0,8872
**Material Absorbtion:**	0,000	**Measurement Date and Time:**	quarta-feira, 31 de julho ...

System

**Temperature (°C):**	25,0	**Duration Used (s):**	50
**Count Rate (kcps):**	244,0	**Measurement Position (mm):**	4,65
**Cell Description:**	Disposable sizing cuvette	**Attenuator:**	8

Results

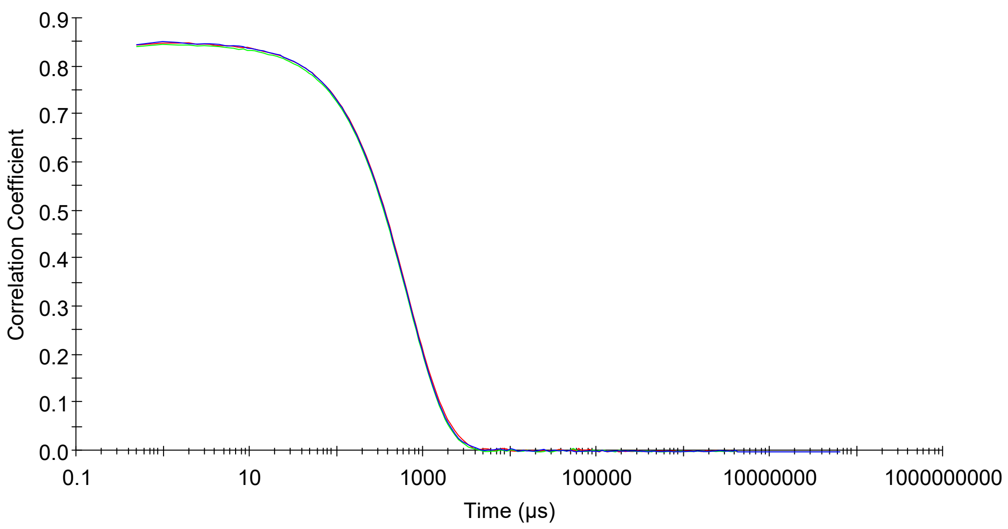

Correlogram Report

v2.0

Malvern Instruments Ltd - © Copyright 2008

Sample Details

Sample Name: NE Copaiba+folha 1

SOP Name: mansettings.nano

General Notes:

**File Name:**	NE Copaiba+folha.dts	**Dispersant Name:**	Water
**Record Number:**	1	**Dispersant RI:**	1,330
**Material RI:**	1,50	**Viscosity (cP):**	0,8872
**Material Absorbtion:**	0,000	**Measurement Date and Time:**	sexta-feira, 12 de julho d...

System

**Temperature (°C):**	25,0	**Duration Used (s):**	50
**Count Rate (kcps):**	258,1	**Measurement Position (mm):**	4,65
**Cell Description:**	Disposable sizing cuvette	**Attenuator:**	8

Results

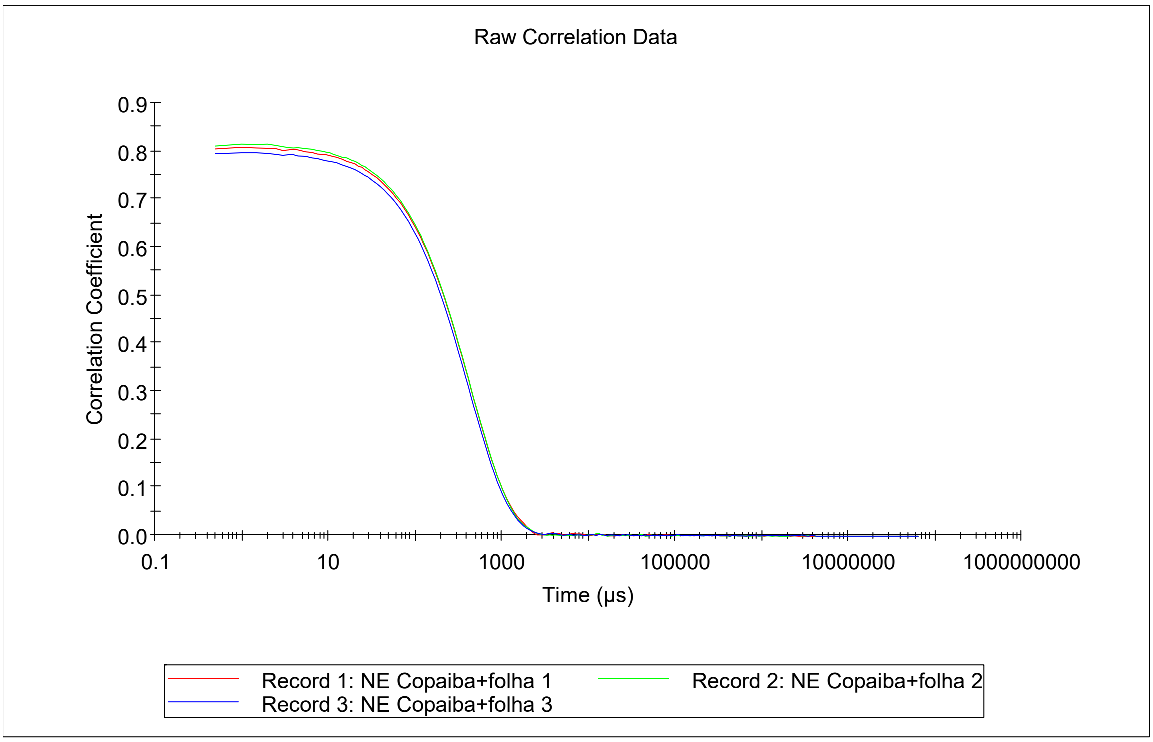

Size Distribution Report by Intensity

v2.2

Sample Details

Sample Name: NE Copaiba+folha 1

SOP Name: mansettings.nano

General Notes:

**File Name:**	NE Copaiba+folha.dts	**Dispersant Name:**	Water
**Record Number:**	1	**Dispersant RI:**	1,330
**Material RI:**	1,50	**Viscosity (cP):**	0,8872
**Material Absorbtion:**	0,000	**Measurement Date and Time:**	sexta-feira, 12 de julho de 2...

System

**Temperature (°C):**	25,0	**Duration Used (s):**	50
**Count Rate (kcps):**	258,1	**Measurement Position (mm):**	4,65
**Cell Description:**	Disposable sizing cuvette	**Attenuator:**	8

Results

			Size (d.n...	% Intensity:	St Dev (d.n...
**Z-Average (d.nm):**	149,2	**Peak 1:**	176,5	100,0	75,67
**PdI:**	0,159	**Peak 2:**	0,000	0,0	0,000
**Intercept:**	0,898	**Peak 3:**	0,000	0,0	0,000

Result quality Good

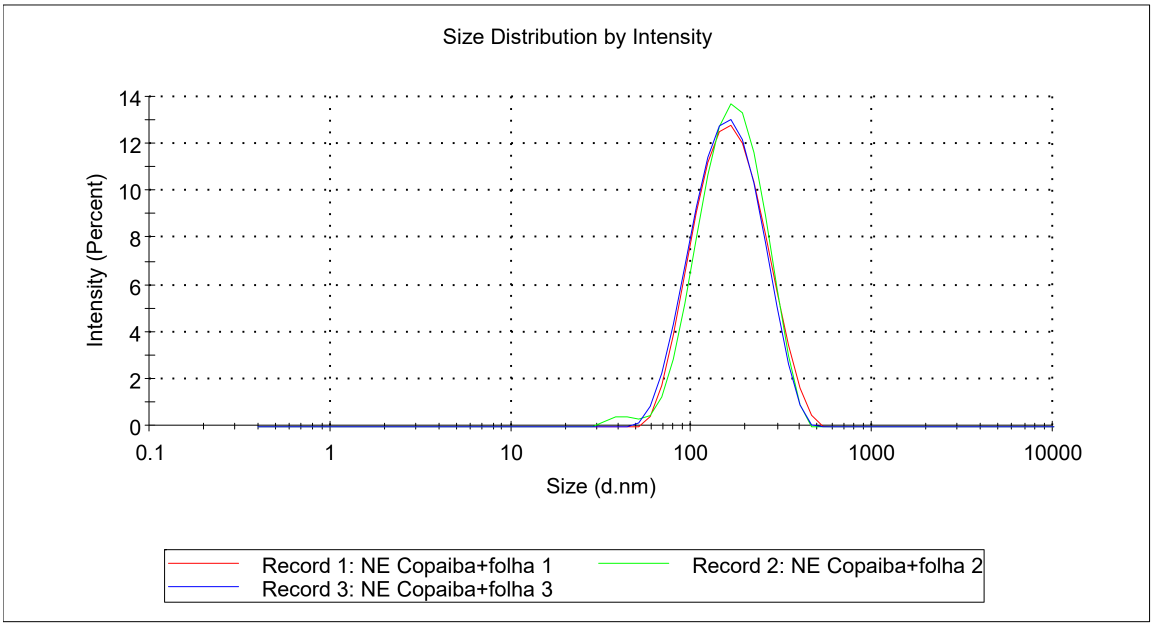

Size Quality Report

v2.0

Malvern Instruments Ltd - © Copyright 2008

Sample Name: NE Copaiba+folha 1

SOP Name: mansettings.nano

File Name: NE Copaiba+folha.dts

Record Number: 1

Measurement Date and Time: sexta-feira, 12 de julho de 2019 12:58:39

Temperature (°C): 25,0

RESULT MEETS QUALITY CRITERIA

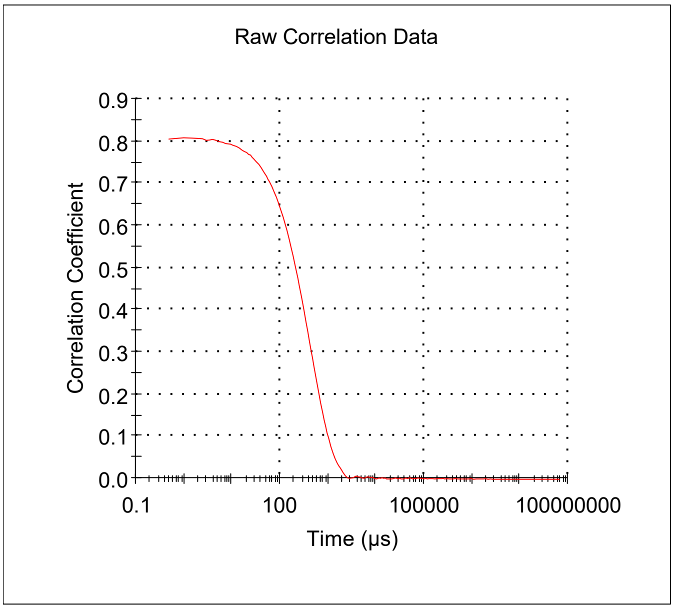

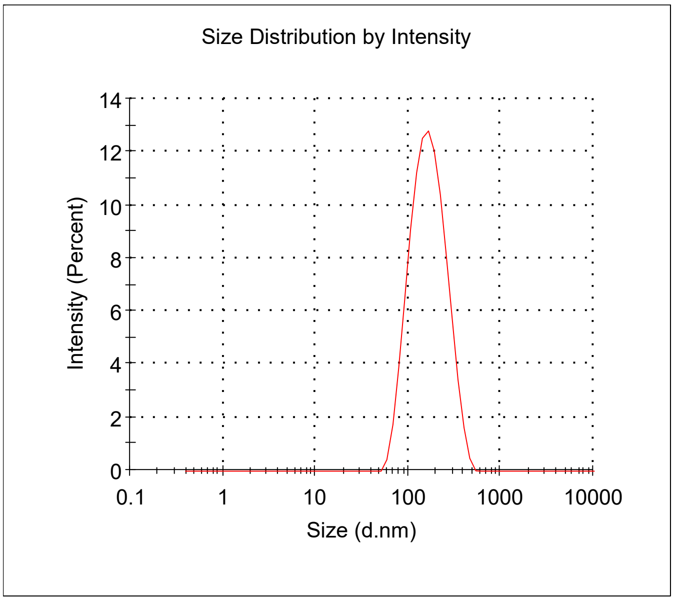

Supplement: Supplementary file 1 [file plants-15-02901-s001.zip › Supplementary_Data_S2_DLS_Processing_Optimization_and_Final_BSLE-NE.pdf]
